# Deciphering Alzheimer’s disease transcriptomics: exploration and validation of core genes in tau and Aβ pathological models toward novel therapeutic targets

**DOI:** 10.3389/fnagi.2025.1621153

**Published:** 2025-10-10

**Authors:** Hui E. Zhang, Meng Li Xiao, Jin Jin Ji, Yu Rong Cheng, Fang Lu

**Affiliations:** ^1^Institute of Clinical Pharmacology, Xiyuan Hospital, China Academy of Chinese Medical Sciences, Beijing, China; ^2^Beijing University of Chinese Medicine, Beijing, China

**Keywords:** Alzheimer’s disease, microarray data, eQTL analysis, Mendelian randomization, qRT-PCR

## Abstract

**Introduction:**

To decode the pathology of Alzheimer’s disease (AD), this study employs multi-omics approaches and bioinformatics analyses to explore AD-associated differentially expressed genes (DEGs), dissect the underlying mechanisms, and thereby facilitate the identification of core genes as well as the development of targeted therapeutic strategies.

**Methods:**

Six independent AD datasets were collected from the Gene Expression Omnibus (GEO) database, and data were processed and normalized using the R software. The evaluation of relationships between differentially expressed genes (DEGs) and AD encompassed differential expression analysis, expression quantitative trait loci (eQTL) analysis, and Mendelian randomization (MR) analysis. Additionally, gene set enrichment analysis (GSEA), immune cell correlation analysis, and Gene Ontology (GO)/Kyoto Encyclopedia of Genes and Genomes (KEGG) enrichment analyses were employed to investigate the functional roles and pathways of these genes. Machine learning approaches were applied to identify potential genes from differentially expressed genes (DEGs) associated with AD. The diagnostic performance of these candidate genes was assessed using a nomogram and receiver operating characteristic curves. The expression levels of the identified genes were further validated via quantitative real-time polymerase chain reaction (qRT-PCR).

**Results:**

Differential gene analysis identified 294 highly expressed genes and 330 lowly expressed genes, and MR analysis identified 10 significantly co-expressed genes associated with AD, specifically METTL7A, SERPINB6, VASP, ENTPD2, CXCL1, FIBP, FUCA1, TARBP1, SORCS3, and DMXL2. Noteworthy observations naive CD4^+^ T cells in AD, with this distinct from CIBERSORT analysis included the presence of unique immune cell subset further underscoring the critical role of immune processes in the pathogenesis and progression of the disease. METTL7A, SERPINB6, VASP, ENTPD2, FIBP, FUCA1, TARBP1, SORCS3, and DMXL2 were selected for nomogram construction and machine learning-based assessment of diagnostic value, demonstrating considerable diagnostic potential. Furthermore, the significance of the identified key genes was corroborated using both the GEO validation set and qRT-PCR.

**Conclusion:**

METTL7A, SERPINB6, VASP, ENTPD2, FIBP, FUCA1, TARBP1, SORCS3, and DMXL2 may regulate the progression of AD. These findings not only deepen our mechanistic understanding of AD pathology but also provide potential candidate genes for the development of targeted therapeutic strategies against AD.

## 1 Introduction

Alzheimer’s disease (AD), a progressive neurodegenerative disorder, primarily impairs cognitive functions in older adults, manifesting as gradual memory loss, deteriorating thinking abilities, and diminished capacity for daily activities (Cairns et al., 2020; Freyssin et al., 2020). With the accelerating global aging trend, AD has emerged as a critical public health challenge. Current estimates indicate over 50 million individuals worldwide live with AD, a number projected to double by 2050 (GBD 2019 Dementia Forecasting Collaborators, 2022). Beyond causing profound suffering for patients, AD imposes substantial economic burdens on families and societies (Jia et al., 2018). Although its exact etiology remains unclear, multifactorial mechanisms involving genetic susceptibility, environmental influences, and lifestyle factors are widely implicated (Zhang et al., 2024). Biochemically, AD is characterized by β-amyloid plaque accumulation and neurofibrillary tangle formation, pathological hallmarks driving neurodegeneration and cognitive decline (John and Reddy, 2021; Tzioras et al., 2023).

Alzheimer’s disease is strongly linked to rare mutations in APP, PSEN1, and PSEN2 genes (Zhang et al., 2019), while the APOE allele represents the strongest genetic risk factor for sporadic AD (Huynh et al., 2017). Genome-wide association studies (GWAS) and whole-genome sequencing (WGS) have identified additional risk loci for late-onset AD, including TREM2, BIN1, CLU, ABCA7, and CR1 (Schupf et al., 2015). A meta-analytic approach further pinpointed susceptibility regions such as HLA-DRB5-HLA-DRB1, PTK2B, and SORL1, underscoring the polygenic architecture of AD (Farfel et al., 2016). Neuroinflammation, a cardinal pathological feature, involves microglial and astrocytic activation (Guo et al., 2020). Soluble oligomeric Aβ (oAβ) modulates glial responses through receptors like TREM2, LRP1, and TLR4, potentially enhancing phagocytic clearance of oAβ (Zhao et al., 2018). Pathological tau species, conversely, trigger microglial inflammatory cascades, promoting cytokine release that exacerbates tau hyperphosphorylation via feedback mechanisms on neuronal signaling (McQuade et al., 2020).

**FIGURE 1:**
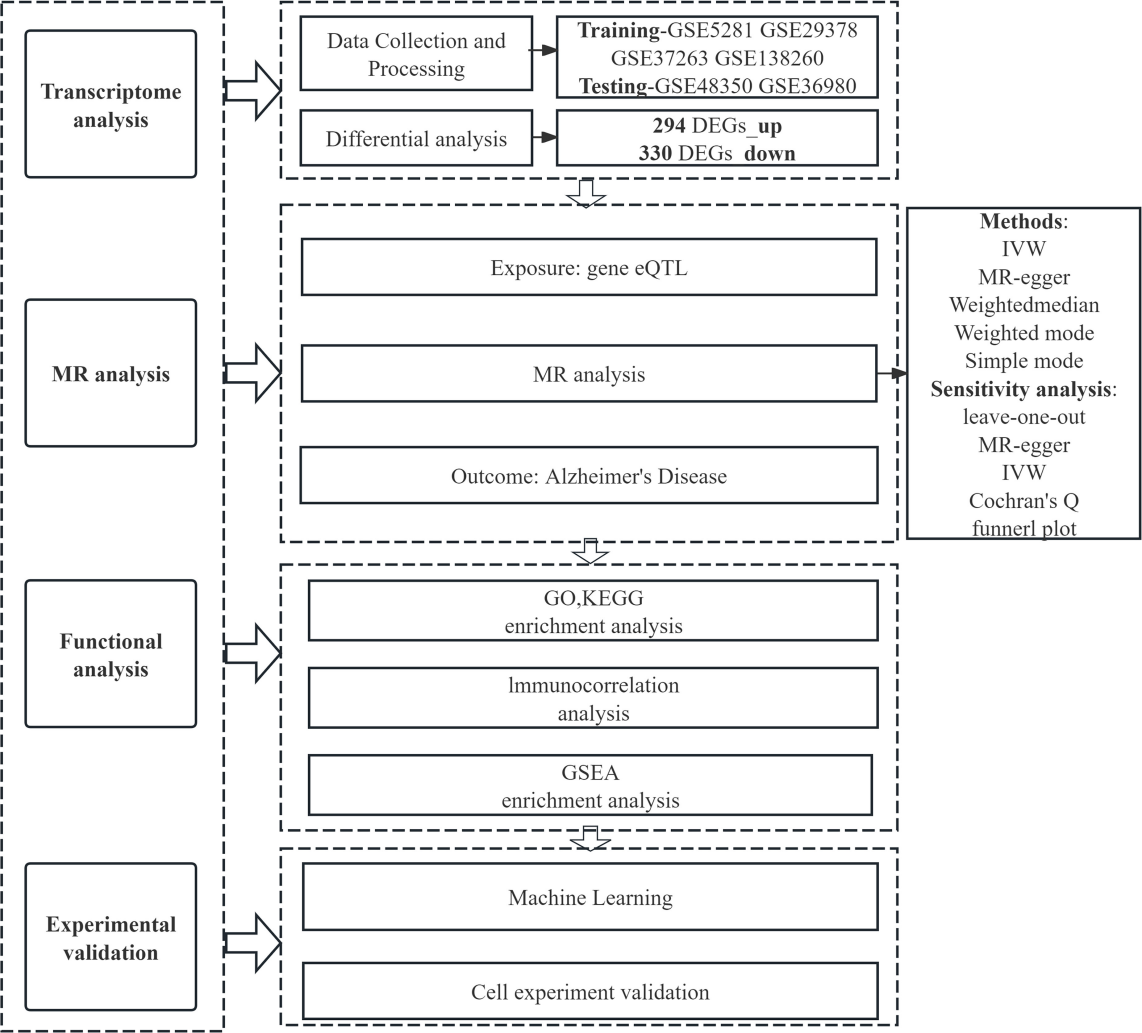
Workflow diagram of this study.

In this study, we employed MR to investigate correlations between eQTL data and AD genome-wide association study (GWAS) data. Furthermore, AD-associated genes were precisely localized using data from the GEO database. Functional characterization of AD-related DEGs was performed via GO analysis, KEGG pathway analysis, and GSEA. Cellular immune infiltration analysis was applied to explore the association between the expression levels of AD-related key genes and infiltrating immune cells. Finally, we validated the differential expression of these key genes using machine learning approaches, *in vitro* cell models, and external GEO datasets—findings that collectively enhance the reliability of our results. The overarching aim of this study was to precisely identify core regulatory genes involved in AD pathology and facilitate the development of intervention strategies related to AD (Figure 1).

## 2 Materials and methods

### 2.1 Data collection on Alzheimer’s disease

Gene expression datasets and clinical phenotype data matching the search criteria “Alzheimer’s disease,” “human,” and “gene expression” were acquired through microarray dataset analysis. All gene expression profiles and corresponding platform probe annotations are publicly accessible for download from the Gene Expression Omnibus (GEO) database^1^ (Zhu et al., 2020).

### 2.2 Identification of differential genes

Using R software (version 4.3.2), we performed dataset-specific preprocessing for GSE5281, GSE29378, GSE37263, and GSE138260, which involved data reading and initial normalization using gene expression matrices and annotation files downloaded from the GEO database. After individual preprocessing, the datasets were merged to combine 134 normal samples and 142 Alzheimer’s disease (AD) samples, followed by batch effect correction and variance-stabilizing transformation. Differential gene screening was conducted using the “limma” package with empirical Bayesian analysis, applying significance thresholds of *P* < 0.05 and absolute log2 fold change (logFC) > 0.585 (Yang et al., 2020). The “pheatmap” package was utilized to generate visualizations, including volcano plots for differential expression analysis and heatmaps for clustering patterns of significant genes. Principal component analysis (PCA) was performed via the “prcomp” function to evaluate sample clustering, assess batch effect mitigation, and visualize key gene expression signatures distinguishing AD cases from healthy controls (Li et al., 2019; Vacchio et al., 2019). This integrated analytical pipeline ensured robust data normalization, rigorous statistical testing, and comprehensive visualization of molecular markers associated with Alzheimer’s disease.

### 2.3 GO and KEGG enrichment analysis

Differential genes were analyzed by GO functional annotation and KEGG pathway enrichment using the “clusterProfiler” R software package (Yang-Chun et al., 2020), and the filtering criterion was set at *P* < 0.05 to understand the potential functional pathways and pathogenesis (Lu et al., 2020).

### 2.4 eQTL analysis of exposure data

To identify genetic variants associated with gene expression, we conducted eQTL analysis using transcriptomic and genotypic data from multiple cohorts. Specifically, peripheral blood eQTL data comprising 5,311 European individuals were incorporated (Westra et al., 2013). The aggregated eQTL dataset utilized in this study was obtained from the GWAS Catalog website^2^ (Cao et al., 2022). Employing the R package “TwoSampleMR,” we identified single-nucleotide polymorphisms (SNPs) with strong statistical associations (*P* < 5 × 10^8^) to serve as instrumental variables. Stringent linkage disequilibrium (LD) parameters were applied, setting the LD threshold at r^2^ < 0.001 and defining an aggregation distance of 10,000 kb (Wootton et al., 2020). SNPs exhibiting weak trait associations or insufficient explanatory power for phenotypic variance were excluded through filtering based on an F-test value > 10 (Rosoff et al., 2021), ensuring only robust genetic instruments were retained for subsequent analyses.

### 2.5 Outcome data set

The outcome data were sourced from the Genetic Association Database (see text footnote 2) within the GWAS Summary Dataset (IEU) (Wu et al., 2020). The specific GWAS identifier utilized was ebi-a-GCST90027158, which included 39,106 case samples and 46,828 control samples from European pedigree populations, encompassing a total of 20,921,626 single-nucleotide polymorphisms (SNPs). All GWAS summary statistics employed in this study are publicly accessible and available for free download.

### 2.6 Mendelian randomization analysis

Mendelian randomization (MR) analysis was conducted using the TwoSampleMR software package. To explore causal associations between Alzheimer’s disease and differentially expressed genes, we employed inverse variance weighting (IVW), MR-Egger, simple mode, weighted median, and weighted mode methods, complemented by sensitivity analyses (Chen et al., 2020). Co-expressed genes—including both upregulated and downregulated transcripts—were identified by intersecting disease-associated gene sets with differentially expressed gene lists. Subsequently, individual MR analyses were performed for each gene in this intersection to determine its causal relationship with Alzheimer’s disease. These analyses incorporated heterogeneity testing, multiple validity assessments, and leave-one-out sensitivity analysis to evaluate result robustness and reliability (Nie et al., 2020).

### 2.7 Immune cell analysis

Immune cell infiltration profiles in the AD and normal tissue samples from the GEO AD dataset were quantified using the “LM22” signature matrix and the “CIBERSORT” algorithm in R software (Zhu et al., 2019). Statistical significance of differences in immune cell proportions between groups was evaluated with 1,000 permutations, and a *P*-value < 0.05 was set as the threshold for meaningful results. Visualization of immune cell infiltration patterns was accomplished by generating violin plots and heatmaps using the “pheatmap” and “ggplot2” packages, which clearly displayed the distribution of 22 immune cell subsets across samples. For immune correlation analysis, the Spearman correlation coefficient was calculated to assess the associations between infiltrating immune cell subsets using the “corrplot” package (Li J. et al., 2025). Meanwhile, the relationship between immune cell infiltration levels and the expression of immune checkpoint genes was explored through scatter plots and linear regression analysis with the “ggpubr” package, and statistical significance was determined by adjusting for multiple comparisons using the Benjamini-Hochberg method (FDR < 0.05) (Wang, 2025).

### 2.8 GSEA enrichment analysis

Single-gene GSEA enrichment analysis is a common method used to assess the enrichment of individual genes in a dataset (Bourdely et al., 2020). Instead of relying on differential genes, this method takes an enrichment perspective of the dataset by considering each gene in the expression matrix, ranking the genes according to a specific metric, and then checking whether the genes in the dataset are enriched at the top or bottom of the ranked list (Cai et al., 2019). Single-gene GSEA enrichment analysis is a powerful analytical tool that provides a more comprehensive assessment of the enrichment of all genes in a dataset, thus providing a deeper understanding of gene expression data (Sande-Melón et al., 2019). In this study, in order to more comprehensively explore the potential regulatory mechanism of each co-expressed gene in AD, we employed GSEA (Gene Set Enrichment Analysis) enrichment analysis and visualization in R, and selected “C2: KEGG gene sets” as the database (Li and Guo, 2020), and then performed single gene GSEA enrichment analysis for each co-expressed gene. *P* < 0.05 was considered as significant enrichment.

### 2.9 Identifying core genes for AD via machine learning

This study employed three machine learning algorithms—random forests (RF), least absolute shrinkage and selection operator (LASSO) logistic regression, and support vector machine-recursive feature elimination (SVM-RFE)—to screen for the characteristic genes of AD (Li G. et al., 2025). Specifically, the RF algorithm was implemented using the “randomForest” package in R software, LASSO logistic regression analysis was conducted via the “glmnet” package in R software, and the SVM-RFE algorithm was executed with the “e107” package in R software (Engebretsen and Bohlin, 2019). AD-related differentially expressed genes (DEGs) were obtained by taking the intersection of the characteristic genes identified by the RF, LASSO logistic regression, and SVM-RFE algorithms. Furthermore, the efficacy of these common AD-related DEGs in diagnosing AD was evaluated using the receiver operating characteristic (ROC) curve.

### 2.10 Cell culture

SK-N-SH cells were cultured in Dulbecco’s Modified Eagle Medium (DMEM), which was supplemented with 10% fetal bovine serum (FBS) and 1% each of penicillin and streptomycin. All cell cultures were maintained in a humidified incubator at 37°C with 5% CO_2_ (Goerges et al., 2024). To establish *in vitro* models that mimic distinct pathological features of AD, the SK-N-SH cells underwent two separate treatment regimens. First, the cells were incubated with 20 nmol/L okadaic acid for 48 h to simulate AD-related tau pathology (Boban et al., 2019). The second regimen involved exposure to 10 μmol/L amyloid-β 1-42 (Aβ_1–42_) oligomers for 24 h to simulate AD-associated Aβ pathology (Han et al., 2017).

### 2.11 qRT-PCR

Total RNA was extracted from treated SK-N-SH cells using the TRIzol^®^ Plus RNA Purification Kit (Thermo Fisher, United States, REF:12183-555). The purity and concentration of the RNA was determined and then reverse transcribed to cDNA using the RNase-Free DNase Set (Qiagen, Shanghai, China, REF:79254). It was then processed using the Start-up reagent: SuperScriptIII First-Strand Synthesis SuperMix (Thermo Fisher, United States, REF:11752-050) and Power SYBR^®^ Green PCR Master Mix (Applied Biosystems, United States, REF:4367659). Finally, PCR was conducted on the CFX384 instrument (Bio-Rad, United States). The β-actin primer pairs was used as the internal control (Wang et al., 2020). The primer sequences used are shown in Table 1.

**TABLE 1:** Primer sequences used in quantitative real-time polymerase chain reaction (qRT-PCR).

Gene	Primer direction	Sequence
METTL7A	Forward	GTGCTCTGTGAAGAACCAGGAG
	Reverse	GATCCAGGACTTGTTGCCAGAAG
SERPINB6	Forward	AGGGAAACACCGCTGCACAGAT
	Reverse	GTGCCAGTCTTGTTCACTTCGG
VASP	Forward	CTGGGAGAAGAACAGCACAACC
	Reverse	AGGTCCGAGTAATCACTGGAGC
ENTPD2	Forward	GGAGAACGACACAGGCATTGTG
	Reverse	CCCCAGAAGGGTTGTCTGCAT
FIBP	Forward	CAAGGTGGTAGAGGAAATGCGG
	Reverse	CCTGTCTCAAAGCGGTTGTTAGC
FUCA1	Forward	GACTTCGGACCGCAGTTCACTG
	Reverse	CCAGTTCCAAGACACAGGACTC
TARBP1	Forward	GATGGTCTTGCTGGCTGTGGAT
	Reverse	GCATCTGTCAGTCTTCAGCAAGG
SORCS3	Forward	AGGCAGGAATGGAGACCCACAT
	Reverse	CCAGGTCTGATAGTCCTCCTTG
DMXL2	Forward	GCTTTGGCTGATACAGTGGCTAC
	Reverse	GGCAGCGATGTCAAAAGGCATG
β-Actin	Forward	GATGACCCAGATCATGTTTGAGAC
	Reverse	GGAGTCCATCACGATGCCAGT

## 3 Results

### 3.1 GEO datasets processing

**FIGURE 2:**
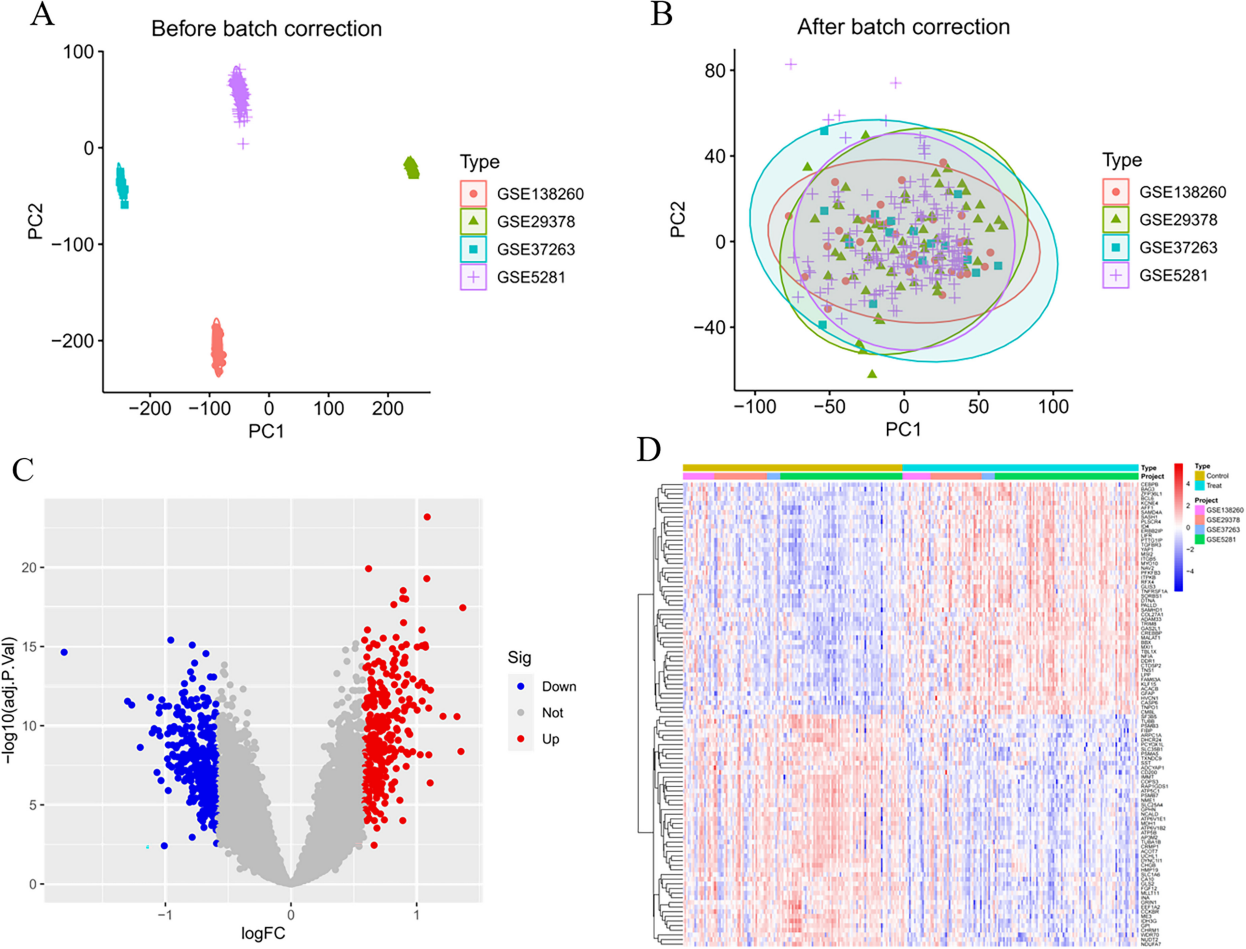
Batch correction and variance analysis. **(A)** Before the batch correction. **(B)** After the batch correction. **(C)** Volcano plot of differential expression genes. **(D)** Heatmap of differential expression genes.

Four Alzheimer’s disease microarray datasets were retrieved from the GEO database as experimental datasets. The four datasets comprised 142 Alzheimer’s disease patients and 134 healthy controls in total. Details of the included datasets are provided in Table 2. Using R version 4.3.2, we performed normalization and integration of gene expression values across respective datasets and mitigated batch effects via principal component analysis (PCA). As illustrated in Figure 2A, pronounced batch effects were evident among the four Alzheimer’s disease gene datasets prior to correction. Following normalization and PCA-based batch effect adjustment, all samples within the integrated dataset exhibited satisfactory homogeneity, as demonstrated in the post-correction PCA analysis shown in Figure 2B.

**TABLE 2:** Characteristics of the four datasets.

GSE ID	Sample	Tissue	Platform
GSE5281	87 samples 74 controls	Brain	GPL570-55999
GSE29378	31 samples 32 controls	Brain	GPL6947-13512
GSE37263	8 samples 8 controls	Brain	GPL5175-3188
GSE138260	17 samples 19 controls	Brain	GPL27556-55246

### 3.2 Differential genes identification

In the analytical results, smaller *P*-values indicated stronger statistical significance for both gene sequencing consistency and differential gene expression. Overall, we identified 294 significantly upregulated and 330 significantly downregulated differentially expressed genes (DEGs). Supplementary Table 1 lists detailed annotations for these DEGs, including gene symbols, entrez IDs, and adjusted *P*-values. Figures 2C, D display the top 50 upregulated and top 50 downregulated DEGs, respectively, ranked by absolute fold-change values.

### 3.3 Selection of Mendelian randomization instrument variables

**FIGURE 3:**
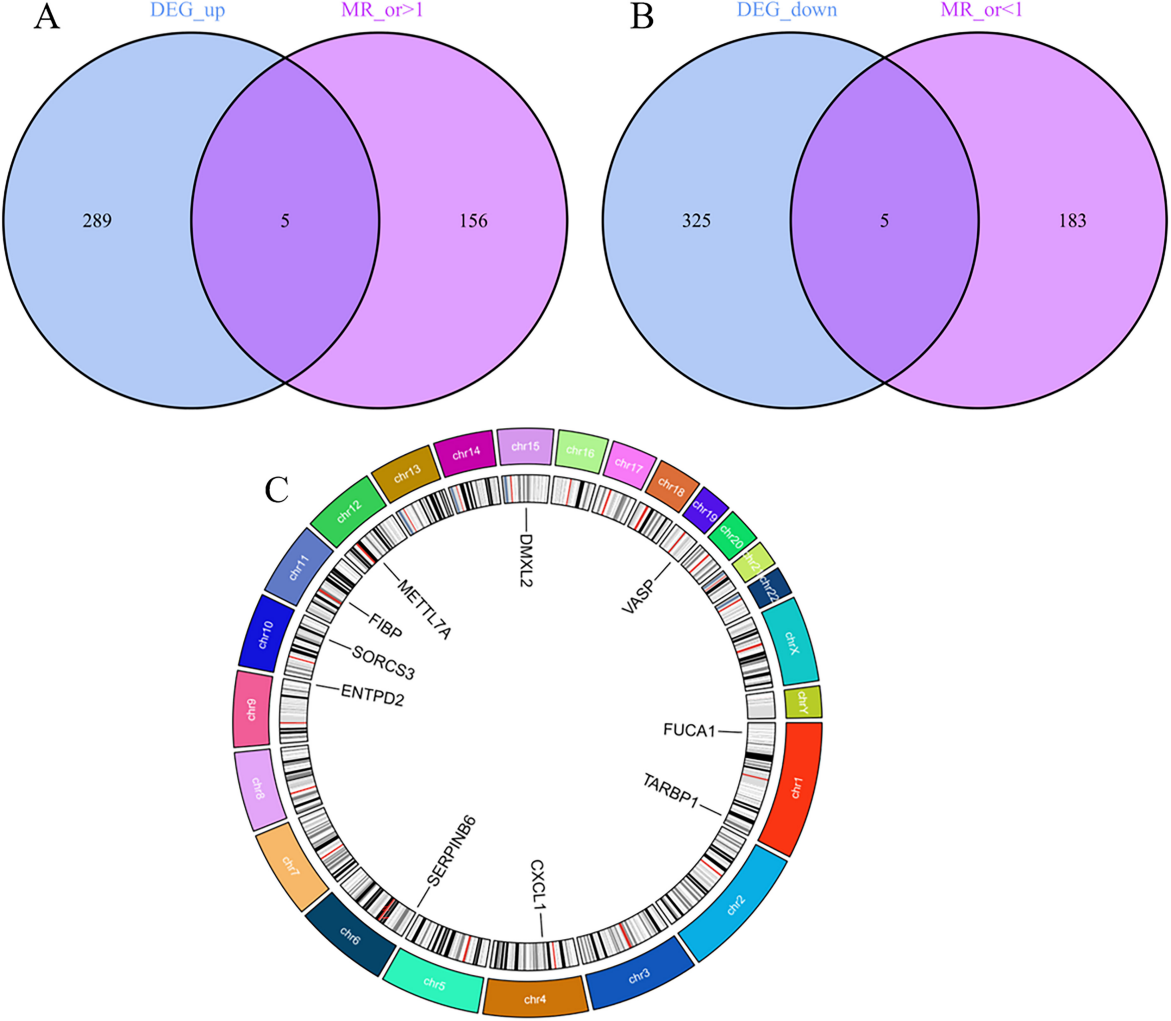
Screening and localization of critical genes. **(A)** Disease upregulated differentially expressed genes (DEGs) are intersected with genes with OR values greater than one in the Mendelian randomization (MR) results. **(B)** Disease downregulated DEGs are intersected with genes with OR values less than one in the MR results. **(C)** Position of disease-critical genes on human chromosomes.

**FIGURE 4:**
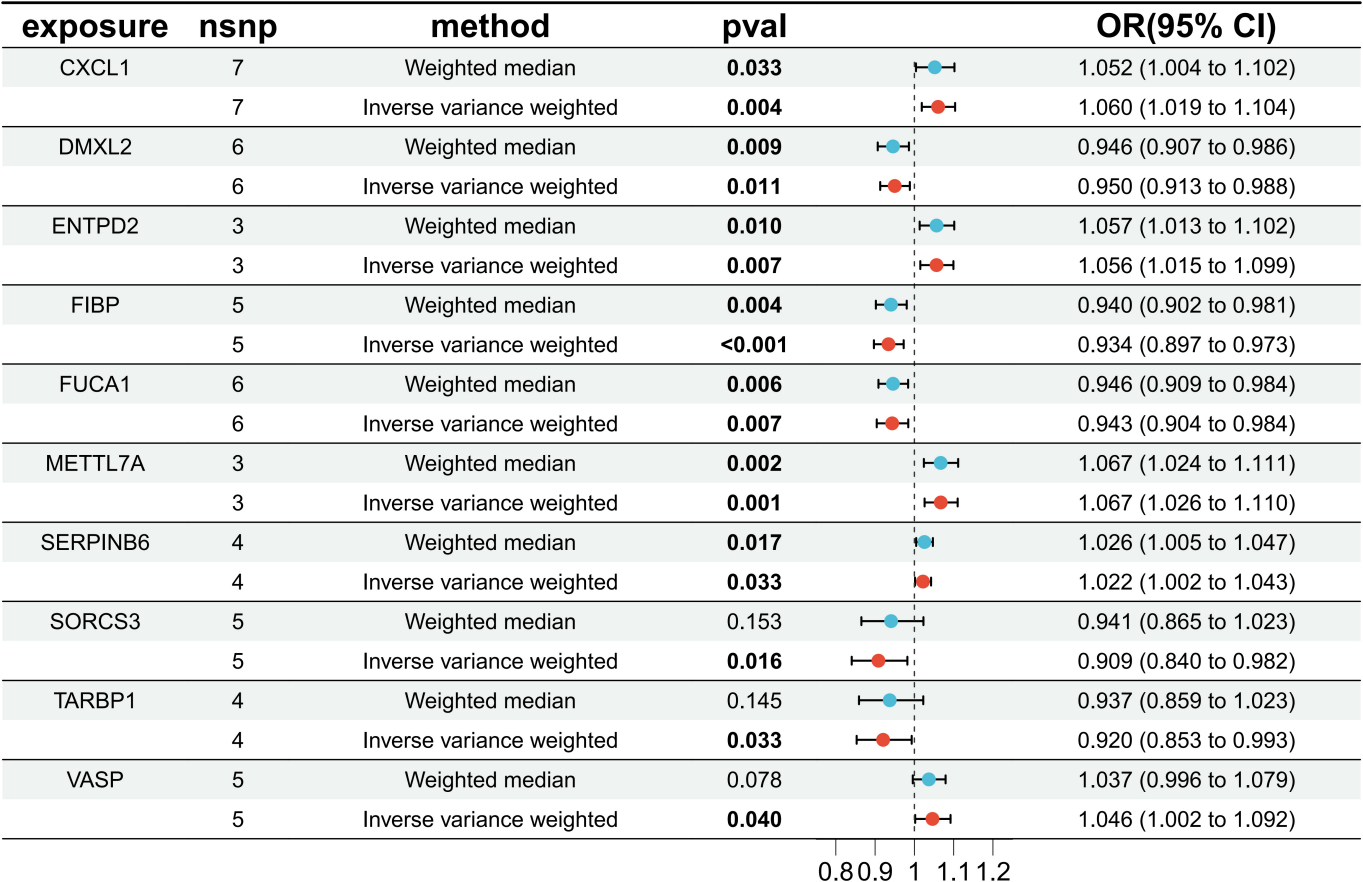
Disease critical genes causally associated with Alzheimer’s disease (AD).

Through cross-tabulation analysis, we identified co-expressed genes from the intersection of related genes and differentially expressed genes, comprising five up-regulated genes (METTL7A, SERPINB6, VASP, ENTPD2, and CXCL1) and five down-regulated genes (FIBP, FUCA1, TARBP1, SORCS3, and DMXL2), as illustrated in Figures 3A, B. To further characterize the chromosomal localization of these genes, we generated a visualization of the co-expressed gene distribution across the genome (Figure 3C). Subsequently, we conducted a MR analysis on the 10 genes co-expressed with AD to evaluate the causal effects of each gene on the disease. The results indicated that all five upregulated co-expressed genes exhibited a significant positive causal association with AD in the MR analysis using the inverse-variance weighting method. Specifically, five upregulated co-expressed genes exhibited significant positive associations with Alzheimer’s disease: METTL7A (OR = 1.067; 95% CI: 1.026–1.110; *P* = 0.001), SERPINB6 (OR = 1.022; 95% CI: 1.002–1.043; *P* = 0.033), VASP (OR = 1.046; 95% CI: 1.002–1.092; *P* = 0.040), ENTPD2 (OR = 1.015; 95% CI: 1.015–1.099; *P* = 0.007), and CXCL1 (OR = 1.060; 95% CI: 1.019–1.104; *P* = 0.004). Conversely, all five downregulated co-expressed genes showed significant negative causal associations with the disease: FIBP (OR = 0.934; 95% CI: 0.897–0.973; *P* = 0.001), FUCA1 (OR = 0.943; 95% CI: 0.904–0.984; *P* = 0.007), TARBP1 (OR = 0.920; 95% CI: 0.853–0.993; *P* = 0.033), SORCS3 (OR = 0.909; 95% CI: 0.840–0.982; *P* = 0.016), and DMXL2 (OR = 0.950; 95% CI: 0.913–0.988; *P* = 0.011). Beyond the MR-Egger approach, additional validation analyses were conducted employing simple mode, weighted median, and weighted mode methodologies. For the five upregulated genes, all analytical methods consistently revealed an elevated risk of Alzheimer’s disease, as evidenced by odds ratios (ORs) greater than 1. Conversely, across all applied methods, the five downregulated genes consistently indicated a reduced risk of Alzheimer’s disease, with ORs consistently below 1 (Figure 4). The heterogeneity and pleiotropy tests for co-expressed genes yielded non-significant results (all *P*-values > 0.05), indicating no statistical evidence of heterogeneity or pleiotropic effects that would necessitate adjustment for these biases. Results from the leave-one-out sensitivity analysis demonstrated consistency between the effect estimates when each instrumental variable was excluded individually and the overall combined effect size, confirming the robustness of the analytical framework.

### 3.4 Gene sensitivity analysis and differential expression verification

**FIGURE 5:**
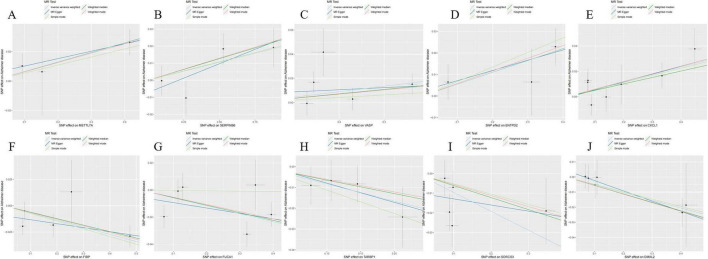
Scatterplot of Mendelian randomization (MR) analysis of the association between Alzheimer’s disease (AD) critical genes and AD. **(A)** Scatterplot of MR analysis of METTL7A. **(B)** Scatterplot of MR analysis of SERPINB6. **(C)** Scatterplot of MR analysis of VASP. **(D)** Scatterplot of MR analysis of ENTPD2. **(E)** Scatterplot of MR analysis of CXCL1. **(F)** Scatterplot of MR analysis of FIBP. **(G)** Scatterplot of MR analysis of FUCA1. **(H)** Scatterplot of MR analysis of TARBP1. **(I)** Scatterplot of MR analysis of SORCS3. **(J)** Scatterplot of MR analysis of DMXL2.

**FIGURE 6:**
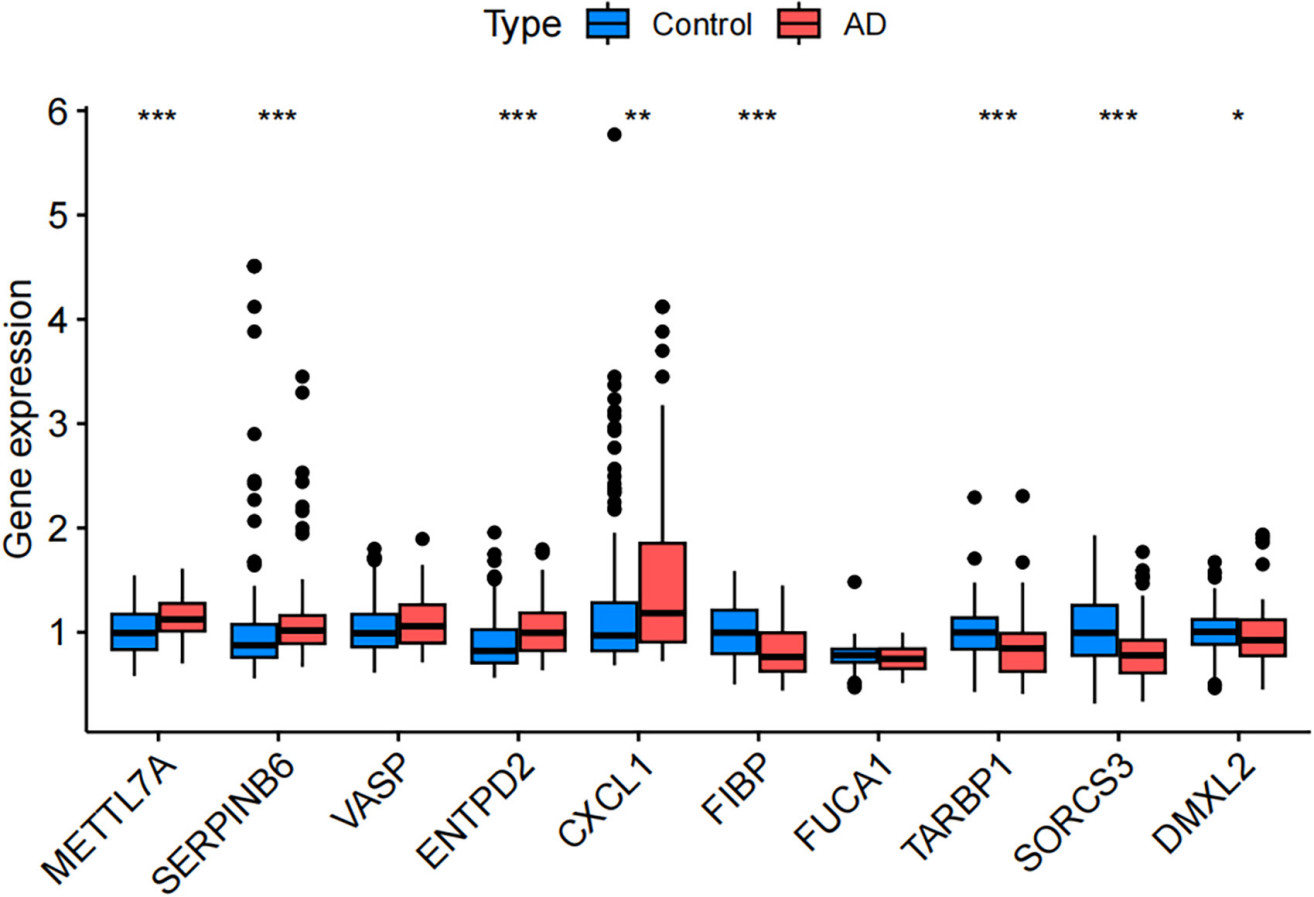
Validation of differential expression of disease-related key genes in the Gene Expression Omnibus (GEO) validation dataset (GSE48350). (Control, the healthy control group; AD, the Alzheimer’s disease patient group. *P < 0.05, **P < 0.01, ***P < 0.001).

Sensitivity analyses were conducted on 10 key AD genes using MR-Egger regression and Cochran’s test. The results indicated no heterogeneity or pleiotropy, thus confirming the reliability of the findings (Table 3). The analysis of the funnel plot indicated that no individual single-nucleotide polymorphism (SNP) affected the outcome, implying the absence of directional pleiotropy for individual SNP non-violation and bias estimation. The leave-one-out analysis further confirmed the absence of horizontal pleiotropy, thereby demonstrating the robustness and reliability of the analytical methods and results (Figure 5). Furthermore, the present study examined the variations in expression of 10 pivotal genes in AD by utilizing the validation set GSE48350 dataset. The findings indicated notable distinctions in the levels of expression of these critical genes in AD, thereby validating their differential expression (Figure 6).

**TABLE 3:** Sensitivity analysis of Alzheimer’s disease (AD) critical genes.

Gene	*P*MR−Egger	*P*MR−Egger. Q	*P*IVW. Q
METTL7A	0.724	0.820	0.876
SERPINB6	0.513	0.299	0.367
VASP	0.664	0.163	0.239
ENTPD2	0.857	0.452	0.734
CXCL1	0.902	0.454	0.581
FIBP	0.266	0.440	0.335
FUCA1	0.652	0.078	0.113
TARBP1	0.940	0.776	0.915
SORCS3	0.384	0.293	0.286
DMXL2	0.525	0.996	0.985

### 3.5 GO and KEGG enrichment analysis

**FIGURE 7:**
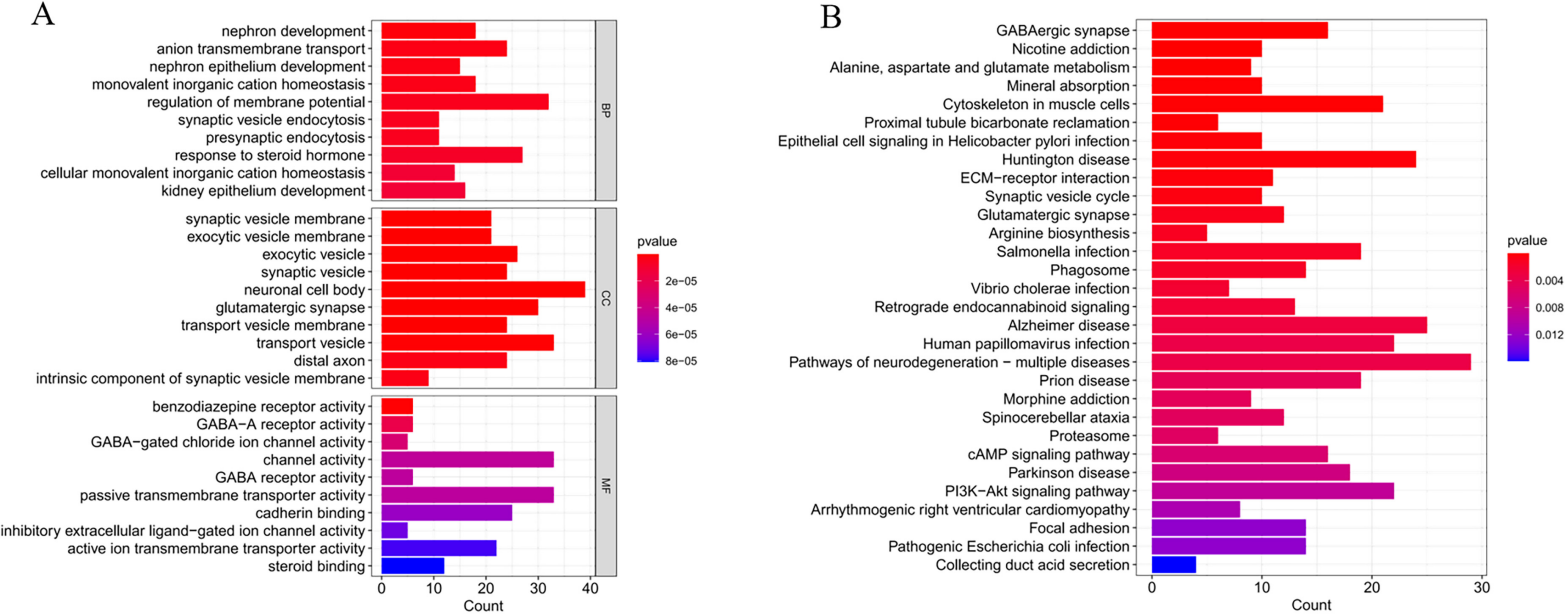
Functional enrichment analysis of critical genes. **(A)** Gene Ontology (GO) enrichment analysis of Alzheimer’s disease (AD) critical genes. **(B)** Kyoto Encyclopedia of Genes and Genomes (KEGG) enrichment analysis of AD critical genes.

After the screening process, we successfully identified 624 genes associated with AD. To delve deeper into the potential functions of these differentially expressed genes, we performed GO and KEGG enrichment analyses. The GO enrichment analysis revealed that these genes were significantly enriched in biological processes, cellular components, and molecular function, including neuronal cell body organization, regulation of membrane potential, neuronal cell body, and passive transmembrane transporter protein activity (Figure 7A). In the KEGG pathway analysis, the differentially expressed genes were primarily enriched in Pathways of neurodegeneration-multiple diseases and the signaling pathways of Alzheimer’s disease (Figure 7B).

### 3.6 Analysis of immune cell infiltration levels in AD and their correlation with critical genes

**FIGURE 8:**
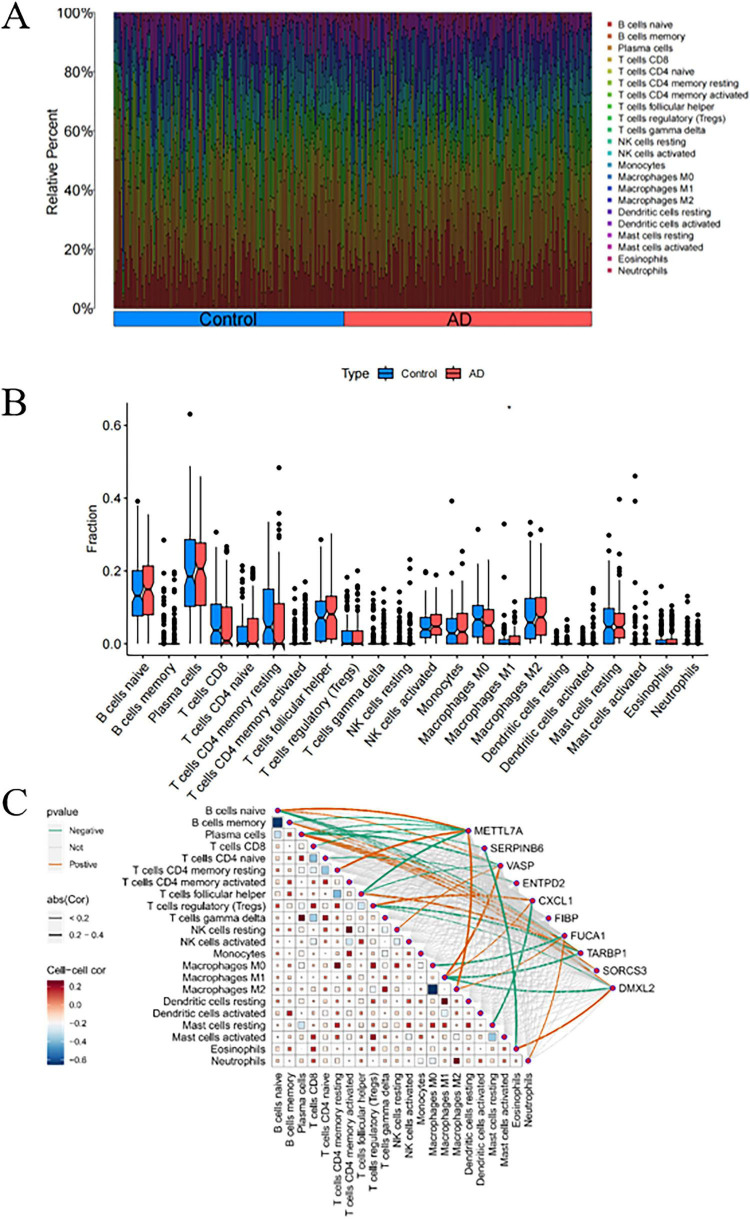
Analysis of immune cell infiltration in Alzheimer’s disease (AD). **(A)** Stacked bar plot depicting the proportional distribution of immune cell subsets between AD and control groups. **(B)** Box-and-whisker plots illustrating intergroup comparisons of 22 immune cell subsets between AD and control groups. **(C)** Heatmap displaying the correlation matrix between the 22 immune cell subsets and their co-expressed genes. *P* < 0.05 indicates statistical significance.

The CIBERSORT algorithm was employed to characterize immune cell profiles and investigate the association between Alzheimer’s disease co-expressed genes and immune cell infiltration. Figure 8A illustrates the distribution of 22 immune cell types across individual samples, depicting their proportional composition in each sample. We identified significant differences in specific immune cell subsets, specifically naive CD4^+^ T cells, between AD and healthy controls. Notably, the proportion of naive CD4^+^ T cell phenotypes was significantly elevated in AD samples relative to healthy controls (Figure 8B). Correlation analyses with 22 immune cell types (Figure 8C) revealed distinct associations for co-expressed genes: METTL7A exhibited positive correlations with naive B cells, resting memory CD4^+^ T cells, and M2 macrophages, while negatively correlating with memory B cells, plasma cells, CD8^+^ T cells, and follicular helper T cells. SERPINB6 showed negative associations with plasma cells and eosinophils. VASP was positively linked to naive B cells, resting natural killer (NK) cells, and M1 macrophages, but negatively associated with plasma cells and follicular helper T cells. ENTPD2 displayed a negative correlation with naive CD4^+^ T cell phenotypes. CXCL1 correlated positively with regulatory T cells (Tregs) and M1 macrophages, and negatively with resting mast cells. FUCA1 was positively associated with M2 macrophages and neutrophils, but negatively correlated with M0 macrophages. TARBP1 showed positive associations with memory B cells and plasma cells, while negatively correlating with Tregs and M1 macrophages. SORCS3 and DMXL2 both demonstrated positive correlations with plasma cells. Additionally, DMXL2 was positively associated with eosinophils and negatively correlated with Tregs and M1 macrophages.

### 3.7 GSEA enrichment analysis

**FIGURE 9:**
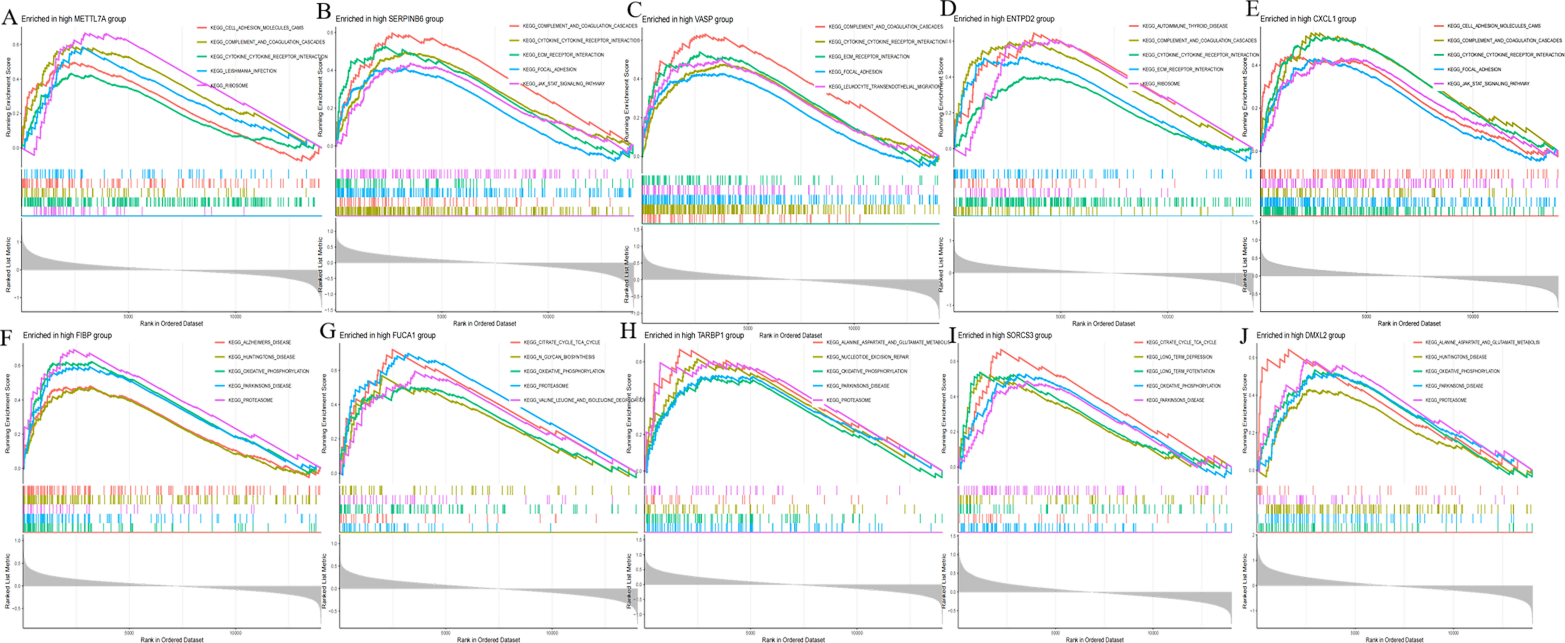
Gene set enrichment analysis (GSEA) of disease critical genes in Alzheimer’s disease (AD). **(A)** GSEA enrichment results of METTL7A high expression group. **(B)** GSEA enrichment results of SERPINB6 high expression group. **(C)** GSEA enrichment results of VASP high expression group. **(D)** GSEA enrichment results of ENTPD2 high expression group. **(E)** GSEA enrichment results of CXCL1 high expression group. **(F)** GSEA enrichment results of FIBP high expression group. **(G)** GSEA enrichment results of FUCA1 high expression group. **(H)** GSEA enrichment results of TARBP1 high expression group. **(I)** GSEA enrichment results of SORCS3 high expression group. **(J)** GSEA enrichment results of DMXL2 high expression group.

To further explore the potential regulatory mechanisms of co-expression in AD, we performed single-gene GSEA enrichment analysis for each of the ten co-expression genes in the merged dataset of GSE5281, GSE29378, GSE37263, and GSE138260. We found that the expression of the ten co-expression genes was closely associated with multiple biological pathways. Examples: Cell adhesion molecules signaling pathway, Alanine, aspartate and glutamate metabolism signaling pathway, Alzheimer disease signaling pathway, Citrate cycle (TCA cycle) signaling pathway and so on. This again demonstrates that AD progression is a complex biological process and that the 10 co-expression genes may influence AD development by regulating different pathways. Among them, we noticed that several immune-related signaling pathways were significantly enriched (Figure 9). Therefore, we hypothesized that the expression of the co-expression gene may be closely associated with the immune response in AD.

### 3.8 AD-related DEGs identification and verification via machine learning

**FIGURE 10:**
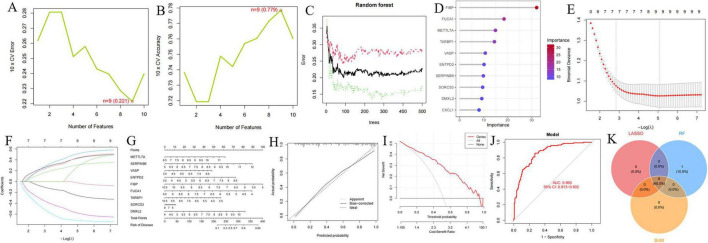
Identification and validation of diagnostic biomarkers based on critical genes using machine learning. **(A,B)** Number of genes associated with the lowest error rate and highest accuracy in the support vector machine (SVM) model. **(C,D)** Random forest analysis identifying critical genes and extracting potential diagnostic biomarkers. **(E,F)** Biomarker screening through least absolute shrinkage and selection operator (LASSO) regression analysis. **(G–J)** Visualization of the diagnostic nomogram **(G)** and evaluation of diagnostic performance **(H–J)**. **(K)** Venn diagram illustrating nine candidate diagnostic genes identified by the SVM, LASSO, and random forest algorithms.

The forest plot depicting the 10 AD-related DEGs is presented in Figure 3. Using the support vector machine (SVM) algorithm, we established that the model attained optimal accuracy with nine genes included (Figures 10A, B). We subsequently deployed the random forest (RF) algorithm to pinpoint potential diagnostic biomarkers (Figures 10C, D). Lastly, implementation of the least absolute shrinkage and selection operator (LASSO) regression algorithm generated nine candidate biomarkers, as depicted in Figures 10E, F. The nomogram indicated the importance of each gene in the diagnostic model (Figure 10G). The accuracy of the diagnostic model was evaluated using the calibration analysis, which showed high accuracy in diagnosing diseases, as demonstrated in Figures 10H, I. Furthermore, the area under the receiver operating characteristic curve (AUC) for the merged dataset (GSE5281, GSE29378, GSE37263, and GSE138260) was 0.860, indicative of robust diagnostic performance of the model for AD (Figure 10J). Finally, the intersection of genes identified by the SVM, RF, and LASSO regression analyses was visualized using a Venn diagram (Figure 10K). Nine common critical genes—METTL7A, SERPINB6, VASP, ENTPD2, FIBP, FUCA1, TARBP1, SORCS3, and DMXL2—were selected for final validation.

**FIGURE 11:**
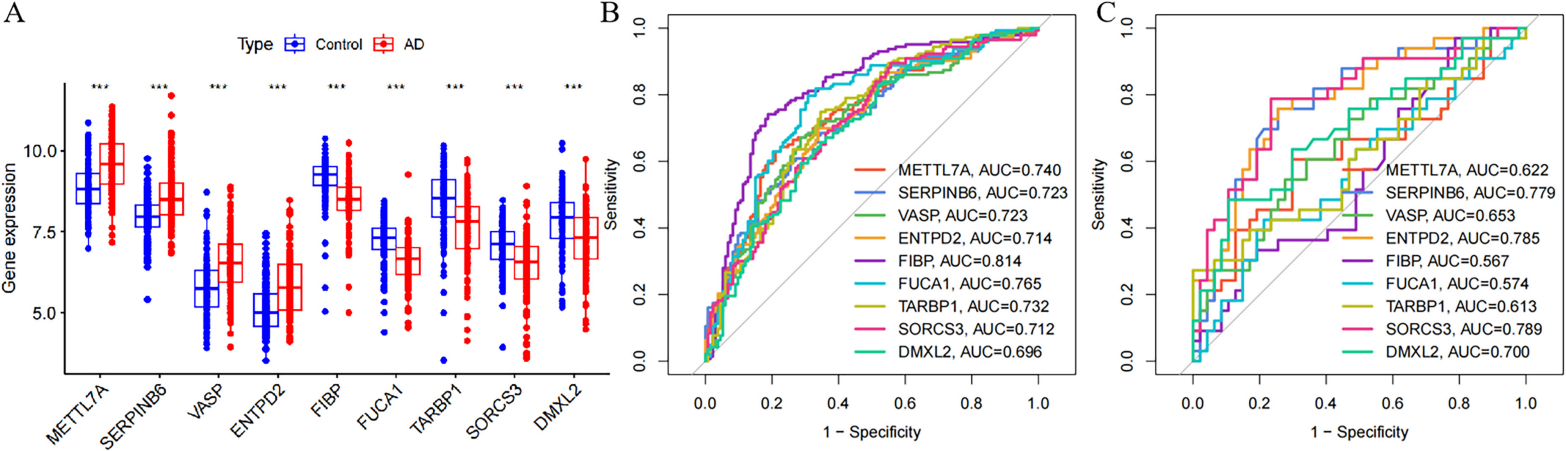
Expression of nine candidate diagnostic genes and validation of diagnostic specificity and sensitivity. **(A)** Expression of candidate diagnostic genes in the merged Alzheimer’s disease AD dataset. **(B)** Receiver operating characteristic (ROC) curves of individual candidate genes in the merged dataset. **(C)** ROC curves of individual candidate genes in the GSE36980 test dataset. This level of significance is much more stringent than *P < 0.05, **P < 0.01, and ***P < 0.0001.

The specific expression levels of these nine common critical genes were compared between AD and control groups using the Wilcoxon rank sum test, with analyses performed on the merged dataset (GSE5281, GSE29378, GSE37263, and GSE138260) (Figure 11A). Nine critical genes exhibited statistically significant differences in the merged datasets. Receiver operating characteristic curves were then constructed to assess the diagnostic specificity and sensitivity of each gene in these datasets. In the merged dataset (Figure 11B), METTL7A (AUC = 0.740), SERPINB6 (AUC = 0.723), VASP (AUC = 0.723), ENTPD2 (AUC = 0.714), FIBP (AUC = 0.814), FUCA1 (AUC = 0.765), TARBP1 (AUC = 0.732), SORCS3 (AUC = 0.712), and DMXL2 (AUC = 0.696) all showed significant diagnostic value. In the GSE36980 dataset (Figure 11C), METTL7A (AUC = 0.622), SERPINB6 (AUC = 0.779), VASP (AUC = 0.653), ENTPD2 (AUC = 0.785), FIBP (AUC = 0.567), FUCA1 (AUC = 0.574), TARBP1 (AUC = 0.613), SORCS3 (AUC = 0.789), and DMXL2 (AUC = 0.700) exhibited diagnostic value.

**FIGURE 12:**
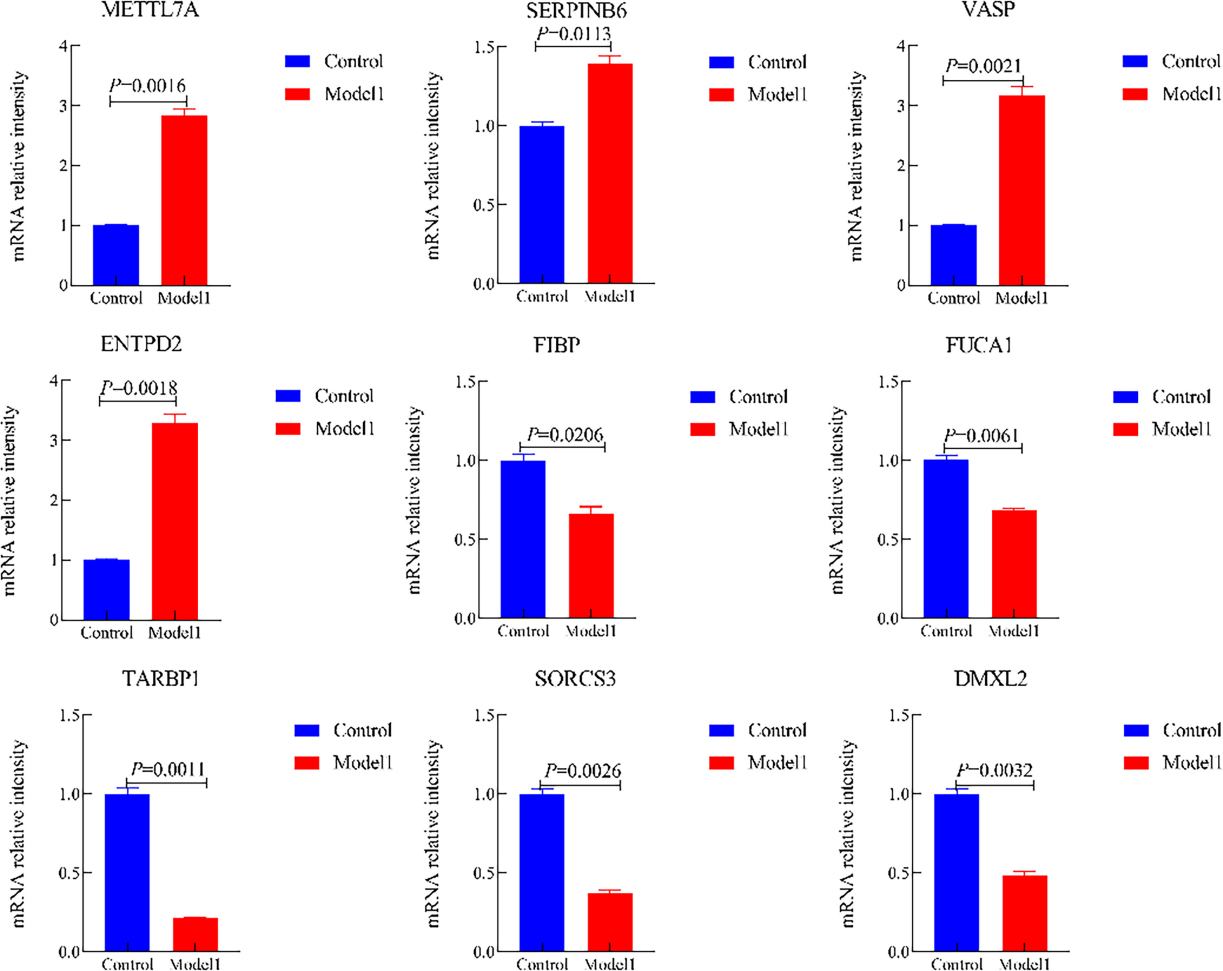
The mRNA expression of METTL7A, SERPINB6, VASP, ENTPD2, FIBP, FUCA1, TARBP1, SORCS3, and DMXL2 in the Alzheimer’s disease (AD)-associated tau pathology. (Control, the normal cell group; Model 1, the OA-induced cellular AD-like model).

**FIGURE 13:**
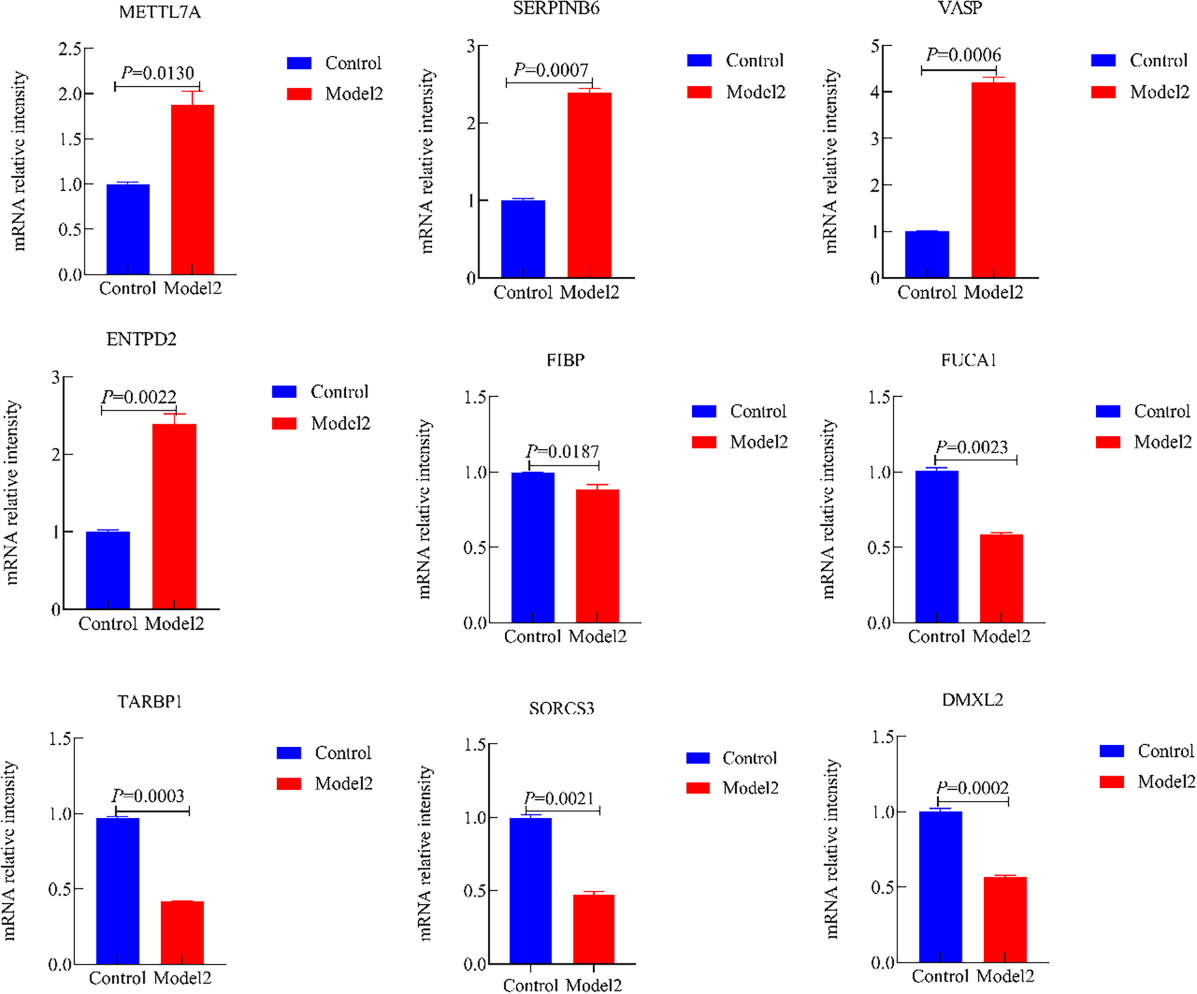
The mRNA expression of METTL7A, SERPINB6, VASP, ENTPD2, FIBP, FUCA1, TARBP1, SORCS3, and DMXL2 in the Alzheimer’s disease (AD)-associated Aβ pathology. (Control, the normal cell group; Model 2, the Aβ_1–42_-induced cellular AD-like model).

Moreover, we validated the mRNA expression of METTL7A, SERPINB6, VASP, ENTPD2, FIBP, FUCA1, TARBP1, SORCS3, and DMXL2 in AD-associated tau and Aβ pathology model. The results revealed significantly increased mRNA levels of METTL7A, SERPINB6, VASP, and ENTPD2, whereas FIBP, FUCA1, TARBP1, SORCS3, and DMXL2 exhibited reduced mRNA expression in the AD-associated tau and Aβ pathology model (Figures 12, 13). Collectively, these findings indicate that all nine candidate genes could serve as potential diagnostic markers for AD, and may be involved in AD-associated tau and Aβ pathogenesis.

## 4 Discussion

Alzheimer’s disease (AD) is a progressive, irreversible, and currently incurable neurodegenerative disorder. Most patients develop obvious clinical symptoms at the middle-late stage, with an average disease course of 5–10 years after diagnosis, and only about 10% of patients survive for more than 10 years. Consequently, AD ranks among the leading causes of death in the elderly population globally (De Luca et al., 2019; Zhang et al., 2020). Aging is the most significant risk factor for AD, with the prevalence increasing exponentially after the age of 65, accompanied by cumulative damage to neuronal structure and function (Mann, 1985). AD is characterized by typical pathological features, primarily including extracellular β-amyloid (Aβ) plaque deposition, intracellular neurofibrillary tangles (NFTs) formed by hyperphosphorylated tau protein, and extensive loss of neurons and synapses in the hippocampus and cerebral cortex (Leake, 2023).

In this study, we employed MR analysis combined with eQTL data to investigate the causal relationship between DEGs and AD-associated tau and Aβ pathology, based on six independent AD datasets from the GEO database. The MR analysis identified 10 genes significantly co-expressed with AD, namely METTL7A, SERPINB6, VASP, ENTPD2, CXCL1, FIBP, FUCA1, TARBP1, SORCS3, and DMXL2. In this study, we employed MR analysis combined with eQTL data to investigate the causal relationship between DEGs and AD, based on six independent AD datasets from the GEO database. The MR analysis identified 10 genes significantly co-expressed with AD, namely METTL7A, SERPINB6, VASP, ENTPD2, CXCL1, FIBP, FUCA1, TARBP1, SORCS3, and DMXL2. By integrating these MR-identified genes with DEGs from AD transcriptomic data, we further filtered out nine core genes (excluding CXCL1) that showed may be associated with AD-associated tau and Aβ pathology. Additionally, we conducted GO/KEGG enrichment analysis and immune cell correlation analysis for these critical genes—uncovering the unique presence of naive CD4^+^ T cells in AD and emphasizing immune processes in AD progression—and validated the genes’ expression and diagnostic value via qRT-PCR and nomogram construction. This finding has the potential to offer new insights into investigating the mechanisms underlying AD-associated tau and Aβ pathology and advancing the development of targeted therapeutic strategies against AD.

The pathological mechanisms underpinning AD—a leading cause of age-related neurodegeneration—remain only partially elucidated, leaving a critical gap in our capacity to unravel the disease’s progressive trajectory (Dong et al., 2022; Metaxas and Kempf, 2016). The genes under investigation are involved in multiple aspects of amyloid-beta (Aβ) metabolism, tau protein regulation, and other associated pathological processes—including neuroinflammation, synaptic impairment, and neuronal survival. Collectively, these regulatory roles drive the progression of AD.

Neuroinflammation acts as a pivotal amplifier in AD pathogenesis, underpinning progressive neuronal dysfunction. Brain-resident microglia (innate immune cells for homeostasis) are overactivated by stimuli like amyloid-β (Aβ) deposition, secreting pro-inflammatory mediators. This exacerbates neuronal damage and Aβ aggregation, forming a deleterious feedforward loop in AD (Heneka et al., 2025). SERPINB6 (a serine protease inhibitor) suppresses pro-inflammatory proteases, reducing cytokine release and protecting synapses (Strik et al., 2004). Clinically, SERPINB6 expression in AD patients’ frontal cortex is significantly higher than in healthy controls (Zattoni et al., 2022). FIBP modulates fibroblast growth factor 2 (FGF2)—an anti-inflammatory/neuroprotective factor that inhibits microglial activation—via direct binding. Hippocampal FIBP mRNA levels are reduced in AD patients vs. controls (Berger et al., 2020). In AD mouse models, FIBP overexpression restores FGF2 activity, reduces cerebral Aβ deposition, and improves spatial memory (Li Y. et al., 2025).

Impaired metabolism and clearance of amyloid-β (Aβ) constitute the initiating event in the pathogenesis of AD. SORCS3 modulates the intracellular trafficking of the amyloid precursor protein (APP), thereby constraining Aβ production (Eggert et al., 2018). This reduction in SORCS3 activity disrupts the normal trafficking of APP, leading to increased cleavage by β-secretase and a consequent elevation in Aβ production (Haass et al., 2012). Genome-wide association studies (GWAS) have identified a significant association between the rs10884402 polymorphism in the SORCS3 gene and heightened AD risk (Kamran et al., 2023; Ruganzu et al., 2021). FUCA1, by contrast, functions as a key glycosidase localized to lysosomes, where it mediates the degradation of fucose residues on glycoproteins and glycolipids. In AD, reduced FUCA1 activity has been observed, a deficit closely associated with abnormal lysosomal acidification—a hallmark of lysosomal dysfunction in the disease. This impairment in FUCA1 activity drives the accumulation of glycosylation waste products within lysosomes, which not only impedes the lysosomal degradation of Aβ but also compromises the phagocytic capacity of microglia toward Aβ (Huang et al., 2022; Quick et al., 2023). Mechanistic insights from cell-based experiments further demonstrate that supplementation of FUCA1 in microglia restores lysosomal function and enhances the efficiency of Aβ phagocytosis (Rao et al., 2025).

Hyperphosphorylation of tau protein is tightly linked to synaptic damage in AD, with the VASP and TARBP1 genes emerging as key regulators of this pathological process—each contributing through distinct molecular mechanisms. VASP (vasodilator-stimulated phosphoprotein), an actin cytoskeleton-binding protein, interacts with microtubule-associated proteins to modulate microtubule dynamics and maintain structural integrity. VASP participates in the release of neurotransmitters at the presynaptic membrane, supporting normal synaptic function (Venkatramani and Panda, 2019). In the AD brain, VASP expression is significantly upregulated—an effect potentially driven by heightened oxidative stress, a well-documented contributor to AD pathogenesis (Ionescu-Tucker and Cotman, 2021; Sinclair et al., 2015). Overexpression of VASP in tau transgenic mice reduces the formation of neurofibrillary tangles (NFTs)—the pathological aggregates of hyperphosphorylated tau—and restores neuronal microtubule integrity (Shim et al., 2007). TARBP1 (TAR RNA-binding protein 1), by contrast, functions as a core component of the RNA-induced silencing complex (RISC), where it regulates the maturation and functional activity of microRNAs. In AD, reduced TARBP1 expression disrupts miR-124 maturation, leading to a marked upregulation of GSK-3β. This increase in GSK-3β activity exacerbates tau hyperphosphorylation and, concurrently, impairs miRNA-mediated regulation of synaptic genes—disrupting synaptic architecture and function (Ghafouri-Fard et al., 2021). Mechanistic validation from cell-based experiments further confirms TARBP1’s role: overexpression of TARBP1 restores miR-124 activity, lowers GSK-3β expression, and reduces tau phosphorylation (Shi et al., 2024).

Neuronal survival deficits and metabolic abnormalities represent additional critical hallmarks of AD pathology, with the ENTPD2, DMXL2, and METTL7A genes emerging as key mediators of these processes—each governing distinct molecular pathways that collectively contribute to AD progression. ENTPD2 (ectonucleoside triphosphate diphosphohydrolase 2), an exonucleotidase localized to the extracellular space, plays a pivotal role in regulating extracellular adenosine triphosphate (ATP) levels—a key modulator of neuroinflammation and neuronal survival. In the AD brain, ENTPD2 activity is increased, disrupting this protective cascade. The resultant elevation in extracellular ATP levels—driven in part by widespread neuronal death in AD—activates P2X7 receptors on microglia, triggering excessive microglial activation and exacerbating neuroinflammation (John and Reddy, 2021). DMXL2 (DMX-like 2), a Golgi apparatus-associated protein, regulates two critical processes for neuronal health: the trafficking of neurotransmitter synthesis enzymes and the maintenance of neuronal calcium homeostasis via modulation of calmodulin signaling. Reduced DMXL2 impairs neurotransmitter synthesis, leading to deficiencies in dopamine and acetylcholine that drive synaptic dysfunction. Concurrently, it disrupts calcium homeostasis, increasing the risk of neuronal apoptosis (Costain et al., 2019). METTL7A (methyltransferase-like 7A), a protein with putative methyltransferase activity, contributes to neuronal metabolic homeostasis through two distinct mechanisms: regulation of lipid metabolism and modulation of RNA methylation (Lee et al., 2021). In AD, single-cell sequencing studies reveal elevated METTL7A expression in microglia from AD patients—suggesting a potential compensatory response to AD-related metabolic stress (Mathys et al., 2019). Collectively, these genes demonstrate significant value as potential biomarkers and therapeutic targets in AD research. Future studies should focus on elucidating the specific molecular regulatory mechanisms of these genes, as well as systematically investigating their synergistic or antagonistic interactions in the pathological progression of AD. This will establish a robust theoretical and experimental foundation for the development of innovative diagnostic technologies and precision treatment strategies.

It should be noted that the *in vitro* model used in this study has certain limitations. Firstly, the SK-N-SH cells used are a neuroblastoma cell line whose cellular phenotype and physiological functions differ significantly from those of primary neurons *in vivo*, making it difficult to fully mimic the pathological response characteristics of normal neurons. Secondly, the model fails to encompass the complex pathological components of AD progression, such as the neuroinflammatory microenvironment and synaptic damage. This limits the clinical translational value of the findings.

Therefore, subsequent studies should optimize experimental systems further. This could be achieved by integrating primary neuronal cultures and brain organoid models derived from AD patients, which can mimic the brain’s three-dimensional microenvironment and cellular heterogeneity, as well as AD animal models, such as APP/PS1 transgenic mice. This would enable researchers to validate the pathological functions and regulatory mechanisms of core genes across multiple levels, from cells and organoids to whole animals. This multidimensional approach will provide more robust experimental evidence for their eventual application in the clinical diagnosis and treatment of AD.

## 5 Conclusion

In summary, this study has clarified the regulatory roles of METTL7A, SERPINB6, VASP, ENTPD2, FIBP, FUCA1, TARBP1, SORCS3, and DMXL2 in AD progression. Combined with functional enrichment analysis, it is inferred that these genes participate in the AD pathogenesis by regulating key pathological processes such as Aβ metabolism and tau phosphorylation. This fills a research gap regarding the roles of these genes in the molecular regulatory network of AD and deepens the systematic understanding of AD pathological mechanisms. On the other hand, the identified core genes provide potential biomarkers for developing highly specific and sensitive AD diagnostic reagents, while also offering key targets for AD-specific therapeutic drug development. This holds promise for overcoming the current challenges of delayed AD diagnosis and limited treatment options, laying the foundation for precision medicine in AD.

## Data Availability

The datasets presented in this study can be found in online repositories. The names of the repository/repositories and accession number(s) can be found in this article/Supplementary material.

## Footnotes

1
https://www.ncbi.nlm.nih.gov/geo/

2
https://gwas.mrcieu.ac.uk/
