# Supplementary material for: Deciphering Alzheimer’s disease transcriptomics: exploration and validation of core genes in tau and Aβ pathological models toward novel therapeutic targets
Source: Front Aging Neurosci. 2025 Oct 10;17:1621153. doi: 10.3389/fnagi.2025.1621153 (PMC12549628; doi:10.3389/fnagi.2025.1621153)
Supplement: Supplementary file 1 [file Table_1.docx]

Supplementary Material

# Supplementary Table S1 Differential genes

| id | logFC | AveExpr | t | P.Value | adj.P.Val | B |
| --- | --- | --- | --- | --- | --- | --- |
| DTNA | 1.08416592040486 | 10.0473937113768 | 12.2899666224618 | 4.65653226372516e-28 | 6.52985519342179e-24 | 52.8405199128684 |
| SORBS1 | 0.617389775614912 | 9.27314478789855 | 11.2541660871836 | 1.71849748578295e-24 | 1.20492451215672e-20 | 44.8245966994341 |
| ITPKB | 1.08086644643146 | 8.14025622913043 | 11.0161857894893 | 1.094615915979e-23 | 5.11659966325785e-20 | 43.0177415598335 |
| SASH1 | 0.892867305840472 | 9.61650965775362 | 10.75222863 | 8.38383359328714e-23 | 2.93916246196664e-19 | 41.0311449695018 |
| PFKFB3 | 0.889304499423197 | 10.9450322677174 | 10.5766744258865 | 3.21185034034185e-22 | 9.00795546452277e-19 | 39.7206775489105 |
| GLIS3 | 0.913383243825653 | 7.23804445710145 | 10.5387138007231 | 4.28912959688022e-22 | 1.00244107228419e-18 | 39.4384903333282 |
| PALLD | 0.81844835871234 | 8.43294015778985 | 10.4126145101999 | 1.11758908674763e-21 | 2.23885025192315e-18 | 38.5042022998181 |
| GFAP | 1.36733688600347 | 11.746344398913 | 10.3352211570833 | 2.00676346422092e-21 | 3.51760550734625e-18 | 37.9331850558412 |
| TGFBR3 | 0.895940125650666 | 8.65189849039855 | 10.0292174601248 | 1.99286941296326e-20 | 3.10511197533153e-17 | 35.6940758707886 |
| NAV2 | 0.608676128864818 | 7.87092637913043 | 9.86572208219396 | 6.70822279686987e-20 | 8.91393521991679e-17 | 34.510444026119 |
| YAP1 | 1.04178633719964 | 7.96296652753623 | 9.86011288027741 | 6.99231886323074e-20 | 8.91393521991679e-17 | 34.4699986620162 |
| ITGB5 | 0.836294920078872 | 8.0360178592029 | 9.70143999157637 | 2.25007277706889e-19 | 2.62939754606976e-16 | 33.3304619146328 |
| AFF1 | 0.734102268044582 | 7.80708061311594 | 9.67386549403269 | 2.75426231060002e-19 | 2.97100156781108e-16 | 33.1333459428617 |
| TRIM8 | 0.58614504820601 | 9.61503228547102 | 9.6230437845294 | 3.99510690315926e-19 | 3.90178918916999e-16 | 32.7707696406659 |
| CA10 | -0.956981498 | 8.08680952018116 | -9.61706162 | 4.17363173625828e-19 | 3.90178918916999e-16 | 32.7281531598598 |
| BBX | 0.657905351498501 | 8.73326762952899 | 9.53681058232811 | 7.49296856854577e-19 | 6.56711863979483e-16 | 32.1577251260579 |
| KLF15 | 0.917973114191067 | 8.03397153188406 | 9.5040564913707 | 9.50781761061961e-19 | 7.40711813076216e-16 | 31.9255947995401 |
| ZFP36L1 | 1.06713885724223 | 8.33826421873188 | 9.49112206751398 | 1.04442066319155e-18 | 7.70837418943951e-16 | 31.8340384139928 |
| TNPO1 | 0.684755299200802 | 9.21935350833333 | 9.47433308814401 | 1.17974007926692e-18 | 8.05291884575164e-16 | 31.7152914258587 |
| IDH3G | -0.786156419 | 8.37191162086956 | -9.471302721 | 1.20595661242804e-18 | 8.05291884575164e-16 | 31.6938692313937 |
| PLSCR4 | 1.04405380428677 | 9.05943113003623 | 9.45157725214168 | 1.39132313627369e-18 | 8.86842015453e-16 | 31.5545113075515 |
| CMBL | 0.881366749467377 | 9.17850523028986 | 9.41541883706657 | 1.80754399890595e-18 | 1.07000052632661e-15 | 31.2994403850462 |
| ACACB | 1.00417327413429 | 8.21688899376812 | 9.41361471662453 | 1.83127808827202e-18 | 1.07000052632661e-15 | 31.2867267035409 |
| HVCN1 | 1.07103832155476 | 6.83251298568841 | 9.40518015002769 | 1.9464081645554e-18 | 1.09177926766242e-15 | 31.2273046192657 |
| GAS2L1 | 0.622122626507698 | 8.77670237663044 | 9.38348396195422 | 2.27663195978202e-18 | 1.22789269123166e-15 | 31.0745788247572 |
| MALAT1 | 0.80775773806457 | 11.4474290820652 | 9.35848883018491 | 2.72648196155125e-18 | 1.36548059095833e-15 | 30.8988553426449 |
| SST | -1.80594683 | 7.45257224847826 | -9.276723034 | 4.90951795966814e-18 | 2.29487234494755e-15 | 30.3257109896422 |
| RAP1GDS1 | -0.677164485 | 8.47001158242754 | -9.244935376 | 6.16646461239955e-18 | 2.78943010515093e-15 | 30.1035999670212 |
| NFIA | 0.837463793704712 | 9.04083962141304 | 9.23796999688235 | 6.48194894718274e-18 | 2.84051156519823e-15 | 30.0549838553728 |
| CTDSP2 | 0.603324870083602 | 9.35833327521739 | 9.16685388492452 | 1.07762774896411e-17 | 4.57926482537081e-15 | 29.5597213653057 |
| KCNE4 | 0.907831422872391 | 5.53018531278985 | 9.15776032452228 | 1.14983148913576e-17 | 4.74237852122083e-15 | 29.4965385677993 |
| ERBB2IP | 0.824402279000466 | 8.48709749068841 | 9.09762471599135 | 1.7641147947015e-17 | 7.0680519331712e-15 | 29.0795527686672 |
| LPP | 0.69733481601504 | 8.6104357690942 | 9.06721421496709 | 2.18922062932567e-17 | 8.49135779457638e-15 | 28.8692437260748 |
| TNS1 | 0.899847448275941 | 8.07500533 | 9.06395200691669 | 2.24046379804126e-17 | 8.49135779457638e-15 | 28.8467058511953 |
| ATP5B | -0.767369585 | 11.4197826655435 | -9.019842427 | 3.06210308125799e-17 | 1.1010223463713e-14 | 28.5423924289582 |
| ID4 | 0.903297324438197 | 7.95609894771739 | 9.01610864078186 | 3.14404688412284e-17 | 1.10222423640137e-14 | 28.5166697913453 |
| ADAM33 | 0.891901977221729 | 7.38995550485507 | 9.01041451835946 | 3.27321877402673e-17 | 1.11952065532139e-14 | 28.4774532168912 |
| DDR1 | 0.729446301188288 | 9.23233336050725 | 9.00150159216927 | 3.48605261275429e-17 | 1.14206140019216e-14 | 28.4160950927149 |
| BCL6 | 0.81294578616173 | 9.3377621367029 | 9.00085538125187 | 3.50200671812471e-17 | 1.14206140019216e-14 | 28.4116477476178 |
| PTTG1IP | 0.665191311083122 | 10.5443134172826 | 8.9846435602236 | 3.92682733744827e-17 | 1.25149772165993e-14 | 28.3001317845655 |
| CREBBP | 0.614683175364642 | 7.88625432673913 | 8.93904796673289 | 5.41548515610771e-17 | 1.65089887704562e-14 | 27.9870826860599 |
| SAMHD1 | 0.595977301137808 | 8.06400927253623 | 8.89497643054361 | 7.38255752622374e-17 | 2.14852398849695e-14 | 27.6853283458961 |
| MXI1 | 0.735061069532572 | 9.44668857217391 | 8.89258610769449 | 7.50750020939532e-17 | 2.14852398849695e-14 | 27.6689854849485 |
| RFX4 | 0.827345708801198 | 7.40889014844203 | 8.88002450934559 | 8.19937205673344e-17 | 2.29959588703146e-14 | 27.5831405416629 |
| CEBPB | 0.816239071909147 | 9.69708199536232 | 8.84608387931537 | 1.04010994045064e-16 | 2.85989444998809e-14 | 27.3515292118241 |
| CASP6 | 0.64050877707556 | 6.18096143623188 | 8.79611301392277 | 1.47494879628845e-16 | 3.97753980199094e-14 | 27.0114260150867 |
| ATP5C1 | -0.799594836 | 9.7476593126087 | -8.792064935 | 1.51720890789239e-16 | 4.00018129421579e-14 | 26.9839218028669 |
| TBL1X | 0.759149263145287 | 7.79773190463768 | 8.74478529104742 | 2.10914828437491e-16 | 5.2815332842481e-14 | 26.6632120216369 |
| FAM63A | 0.926264746848411 | 8.38556826894927 | 8.72525468832201 | 2.41591136359249e-16 | 5.84109052614784e-14 | 26.5310153318334 |
| MYO10 | 0.805055373756771 | 8.42156461463768 | 8.70374433605255 | 2.80510523726614e-16 | 6.66711707494628e-14 | 26.3856117613197 |
| GPHN | -0.64186084 | 7.38842473923913 | -8.670119574 | 3.54139065983814e-16 | 8.27682020381836e-14 | 26.1587265330169 |
| ME3 | -0.615762046 | 7.58006899536232 | -8.661975751 | 3.74677530704382e-16 | 8.40698215867108e-14 | 26.1038507515868 |
| LIFR | 0.849551228811185 | 8.41112509521739 | 8.66081719190559 | 3.77693700346772e-16 | 8.40698215867108e-14 | 26.0960463818859 |
| PCYOX1L | -0.782882526 | 7.69130220833333 | -8.627585982 | 4.75233999910859e-16 | 1.04128224699218e-13 | 25.8724459138204 |
| TNFRSF1A | 0.970601725570742 | 7.51827029271739 | 8.61452716106677 | 5.20061660809673e-16 | 1.12197302608216e-13 | 25.7847126859235 |
| MSI2 | 0.634913439067248 | 7.70572522992754 | 8.60210892208231 | 5.66566978039249e-16 | 1.20378314137036e-13 | 25.7013538538742 |
| BAG3 | 1.04221730550713 | 9.21276744028986 | 8.53760274535855 | 8.83048303445497e-16 | 1.79463570423423e-13 | 25.2694650144371 |
| SAMD4A | 0.707968145650664 | 7.5407503909058 | 8.52983196301373 | 9.31423760222559e-16 | 1.86590791280013e-13 | 25.2175642168705 |
| COL27A1 | 0.65237889086598 | 6.83284203021739 | 8.5262675143931 | 9.5448282599801e-16 | 1.88517079844649e-13 | 25.1937665614383 |
| CXCR4 | 0.913636115680637 | 5.92597187351449 | 8.5206418052948 | 9.92031989766769e-16 | 1.93212008229158e-13 | 25.1562188808994 |
| SEMA4C | 0.639561806079183 | 8.81878031471014 | 8.51364465509056 | 1.04078187896404e-15 | 1.99929921763188e-13 | 25.1095378942416 |
| NME1 | -0.84844982 | 9.36286207456522 | -8.497856838 | 1.15966506300509e-15 | 2.19756529439465e-13 | 25.004292630203 |
| NOTCH2 | 0.823352895222144 | 8.12027042025362 | 8.47339078635573 | 1.37097051113492e-15 | 2.563349263686e-13 | 24.8414214684975 |
| MKNK2 | 0.901543216967767 | 9.731381968 | 8.45778804572215 | 1.52519819396587e-15 | 2.77764341220564e-13 | 24.7376970683583 |
| DDIT4 | 1.01240856567065 | 9.61978667163043 | 8.45174521505379 | 1.58944034627405e-15 | 2.85752845843603e-13 | 24.6975554444577 |
| DNAJC1 | 0.642668937047692 | 8.26154871894927 | 8.4444368201375 | 1.67072536672684e-15 | 2.96564326805196e-13 | 24.6490293595723 |
| QKI | 0.724715338775441 | 9.23476134496377 | 8.40341848969998 | 2.20942053610954e-15 | 3.86156025115614e-13 | 24.3771354248433 |
| GJA1 | 1.06881508483043 | 11.8164938944565 | 8.40202124463466 | 2.2305239987424e-15 | 3.86156025115614e-13 | 24.3678874006007 |
| AP3M2 | -0.706705376 | 8.73257858311594 | -8.380035007 | 2.59011533569091e-15 | 4.42941309175532e-13 | 24.2224855901597 |
| KANK1 | 0.638826881302906 | 7.93726163876812 | 8.3698343658077 | 2.77589103695869e-15 | 4.68991807364719e-13 | 24.1551022021707 |
| MAFF | 1.11027095316631 | 7.88574733952899 | 8.33179025671968 | 3.59264958875115e-15 | 5.72496877080198e-13 | 23.9042205899205 |
| C9orf64 | 0.742395362156795 | 8.68362989557971 | 8.32820175232102 | 3.6809952505933e-15 | 5.79984229203032e-13 | 23.8805913430857 |
| SRGAP1 | 0.653296103682104 | 6.94154279452899 | 8.31762614184297 | 3.95405657728158e-15 | 6.0269277590456e-13 | 23.8109893796606 |
| GRAMD3 | 0.859280979897479 | 8.75412913083333 | 8.2940974152784 | 4.63556686542735e-15 | 6.91537810147741e-13 | 23.656327868771 |
| PLOD2 | 0.73328307481203 | 8.14733447626812 | 8.27927999961473 | 5.12313546928384e-15 | 7.56228723008077e-13 | 23.5590631478997 |
| CD200 | -0.846188724 | 8.29334384753623 | -8.277403121 | 5.18840842622338e-15 | 7.5788595167636e-13 | 23.5467503587014 |
| CD44 | 0.662758101516382 | 6.58522880442029 | 8.25083330795815 | 6.20572849565595e-15 | 8.97143615408077e-13 | 23.372625795938 |
| MLLT11 | -0.865809368 | 11.926857209529 | -8.249281024 | 6.27091851500088e-15 | 8.97317248325075e-13 | 23.3624633588526 |
| TPD52L1 | 0.695568979285456 | 9.17621979416667 | 8.22881471416092 | 7.19650488818462e-15 | 1.01935947522235e-12 | 23.2285831451552 |
| RIN2 | 0.76301170827489 | 8.55291357768116 | 8.21957811723515 | 7.65732592821979e-15 | 1.07378681491426e-12 | 23.1682277944269 |
| HIF3A | 0.650159321634679 | 6.75308382902174 | 8.18798874562053 | 9.46524256925293e-15 | 1.31416927275875e-12 | 22.9621214591972 |
| TOB1 | 0.758636131042648 | 9.45248080978261 | 8.18166896053454 | 9.87468636570371e-15 | 1.35757575398297e-12 | 22.9209455357544 |
| NFKBIA | 0.743190724786272 | 9.18659329503623 | 8.16398436216168 | 1.11158824592683e-14 | 1.51337883229436e-12 | 22.8058260844494 |
| ADCYAP1 | -1.11851074 | 5.64748311931159 | -8.155617056 | 1.17557503895332e-14 | 1.58510468954254e-12 | 22.7514112364136 |
| SLC35B1 | -0.696403491 | 8.14187599 | -8.154059653 | 1.18788083581875e-14 | 1.58644313911298e-12 | 22.7412867860721 |
| SALL3 | 0.623293241738787 | 7.73798324923913 | 8.14692577728265 | 1.24589759022151e-14 | 1.64786636049535e-12 | 22.6949255473744 |
| SLC25A4 | -0.667343708 | 9.20170967496377 | -8.129186603 | 1.4025954198139e-14 | 1.79374087190169e-12 | 22.5797506468363 |
| UNG | 0.606215063906624 | 7.60903078304348 | 8.12871081765275 | 1.4070562355358e-14 | 1.79374087190169e-12 | 22.5766636376754 |
| CLDN15 | 0.656828571885486 | 6.53856805931159 | 8.10560210000926 | 1.64147195176988e-14 | 2.05521081961331e-12 | 22.4268619049687 |
| CEBPD | 0.723050838525682 | 9.97054471942029 | 8.10047418146059 | 1.69851133475702e-14 | 2.07268507845989e-12 | 22.3936557526476 |
| ACOT7 | -0.752260882 | 8.95903152931159 | -8.100370291 | 1.69968697621717e-14 | 2.07268507845989e-12 | 22.3929831339259 |
| PMP2 | 0.941927898999422 | 10.1255146540217 | 8.09906286938081 | 1.71455087428758e-14 | 2.07268507845989e-12 | 22.3845189785363 |
| ATP6V1E1 | -0.85404985 | 10.8284875508696 | -8.097652445 | 1.73072996659285e-14 | 2.07436122406253e-12 | 22.3753889303296 |
| INA | -1.026935927 | 10.2224349466667 | -8.077633272 | 1.97733019252637e-14 | 2.33223643212291e-12 | 22.2459049587491 |
| GRIN1 | -0.793278167 | 7.98871675536232 | -8.077494954 | 1.97914950739946e-14 | 2.33223643212291e-12 | 22.2450110052152 |
| SOX9 | 0.882398132706776 | 9.34464890706522 | 8.07264198577686 | 2.04404002680659e-14 | 2.3886311079924e-12 | 22.2136520585097 |
| KIF5B | 0.653987612789852 | 9.39216820018116 | 8.06558713429143 | 2.14214184202478e-14 | 2.47564720197351e-12 | 22.1680856830371 |
| SLC39A12 | 1.04406985166413 | 8.36575551594203 | 8.04729822214881 | 2.41867141681425e-14 | 2.75748205512083e-12 | 22.0500744632425 |
| CHGB | -1.301033408 | 9.57056224768116 | -8.040504289 | 2.53014987876872e-14 | 2.86131385080434e-12 | 22.0062780372008 |
| CCKBR | -0.657757786 | 6.92464194786232 | -8.03749138 | 2.58119927856321e-14 | 2.89569259866335e-12 | 21.986862936233 |
| BACE2 | 0.66549532327831 | 6.56601721445652 | 8.02939985849388 | 2.72339370739816e-14 | 3.03096428244797e-12 | 21.9347437035671 |
| SLC1A6 | -0.846806262 | 6.37046979572464 | -8.02099143 | 2.87936416060057e-14 | 3.15447840813295e-12 | 21.8806176707443 |
| ANP32B | 0.769083552922863 | 10.5791384707246 | 8.01500435194508 | 2.9957716569767e-14 | 3.25656635238638e-12 | 21.8420995886363 |
| VCAN | 0.726330032484357 | 8.70941757847826 | 8.01336576314642 | 3.02843396712375e-14 | 3.26674842469049e-12 | 21.8315607801436 |
| NEDD1 | 0.60851732056627 | 6.54728053568841 | 8.0053663624352 | 3.19301224296139e-14 | 3.3920917184127e-12 | 21.7801307507383 |
| TUBB | -0.59867555 | 9.1839560309058 | -7.964771689 | 4.17462782726244e-14 | 4.36871686729113e-12 | 21.519631043414 |
| MT1M | 0.94012680272832 | 9.03229866800725 | 7.95373473330433 | 4.48960308555201e-14 | 4.60717551391614e-12 | 21.4489488021006 |
| FIBP | -0.686534505 | 8.76817570612319 | -7.953347998 | 4.50105573277124e-14 | 4.60717551391614e-12 | 21.4464732052476 |
| ARPC1A | -1.267932047 | 8.16601470876812 | -7.937198668 | 5.00597146342104e-14 | 4.94523257815924e-12 | 21.3431642580541 |
| PTMA | 0.640157317823761 | 10.0361575498913 | 7.93633915418248 | 5.03436032256107e-14 | 4.94523257815924e-12 | 21.3376695419146 |
| FGF12 | -0.791923385 | 7.50423391637681 | -7.936081026 | 5.04291705538595e-14 | 4.94523257815924e-12 | 21.33601945 |
| MRGPRF | 0.97472408307377 | 6.00073698456522 | 7.92849716613428 | 5.3008329891781e-14 | 5.13149347947098e-12 | 21.2875543897021 |
| GPI | -0.822432699 | 9.39424561963768 | -7.928347727 | 5.3060440314005e-14 | 5.13149347947098e-12 | 21.2865996812647 |
| PSMA5 | -0.679962878 | 9.37589688554348 | -7.918557483 | 5.65869858909209e-14 | 5.43395146256292e-12 | 21.2240783477944 |
| ZFP36L2 | 0.746065797082917 | 7.24323368898551 | 7.91754965897122 | 5.69629084359088e-14 | 5.43395146256292e-12 | 21.2176450491743 |
| ADD3 | 0.588505840666709 | 10.5126077844928 | 7.9104466590215 | 5.96832745200768e-14 | 5.65499026077728e-12 | 21.1723186846626 |
| UCHL1 | -0.973471659 | 10.1561008600362 | -7.905951614 | 6.14707553825277e-14 | 5.78526444784688e-12 | 21.1436476776121 |
| NUDT2 | -1.001001068 | 6.83047070076087 | -7.902054728 | 6.30631220339979e-14 | 5.89556106855168e-12 | 21.1188002318322 |
| GLS2 | -0.738391011 | 6.9715208407971 | -7.898459532 | 6.45683307059401e-14 | 5.95685329927235e-12 | 21.0958832805681 |
| ITGB8 | 0.660409473105314 | 6.86953650387681 | 7.88698720382775 | 6.96128990193531e-14 | 6.38027243757117e-12 | 21.0227988317256 |
| MDH1 | -1.045683392 | 11.3454429476812 | -7.876444325 | 7.45912063107877e-14 | 6.74833861997533e-12 | 20.9556945154141 |
| TNS3 | 0.903853563248328 | 9.74406737992754 | 7.87331953206204 | 7.61331844558809e-14 | 6.84369003605652e-12 | 20.93581642 |
| NCALD | -0.904322334 | 8.80326545402174 | -7.869231501 | 7.81981367271302e-14 | 6.98453803391431e-12 | 20.9098182967928 |
| SEPP1 | 0.909904429536772 | 9.48528160028986 | 7.86443032245386 | 8.06940710205307e-14 | 7.16185416405634e-12 | 20.8792957582245 |
| IRAK1 | 0.59818345380409 | 8.77948492427536 | 7.86119906600316 | 8.241802194662e-14 | 7.26885485382045e-12 | 20.8587603171943 |
| SLC7A2 | 1.0960093699653 | 6.89991599568841 | 7.85067706495731 | 8.82878814034781e-14 | 7.73788100575609e-12 | 20.7919273842918 |
| COPS3 | -0.771284541 | 8.18474905514493 | -7.842433389 | 9.31736890058599e-14 | 8.11537044055387e-12 | 20.7396053740705 |
| ZIC2 | 1.00407819393185 | 8.2189101190942 | 7.80275220262917 | 1.20693020827848e-13 | 1.03833020310976e-11 | 20.4882403271828 |
| SRRM2 | 0.845280014251014 | 10.3185018750725 | 7.78590116559065 | 1.34680162080584e-13 | 1.14461812900365e-11 | 20.3817411062095 |
| PHYHD1 | 0.902331789411646 | 6.90926557050725 | 7.77924565856151 | 1.40635466497538e-13 | 1.18803081126203e-11 | 20.3397185172159 |
| NDUFA7 | -0.865935084 | 7.29978466054348 | -7.758771477 | 1.60634903890249e-13 | 1.34885224985207e-11 | 20.2105894716547 |
| PSMB3 | -0.60336535 | 9.5227808482971 | -7.748731845 | 1.71443608561129e-13 | 1.43104388265043e-11 | 20.1473499267388 |
| HIPK2 | 0.829570772463329 | 9.89409413202899 | 7.73964476690071 | 1.81844384704736e-13 | 1.50887799213877e-11 | 20.0901557818352 |
| EEF1A2 | -0.883621953 | 10.1368634415942 | -7.734124828 | 1.88464063744988e-13 | 1.54551553561168e-11 | 20.0554342712911 |
| PSMB7 | -0.60351519 | 9.02250171297101 | -7.732274694 | 1.90735620382816e-13 | 1.55504977013269e-11 | 20.0438001203771 |
| CHRM1 | -1.049861378 | 8.07623277210145 | -7.728847064 | 1.9501567769102e-13 | 1.58075424754981e-11 | 20.0222509665468 |
| DYNC1I1 | -0.910365131 | 9.64438916373188 | -7.7049448 | 2.27616532485201e-13 | 1.82392379145141e-11 | 19.872150842096 |
| ATP6V1B2 | -0.881297412 | 10.3278870692029 | -7.702700577 | 2.30940733827763e-13 | 1.84004654003791e-11 | 19.8580730689706 |
| DHCR24 | -0.904532601 | 8.51777508449275 | -7.684173825 | 2.60278678199434e-13 | 2.04738028526369e-11 | 19.7419578320664 |
| PAQR8 | 0.630938249400602 | 10.0285902440217 | 7.68354124052884 | 2.61342844656778e-13 | 2.04738028526369e-11 | 19.7379963391518 |
| IMMT | -0.606291256 | 8.69395761456522 | -7.679020479 | 2.69073993983235e-13 | 2.09623589868161e-11 | 19.709691672173 |
| AHNAK | 0.807820948451018 | 7.54607166184783 | 7.67047197997297 | 2.84315477110948e-13 | 2.19063512941034e-11 | 19.6561986230908 |
| EFHD1 | 0.776730005091224 | 10.2275275064855 | 7.66717548556305 | 2.90418014379309e-13 | 2.21190894672961e-11 | 19.6355807952218 |
| WDR70 | -0.717170116 | 6.97585735855072 | -7.657327962 | 3.09428716293046e-13 | 2.32038443239432e-11 | 19.5740239295541 |
| SF3B5 | -0.58815717 | 8.39412569101449 | -7.652979024 | 3.18210662509024e-13 | 2.37354687253406e-11 | 19.5468550430762 |
| CHST6 | 1.20981112353909 | 6.38522577634058 | 7.64037730050204 | 3.45071312438983e-13 | 2.54680790227993e-11 | 19.4681855239745 |
| TXNDC9 | -0.60980263 | 7.28587977202899 | -7.638455152 | 3.49360546796956e-13 | 2.56496489410142e-11 | 19.4561934120716 |
| WWTR1 | 0.725308552888168 | 6.48780438384058 | 7.63369539541146 | 3.60209715293148e-13 | 2.59858994653618e-11 | 19.4265061585527 |
| SCRIB | 0.686988804877755 | 8.98757862105072 | 7.63191805269601 | 3.64345635466776e-13 | 2.60674430926051e-11 | 19.4154237121625 |
| ID3 | 1.32117096994899 | 7.6947920934058 | 7.62840614238992 | 3.72656262002431e-13 | 2.65266942236553e-11 | 19.3935304723879 |
| XRCC2 | 0.730725559812296 | 8.26189160547101 | 7.6150656413045 | 4.05966234305422e-13 | 2.87518409276007e-11 | 19.3104255869574 |
| HMP19 | -0.86185114 | 10.1428392940942 | -7.612183701 | 4.1353937105093e-13 | 2.91410180916944e-11 | 19.2924849301292 |
| ATP8B1 | 0.691903684241021 | 7.31029395050725 | 7.60386810358087 | 4.36181552218235e-13 | 3.04307159540115e-11 | 19.2407434816477 |
| SPP1 | 0.848057093554864 | 9.99150818608696 | 7.58752286133329 | 4.84312703283475e-13 | 3.33358849974953e-11 | 19.1391474132188 |
| TUBA1B | -0.659555128 | 12.3845974917029 | -7.584922121 | 4.92439920177971e-13 | 3.36852926861253e-11 | 19.1229953280611 |
| CRMP1 | -0.689893334 | 9.60160004597826 | -7.575896069 | 5.21704228774155e-13 | 3.55138757286406e-11 | 19.0669664731147 |
| NRXN3 | -0.816716835 | 8.46130179873188 | -7.562537201 | 5.68193200139356e-13 | 3.8491658191083e-11 | 18.9841219858183 |
| PRDX6 | 0.618802398850098 | 10.7712083952536 | 7.55481689437834 | 5.96900018667575e-13 | 4.006775043258e-11 | 18.9362884986059 |
| CDC42EP4 | 0.623904337657612 | 8.55222889213768 | 7.5547451992455 | 5.97173204051147e-13 | 4.006775043258e-11 | 18.9358444399585 |
| CRYM | -0.71009794 | 9.4376747215942 | -7.551758803 | 6.08662781567011e-13 | 4.05093481349789e-11 | 18.9173500309037 |
| CSRP1 | 0.674759139152439 | 11.0018970551449 | 7.55153504672004 | 6.09532372279866e-13 | 4.05093481349789e-11 | 18.9159645289255 |
| CHCHD6 | -0.848577589 | 8.05534522836957 | -7.542952026 | 6.43830660749322e-13 | 4.23870298389096e-11 | 18.8628386241264 |
| METTL7A | 0.733495665194811 | 9.22498822326087 | 7.53342938103664 | 6.84115350078693e-13 | 4.44942401549072e-11 | 18.8039432276337 |
| SRGN | 0.885043329961624 | 8.60481931818841 | 7.53314483279796 | 6.85356619372457e-13 | 4.44942401549072e-11 | 18.8021841138721 |
| ATP11C | 0.6638810967522 | 6.06738338728261 | 7.5130245760871 | 7.78999012725963e-13 | 4.98808363262839e-11 | 18.6779089937177 |
| SLC26A6 | 0.678521044633261 | 7.15741584797101 | 7.50885621743023 | 7.99922904063273e-13 | 5.09878131076331e-11 | 18.6521900210014 |
| ZNF423 | 0.772887156169096 | 7.71502964978261 | 7.49979760377995 | 8.47325377140465e-13 | 5.35227196560394e-11 | 18.5963304043294 |
| EGR1 | -0.681870202 | 8.42834384195652 | -7.458729892 | 1.09940669570807e-12 | 6.70303482344099e-11 | 18.3436471994842 |
| TJP1 | 0.616287028561974 | 9.97206827992754 | 7.45154217830621 | 1.15057281666636e-12 | 6.98462450567632e-11 | 18.2995168608706 |
| RAB20 | 0.691580620749255 | 5.9436121890942 | 7.44781971397957 | 1.17798753539415e-12 | 7.12022379691042e-11 | 18.2766731797839 |
| TRIP10 | 0.722405666814241 | 6.60338685514493 | 7.42824192116317 | 1.3331298059441e-12 | 7.89366470220766e-11 | 18.1566548353462 |
| ANXA6 | -0.708187086 | 7.75173777083333 | -7.426469941 | 1.34812832141166e-12 | 7.94194302211003e-11 | 18.1458023812757 |
| MYBPC1 | 0.750837765527102 | 8.67089956115942 | 7.41637250964542 | 1.43682676301662e-12 | 8.39525904074253e-11 | 18.0839937883733 |
| RAD51C | -0.684536154 | 7.15072178355072 | -7.40905422 | 1.50468640225671e-12 | 8.68321704479255e-11 | 18.0392319619738 |
| PBXIP1 | 0.751594938559863 | 8.25146302721014 | 7.37624201642258 | 1.84990131805234e-12 | 1.04601476544548e-10 | 17.8389011991465 |
| GSN | 0.650173517184917 | 7.4831719065942 | 7.36868139817796 | 1.93991068543066e-12 | 1.09101413561076e-10 | 17.7928251290298 |
| MAEL | -0.931095919 | 5.5819354048913 | -7.362001262 | 2.02302264651513e-12 | 1.12129828348149e-10 | 17.7521412551608 |
| FXYD7 | -0.832766187 | 7.79557113278986 | -7.359604161 | 2.05369463363815e-12 | 1.13381731683102e-10 | 17.7375482819864 |
| FUCA1 | -0.615134498 | 6.88952038416667 | -7.3498452 | 2.18336946185571e-12 | 1.18672054122491e-10 | 17.6781709898214 |
| PTPN3 | -0.787911941 | 6.27461525677536 | -7.344264996 | 2.26111193221367e-12 | 1.22423060329854e-10 | 17.6442426390942 |
| NIT2 | -0.720402695 | 7.51218173894928 | -7.339099636 | 2.33550422027961e-12 | 1.25964521849927e-10 | 17.6128520295148 |
| ANLN | 0.762798118120826 | 8.28217175442029 | 7.33786023929336 | 2.35371017599534e-12 | 1.26460068191504e-10 | 17.6053222490185 |
| STOM | 0.685950050625687 | 9.50759042771739 | 7.33430089523277 | 2.4067762528442e-12 | 1.28327845603172e-10 | 17.5837027153074 |
| SLC16A14 | -0.642641469 | 7.87850842847826 | -7.332393458 | 2.435697825925e-12 | 1.29377994746009e-10 | 17.5721198014893 |
| TSPAN7 | -0.637734302 | 11.5244052319928 | -7.326972417 | 2.51977846097494e-12 | 1.32340274750006e-10 | 17.5392116035112 |
| BSCL2 | -0.963455406 | 10.1818062076812 | -7.319865958 | 2.63434744327696e-12 | 1.37328826011423e-10 | 17.4960969632228 |
| PGF | 0.840673246239557 | 8.39541385068841 | 7.30547149109996 | 2.88237659908052e-12 | 1.48601349444508e-10 | 17.4088526382964 |
| MAL2 | -1.089151197 | 8.18777132561594 | -7.297644173 | 3.02676873265794e-12 | 1.52677618482238e-10 | 17.3614601960056 |
| TOB2 | 0.610230436920972 | 7.70645062971014 | 7.29411953651524 | 3.09409486211592e-12 | 1.55514309144988e-10 | 17.3401306088141 |
| ANGPT1 | 0.608134705001837 | 6.65406800503623 | 7.29069774615973 | 3.16086803534057e-12 | 1.58303044498503e-10 | 17.3194300644951 |
| DTX3L | 0.728711658730743 | 6.80811348768116 | 7.28951598079366 | 3.18425755045149e-12 | 1.58906916832673e-10 | 17.3122823536985 |
| SCN2B | -0.889940848 | 7.70811551565217 | -7.288601634 | 3.20247130507156e-12 | 1.59249131599357e-10 | 17.3067526179926 |
| NDRG4 | -0.656060421 | 10.0933098693116 | -7.271925754 | 3.55325716357216e-12 | 1.74832720016745e-10 | 17.205983471349 |
| PRR11 | 0.729553577941012 | 8.82839807673913 | 7.26731289283736 | 3.65680470898983e-12 | 1.78673771547611e-10 | 17.1781364301866 |
| SULT4A1 | -0.844434653 | 9.47065078050725 | -7.262797182 | 3.76105070017342e-12 | 1.82495550064124e-10 | 17.150887466792 |
| VASN | 0.729633596543984 | 7.32705643663043 | 7.25924296290163 | 3.84515564591056e-12 | 1.85294218634377e-10 | 17.1294484703964 |
| EMX2 | 0.845523487850574 | 8.60096252315217 | 7.25046562599556 | 4.06088105543697e-12 | 1.9435404450646e-10 | 17.0765342047879 |
| RRAGB | -1.055759308 | 6.10562387804348 | -7.239419954 | 4.34937235139522e-12 | 2.03984108640853e-10 | 17.0100070351606 |
| FGR | 0.930838613174197 | 6.58099852855072 | 7.23008693715323 | 4.60880510359766e-12 | 2.14715195906146e-10 | 16.9538487905861 |
| SDHB | -0.664767029 | 7.78788424902174 | -7.227390121 | 4.68656893548093e-12 | 2.17615086696189e-10 | 16.9376307967222 |
| MEGF10 | 0.710230615951414 | 8.77243674253623 | 7.21753605644147 | 4.98186693300427e-12 | 2.29805000004996e-10 | 16.8784058894944 |
| RTN4IP1 | -0.938209142 | 6.28987604344203 | -7.213206847 | 5.11732828060576e-12 | 2.35279654029294e-10 | 16.8524038759128 |
| SV2B | -0.842221019 | 10.2810268760145 | -7.192042084 | 5.8336198350642e-12 | 2.64740617951797e-10 | 16.7254377199365 |
| DHRS7B | -1.1051448 | 6.49321969782609 | -7.177967761 | 6.36373481268812e-12 | 2.8786662347847e-10 | 16.6411478260658 |
| LARGE | -0.696484788 | 8.07920356021739 | -7.177290908 | 6.39039136093023e-12 | 2.88142951943166e-10 | 16.6370970540771 |
| RTN3 | -0.684960746 | 10.5992916800362 | -7.157849223 | 7.20483354308035e-12 | 3.19725888527265e-10 | 16.5208559495372 |
| MPV17 | -0.867787975 | 7.42389526637681 | -7.155256695 | 7.32089143255601e-12 | 3.23851295137959e-10 | 16.5053716579688 |
| GOT1 | -0.981638792 | 9.18258750275362 | -7.153879101 | 7.38330857537162e-12 | 3.25585333812692e-10 | 16.497145317643 |
| ENO2 | -0.63514726 | 10.9961772130072 | -7.14828206 | 7.642340728213e-12 | 3.35951548688812e-10 | 16.4637336167255 |
| PSD2 | 0.660526289307017 | 9.94523419452899 | 7.14062579887539 | 8.01122520544237e-12 | 3.48717312502321e-10 | 16.4180584283326 |
| AEBP1 | 0.945101741050012 | 7.91928280286232 | 7.14020074455515 | 8.03221079214502e-12 | 3.48717312502321e-10 | 16.4155236539557 |
| DHPS | -0.626456152 | 8.01637336684783 | -7.132726442 | 8.41020179415881e-12 | 3.61767667973893e-10 | 16.3709682468089 |
| FAM107A | 0.784673432231969 | 12.4547495676087 | 7.1275755917377 | 8.6808344927094e-12 | 3.68980020570283e-10 | 16.340281958541 |
| CAPNS1 | -0.791901817 | 9.10048786543478 | -7.127532749 | 8.68312107168177e-12 | 3.68980020570283e-10 | 16.3400267889782 |
| MAP2K4 | -0.714758792 | 8.83120090681159 | -7.12234712 | 8.96431561622166e-12 | 3.7863433098276e-10 | 16.30914884 |
| TM7SF2 | -0.793054259 | 7.51330516061594 | -7.120594955 | 9.06134058881099e-12 | 3.80440655918852e-10 | 16.2987190260871 |
| SLC35E1 | 0.637515757593462 | 9.47878371271739 | 7.11648365400645 | 9.2930804496584e-12 | 3.87847818885594e-10 | 16.2742533320449 |
| OSMR | 0.671440398623475 | 6.43180986833333 | 7.10887567469037 | 9.73741385549301e-12 | 4.03668308281484e-10 | 16.2290051425436 |
| GRAMD1C | 0.80567457694306 | 7.76834940811594 | 7.10852305373871 | 9.75850791609664e-12 | 4.03668308281484e-10 | 16.2269087507803 |
| FHL2 | -0.699340599 | 7.69458229992754 | -7.099271385 | 1.03283178488017e-11 | 4.23491231560662e-10 | 16.1719316278321 |
| HSPB3 | -1.042860933 | 6.21861548612319 | -7.092276911 | 1.0780741303045e-11 | 4.40753164118366e-10 | 16.1304004638975 |
| FYCO1 | 0.627941384825172 | 7.11808043213768 | 7.05147412735641 | 1.38363777491728e-11 | 5.45021138136659e-10 | 15.8886899496135 |
| TBC1D7 | -0.667168728 | 7.17740361800725 | -7.048264816 | 1.41100618539352e-11 | 5.49626103827039e-10 | 15.8697193938769 |
| KCNIP4 | -0.645774758 | 8.12107772166667 | -7.03968477 | 1.48681981830875e-11 | 5.75957853926618e-10 | 15.8190313281797 |
| KCNJ10 | 0.768115652403392 | 8.43800197746377 | 7.03661988551418 | 1.51486205022528e-11 | 5.83596443140359e-10 | 15.8009354034742 |
| SLC12A7 | 0.644086505342548 | 7.78381603706522 | 7.03489098507614 | 1.53090971012867e-11 | 5.84957680248892e-10 | 15.7907299122051 |
| FBXW12 | 0.650096679870135 | 8.24768084655797 | 7.03409114743862 | 1.53839031903829e-11 | 5.86218680540053e-10 | 15.7860091564544 |
| TUSC3 | -0.623342498 | 7.23923720177536 | -7.032275342 | 1.55550676939191e-11 | 5.9033744690196e-10 | 15.7752934015186 |
| PLCL2 | -0.608295884 | 7.70554504855073 | -7.019882291 | 1.67744013832292e-11 | 6.28950349189901e-10 | 15.702208679518 |
| LRP10 | 0.595486760314949 | 7.69670351228261 | 7.01219381214899 | 1.75776563922945e-11 | 6.53823542676778e-10 | 15.6569130183336 |
| CNOT10 | -0.830425197 | 5.86730088061594 | -7.009504945 | 1.7867418465618e-11 | 6.62843410432174e-10 | 15.6410800699541 |
| AHCYL1 | 0.585348968161318 | 11.0797948093478 | 6.98680139735116 | 2.05090590114988e-11 | 7.47009180566879e-10 | 15.5075628803794 |
| GEM | 0.852563337112887 | 7.06908522083333 | 6.96927854686296 | 2.28074269995601e-11 | 8.26430358694654e-10 | 15.4047197390201 |
| RWDD2B | -0.778189516 | 6.91614346865942 | -6.968544838 | 2.29090017475459e-11 | 8.27971472953185e-10 | 15.4004174787414 |
| HSPA2 | 0.793109401097849 | 9.55920082478261 | 6.96194934469757 | 2.38423019834508e-11 | 8.55091050419259e-10 | 15.3617576041282 |
| C1QTNF5 | 0.607871604796259 | 7.29480905456522 | 6.95578260925391 | 2.47487299879422e-11 | 8.78611242078264e-10 | 15.3256341131375 |
| NSUN6 | 0.700724379366424 | 8.14762186597826 | 6.95288737995583 | 2.51859067314728e-11 | 8.9187366185718e-10 | 15.3086821822831 |
| RPH3A | -0.795013125 | 8.60818967130435 | -6.945932212 | 2.62674273428586e-11 | 9.27829051961981e-10 | 15.2679790225655 |
| RAB3C | -0.830214796 | 7.4988103776087 | -6.944961687 | 2.64219316009523e-11 | 9.30941574975261e-10 | 15.2623015694409 |
| GNG3 | -0.787094636 | 9.91198418021739 | -6.941703105 | 2.69472574752848e-11 | 9.47071156831876e-10 | 15.2432433352168 |
| SENP8 | -0.633676644 | 5.67609709210145 | -6.928475611 | 2.91872957941925e-11 | 1.02068191751113e-09 | 15.1659451348403 |
| EMP1 | 0.662764171381253 | 6.51093508224638 | 6.92256473039443 | 3.0246591057009e-11 | 1.04727887998133e-09 | 15.1314369040669 |
| NECAP1 | -0.794077281 | 9.31169038376812 | -6.917786737 | 3.11304305355474e-11 | 1.07522666847286e-09 | 15.1035577009039 |
| CAMK1G | -0.867425779 | 7.23172571753623 | -6.90188179 | 3.42595939224132e-11 | 1.17176167213171e-09 | 15.0108512731197 |
| SERPINB6 | 0.641522750507911 | 8.30078446894928 | 6.894170875 | 3.58860661011054e-11 | 1.21553213752609e-09 | 14.9659601675691 |
| MECR | -0.790293282 | 6.77714386677536 | -6.861097413 | 4.37676858525441e-11 | 1.46131966359578e-09 | 14.7738163853361 |
| ZBTB20 | 0.740793234146899 | 9.04579990405797 | 6.85247412463406 | 4.60882203167363e-11 | 1.5242809280698e-09 | 14.7238258991952 |
| GABRG1 | 0.704092566492987 | 7.75278237351449 | 6.84237733280536 | 4.89593796801895e-11 | 1.61542913236541e-09 | 14.6653499017904 |
| KIFAP3 | -0.942303476 | 8.97042085858696 | -6.838927219 | 4.9980232199965e-11 | 1.64524130549322e-09 | 14.6453824336562 |
| GABRA1 | -0.976346011 | 8.65745931213768 | -6.837830373 | 5.03091416493957e-11 | 1.65218991416739e-09 | 14.6390359542559 |
| BMPR1B | 0.596505537544562 | 7.63891763362319 | 6.83430100638329 | 5.13819984565902e-11 | 1.68348075784291e-09 | 14.6186195312344 |
| MEF2C | -0.756994533 | 10.2167956218478 | -6.823771189 | 5.47183395726399e-11 | 1.78031386502814e-09 | 14.5577518747253 |
| ATP1A3 | -0.90397726 | 7.97008139322464 | -6.821764663 | 5.53778450252094e-11 | 1.79344923969633e-09 | 14.5461607008644 |
| ATP6V1H | -0.645271661 | 8.81758226862319 | -6.817737661 | 5.67251146999965e-11 | 1.83284857935035e-09 | 14.5229050814744 |
| SLC38A2 | 0.626927170617799 | 10.4902992077536 | 6.80873074937803 | 5.98562072775531e-11 | 1.90764453330256e-09 | 14.4709262237521 |
| CASP7 | 0.722151456847891 | 6.30198450242754 | 6.79903388053604 | 6.34172042273199e-11 | 2.01198971692241e-09 | 14.4150202887438 |
| COL5A3 | 0.644137147746978 | 7.45307071231884 | 6.79497788858311 | 6.49677478875497e-11 | 2.05652986146075e-09 | 14.3916528680385 |
| RHOBTB3 | 0.618082043529107 | 9.21680495376812 | 6.78953733349729 | 6.71062550333163e-11 | 2.11467643670156e-09 | 14.3603242924797 |
| DDX25 | -0.632074541 | 7.91594752637681 | -6.78656269 | 6.83046020862278e-11 | 2.14761308308335e-09 | 14.3432028440411 |
| RAB3B | -0.640933902 | 6.32360268192029 | -6.773690369 | 7.37373928282152e-11 | 2.30807915095996e-09 | 14.2691740597578 |
| C1QTNF4 | -1.200044154 | 6.83269328456522 | -6.770835278 | 7.49987701473885e-11 | 2.3423335273426e-09 | 14.2527680155782 |
| CFI | 0.691243556420949 | 5.60362194858696 | 6.7687477941785 | 7.59344206290406e-11 | 2.36103853765197e-09 | 14.2407759506866 |
| REEP1 | -0.598539659 | 9.73803842166667 | -6.76395508 | 7.81262423972616e-11 | 2.41313721836299e-09 | 14.2132530238763 |
| GLOD4 | -0.596246579 | 7.9644059076087 | -6.761638572 | 7.92078152994382e-11 | 2.44116745921763e-09 | 14.1999551016389 |
| DDIT4L | 0.791357247423626 | 7.23932119257246 | 6.75597016539899 | 8.19168444742655e-11 | 2.50812207437255e-09 | 14.1674293344626 |
| STAT4 | -0.914551559 | 7.52151129833333 | -6.754281004 | 8.27415944786892e-11 | 2.5278548570254e-09 | 14.1577405640637 |
| FIG4 | -0.612332861 | 8.8503514023913 | -6.748616753 | 8.55672446922886e-11 | 2.59720665004321e-09 | 14.1252639520163 |
| CTDSP1 | 0.669853998105572 | 8.03641924134058 | 6.74810819423567 | 8.58255351104826e-11 | 2.59942004072203e-09 | 14.1223490256469 |
| ZNF160 | 0.686394343558026 | 8.37430303003623 | 6.74203504168936 | 8.89698621676682e-11 | 2.66797253084225e-09 | 14.0875514780578 |
| FAM89A | 0.686877946429366 | 7.57401157833333 | 6.74179299692627 | 8.90974977615306e-11 | 2.66797253084225e-09 | 14.0861650910049 |
| SYT13 | -0.758313062 | 9.86969357963768 | -6.73703487 | 9.16434246760885e-11 | 2.71871248465623e-09 | 14.0589186710986 |
| BFSP1 | -0.703378588 | 5.80620833855073 | -6.736925112 | 9.17029883222134e-11 | 2.71871248465623e-09 | 14.0582903311922 |
| NUPR1 | 0.809152276424104 | 7.79471185807971 | 6.72851076377512 | 9.63844161510668e-11 | 2.82761227549458e-09 | 14.0101416466194 |
| RHPN2 | 0.746741606926766 | 7.80799795007246 | 6.71729840402282 | 1.02989244849178e-10 | 3.00253259983372e-09 | 13.9460492530841 |
| CRB2 | 0.878162822616858 | 5.83284633967391 | 6.71105511733803 | 1.06857697447954e-10 | 3.08961956971683e-09 | 13.9103944819224 |
| AQP4 | 0.761825717345816 | 10.1650055311594 | 6.69759251958797 | 1.1569179031547e-10 | 3.31768093168474e-09 | 13.8335920796715 |
| GPRC5B | 0.621728698398437 | 10.2571518511594 | 6.69101310154792 | 1.20266449463862e-10 | 3.43800081057731e-09 | 13.7960977085067 |
| GOT2 | -0.611542354 | 10.2383993514855 | -6.690484054 | 1.20641922489677e-10 | 3.43800081057731e-09 | 13.7930839598454 |
| SDC4 | 0.780795009814925 | 9.43978457068841 | 6.6843126045935 | 1.25107898836797e-10 | 3.51580774626934e-09 | 13.7579406161026 |
| NRN1 | -0.656787517 | 9.50704922068841 | -6.676980866 | 1.30624982589275e-10 | 3.6635082616988e-09 | 13.716220380586 |
| RGS7 | -0.734391108 | 9.0515862398913 | -6.673484413 | 1.33339546723134e-10 | 3.73194912286713e-09 | 13.6963359309254 |
| GABRG2 | -0.790825626 | 9.39533434130435 | -6.660448488 | 1.43957681903393e-10 | 3.98255005491704e-09 | 13.6222664165634 |
| IL17RB | 0.663548759105105 | 7.8372063292029 | 6.6477593162497 | 1.55089604194784e-10 | 4.23117027164096e-09 | 13.5502676359622 |
| SERPINA3 | 1.35255524458016 | 8.93185770619565 | 6.64677651504436 | 1.55986252230036e-10 | 4.23945712416022e-09 | 13.5446953311213 |
| ATP1A1 | -0.681105663 | 10.5704157832609 | -6.646763681 | 1.55997994442464e-10 | 4.23945712416022e-09 | 13.5446225708922 |
| PSAT1 | 0.637508103856142 | 9.50502469463768 | 6.64487938049693 | 1.57731509018184e-10 | 4.2782765008936e-09 | 13.5339406071885 |
| HEPH | 0.7097126519796 | 7.75938051594203 | 6.64384252734726 | 1.58693447596862e-10 | 4.29605833137218e-09 | 13.5280636972997 |
| PTPRR | -0.587788068 | 7.44120550630435 | -6.640622231 | 1.61717964429189e-10 | 4.35272747637335e-09 | 13.50981521 |
| FOXC1 | 0.968787209114565 | 8.70686561213768 | 6.63996320154474 | 1.62343871662154e-10 | 4.36120328030343e-09 | 13.5060814664362 |
| SYN2 | -0.742391606 | 9.00738778927536 | -6.638003147 | 1.64219513032105e-10 | 4.38638139285563e-09 | 13.4949783370237 |
| VASP | 0.742259700859144 | 6.15689030985507 | 6.635812795 | 1.66340725953811e-10 | 4.43168353571512e-09 | 13.4825734450477 |
| SLCO4A1 | 0.826860605067036 | 7.02713214786232 | 6.63560063193218 | 1.66547616296218e-10 | 4.43168353571512e-09 | 13.4813720323893 |
| MTX2 | -0.764580729 | 8.36472452539855 | -6.625760728 | 1.76425784405245e-10 | 4.67678407318478e-09 | 13.4256823639315 |
| SLC25A18 | 0.831313544401915 | 10.2798946012319 | 6.62489953590985 | 1.77317222033039e-10 | 4.68411794895781e-09 | 13.4208112334782 |
| TJP2 | 0.591321570016301 | 7.41861277905797 | 6.62484819286279 | 1.77370507801227e-10 | 4.68411794895781e-09 | 13.420520838087 |
| SLC14A1 | 0.917828734089592 | 8.0587897567029 | 6.62253544096625 | 1.79787150641429e-10 | 4.73901355910668e-09 | 13.4074416450476 |
| GLUL | 0.624925331776634 | 10.6432427745652 | 6.62036398973818 | 1.82085568271058e-10 | 4.77268396984122e-09 | 13.3951645565719 |
| LAMB2 | 0.734481739248648 | 7.90285826597826 | 6.61860275615178 | 1.83970958780888e-10 | 4.7952132992275e-09 | 13.3852089269991 |
| PAK1 | -0.631347456 | 9.27272011967391 | -6.610935763 | 1.92404313500873e-10 | 4.97801787494972e-09 | 13.3418925247355 |
| CASD1 | -0.612445881 | 6.96250583644928 | -6.610140196 | 1.9330084791037e-10 | 4.99200329695601e-09 | 13.3373998756201 |
| MSX1 | 0.690852425282091 | 7.3717587815942 | 6.58901895289602 | 2.18661848801908e-10 | 5.58523698679262e-09 | 13.2182697139855 |
| PECAM1 | 0.657541348249651 | 7.47092942710145 | 6.58815534919915 | 2.19765546864979e-10 | 5.60322229761383e-09 | 13.213404628151 |
| ATOH7 | -0.717769226 | 5.59364375213768 | -6.584838004 | 2.24056260841056e-10 | 5.70225217018898e-09 | 13.1947207789952 |
| JAZF1 | -0.615986447 | 7.5289113109058 | -6.58055443 | 2.29718587603292e-10 | 5.81938517704921e-09 | 13.1706050906271 |
| UQCRC1 | -0.856660809 | 9.43036137873188 | -6.570494614 | 2.43574647636651e-10 | 6.14325051044739e-09 | 13.114015229517 |
| SSSCA1 | -0.941898843 | 5.84929300449275 | -6.563046658 | 2.54358749176858e-10 | 6.3922450532385e-09 | 13.0721586092732 |
| CD163 | 0.931215787683371 | 5.90113324619565 | 6.5625442570289 | 2.55102835061931e-10 | 6.39947595004197e-09 | 13.0693364231007 |
| BCAR3 | 0.648495601353386 | 7.01895740804348 | 6.56173748132542 | 2.56302192415262e-10 | 6.41316944952054e-09 | 13.064804771479 |
| TM2D2 | -0.599823844 | 8.33475301166667 | -6.561255936 | 2.57020696757509e-10 | 6.41316944952054e-09 | 13.0621001308362 |
| ENTPD2 | 0.691653604379833 | 5.51526230615942 | 6.55716808515474 | 2.63200290296277e-10 | 6.54407388444095e-09 | 13.0391461855687 |
| FOXD1 | 1.02853073506336 | 5.5691573782971 | 6.55062601865774 | 2.73394721002043e-10 | 6.77743963213559e-09 | 13.0024331481963 |
| HPRT1 | -0.822002321 | 9.09620553384058 | -6.546331458 | 2.80296623912834e-10 | 6.92006964283393e-09 | 12.9783473045006 |
| SNX31 | 1.09683568226458 | 5.36881328938406 | 6.54601009761021 | 2.80819915758803e-10 | 6.92080435621389e-09 | 12.9765454341479 |
| LRMP | -0.740071844 | 6.01425328702899 | -6.541132122 | 2.88881815608355e-10 | 7.09455289015055e-09 | 12.9492025141012 |
| PODXL2 | -0.798197208 | 7.45394841018116 | -6.530877975 | 3.06576963651563e-10 | 7.43793903336656e-09 | 12.8917726837049 |
| LETMD1 | -0.634111785 | 8.4901545426087 | -6.527999313 | 3.11732987299308e-10 | 7.54996836079137e-09 | 12.8756621685578 |
| SLC1A3 | 0.64690064345392 | 11.5579684298913 | 6.52312717675884 | 3.20654175069899e-10 | 7.75264396035378e-09 | 12.8484069663895 |
| EFEMP2 | 0.585499668772284 | 8.077890015 | 6.51750389204231 | 3.31262586354416e-10 | 7.97341910072817e-09 | 12.8169682876132 |
| WDR7 | -0.672452681 | 8.74918208492754 | -6.515571217 | 3.34987559895509e-10 | 8.02996675626448e-09 | 12.8061676694147 |
| EPS8 | 0.672432496131764 | 7.732214953 | 6.51333719839041 | 3.3934458265889e-10 | 8.10669349680685e-09 | 12.7936859343402 |
| ATRNL1 | -0.638909696 | 7.76316478695652 | -6.509440472 | 3.47077905765084e-10 | 8.27733583765947e-09 | 12.7719219592626 |
| PPP1R14C | -0.811032725 | 6.97943371945652 | -6.501438154 | 3.63505369311202e-10 | 8.6105334355591e-09 | 12.7272574032467 |
| TUBA4A | -0.712232148 | 10.6724346391667 | -6.50028268 | 3.65939564300951e-10 | 8.65357590251641e-09 | 12.7208115127192 |
| TAGLN3 | -0.623487592 | 10.7072530378261 | -6.499970276 | 3.66600438154255e-10 | 8.65460933373251e-09 | 12.7190688877404 |
| STMN2 | -0.866605278 | 11.1531930680072 | -6.498151922 | 3.70470379847095e-10 | 8.72500250354556e-09 | 12.7089271336832 |
| NVL | -0.929438316 | 5.86163456184783 | -6.497985482 | 3.70826605727245e-10 | 8.72500250354556e-09 | 12.7079989271572 |
| HSPA1A | 0.819709541153586 | 11.3555900826087 | 6.49736853801848 | 3.72149962581793e-10 | 8.74147223665743e-09 | 12.7045584976439 |
| ELF1 | 0.649511552713601 | 6.56347272818841 | 6.49678650720979 | 3.73402678125183e-10 | 8.75623036011612e-09 | 12.7013129844071 |
| GABRA4 | -0.706377114 | 7.11010772876812 | -6.485303558 | 3.98981208130623e-10 | 9.27846348526654e-09 | 12.6373255605198 |
| CDC37 | -0.901191851 | 7.95023396032609 | -6.482706393 | 4.05000664343338e-10 | 9.38731291915146e-09 | 12.6228646881721 |
| PPIH | -0.659246503 | 7.12646039311594 | -6.480819271 | 4.09430268384523e-10 | 9.45899855865489e-09 | 12.612359966627 |
| TMEM123 | 0.675157687989911 | 8.72623243927536 | 6.48081408943276 | 4.09442496263533e-10 | 9.45899855865489e-09 | 12.6123311261865 |
| PON2 | 0.676871362439147 | 10.1621142217029 | 6.4800520648607 | 4.11244678893542e-10 | 9.4850067962568e-09 | 12.6080899330654 |
| MRPS9 | -0.590666301 | 7.87197382764493 | -6.47567003 | 4.21760253504727e-10 | 9.69564595884719e-09 | 12.5837080027454 |
| MRVI1 | 0.663534005690627 | 8.32796172605073 | 6.46899645931816 | 4.38283665944993e-10 | 1.00590046604691e-08 | 12.5465991197475 |
| BDNF | -0.747452013 | 6.49156459641304 | -6.457394848 | 4.68533510135696e-10 | 1.06833258741998e-08 | 12.482154561747 |
| SEMA3F | 0.706530368247019 | 6.97909836438406 | 6.45457806624128 | 4.76181591304517e-10 | 1.0840088400752e-08 | 12.4665207791792 |
| CARTPT | -0.776682664 | 7.52255615188406 | -6.438928212 | 5.20957845017798e-10 | 1.17639160397497e-08 | 12.3797521216361 |
| C1orf87 | 0.785720927608182 | 4.70597209978261 | 6.43634710575379 | 5.28729296756077e-10 | 1.19202104958368e-08 | 12.3654564296951 |
| KCTD4 | -0.767407942 | 6.7661871909058 | -6.431425883 | 5.43863031317638e-10 | 1.22040094945679e-08 | 12.338211521218 |
| PSMG1 | -0.67149638 | 7.16667177583333 | -6.431404965 | 5.43928256015472e-10 | 1.22040094945679e-08 | 12.3380957488013 |
| PSMD8 | -0.983238075 | 8.58168630192029 | -6.42942272 | 5.50144123911576e-10 | 1.23237556703068e-08 | 12.3271260083207 |
| NIF3L1 | -0.608849067 | 8.00770573518116 | -6.426762082 | 5.58596931285042e-10 | 1.24732559990607e-08 | 12.312405964856 |
| TARBP1 | -0.778967903 | 8.00826930036232 | -6.417184255 | 5.90093632130058e-10 | 1.30931693091136e-08 | 12.2594536995115 |
| CYBRD1 | 0.674369578721279 | 8.37436084384058 | 6.41275490238749 | 6.05242459782761e-10 | 1.34080805901006e-08 | 12.2349852004512 |
| NLRC5 | 0.791595252705714 | 6.67494635336956 | 6.40388602756272 | 6.36730375250127e-10 | 1.40170644460479e-08 | 12.1860296112659 |
| SLC4A11 | 0.597768554691624 | 6.79492016173913 | 6.400348629 | 6.49732913256591e-10 | 1.42591071006148e-08 | 12.1665174060388 |
| SGIP1 | -0.830581517 | 8.68475995431159 | -6.400341621 | 6.49758927283238e-10 | 1.42591071006148e-08 | 12.1664787613232 |
| COLEC12 | 0.720211465034963 | 7.32786014166667 | 6.39565199214223 | 6.6739963248843e-10 | 1.46196940336811e-08 | 12.1406232187761 |
| ASNS | -0.67885277 | 9.12750188195652 | -6.394449044 | 6.71999872421253e-10 | 1.46554497837686e-08 | 12.1339932121773 |
| ABCA1 | 0.61269502524423 | 7.75150328318841 | 6.37707920762497 | 7.41995427555347e-10 | 1.59341529565216e-08 | 12.0383631295772 |
| ATP1A2 | 0.703791090403815 | 11.4168956640217 | 6.37310632157852 | 7.58983092729774e-10 | 1.62491907012971e-08 | 12.0165174357012 |
| GPS1 | -0.671858434 | 7.48639824677536 | -6.36506111 | 7.94560030398481e-10 | 1.68819928882999e-08 | 11.972310240621 |
| C10orf54 | 0.618848928809611 | 7.90202283474638 | 6.35474728281355 | 8.42568872923295e-10 | 1.76876396781488e-08 | 11.9156980891829 |
| TRIM47 | 0.768154726998268 | 6.70722055992754 | 6.33115373446693 | 9.63341145913618e-10 | 2.00429271352324e-08 | 11.7864512013292 |
| CDK5 | -0.848759825 | 8.57647614134058 | -6.329757699 | 9.70996219132104e-10 | 2.01722666383548e-08 | 11.7788148632287 |
| AMPH | -0.874113061 | 9.09542642144928 | -6.314479672 | 1.05876928436187e-09 | 2.17063182377288e-08 | 11.6953259295522 |
| RASEF | 0.720447916985123 | 8.11446773630435 | 6.30477759414016 | 1.1184975559653e-09 | 2.28639813809058e-08 | 11.6423858002764 |
| TTLL1 | -0.665369421 | 6.95219923322464 | -6.299251029 | 1.15398398134111e-09 | 2.34526338700673e-08 | 11.6122568803577 |
| GBP1 | 0.720152050025764 | 6.10398165043478 | 6.2911685434311 | 1.2078771840269e-09 | 2.44064290369009e-08 | 11.5682295629918 |
| SLC27A4 | -0.748941155 | 7.0472185209058 | -6.283922083 | 1.25828794595552e-09 | 2.53155980862758e-08 | 11.5287922650207 |
| NUDT18 | -0.758404425 | 6.54747115786232 | -6.279538875 | 1.28977495347979e-09 | 2.58748414487083e-08 | 11.5049541227822 |
| TMEM35 | -0.596138914 | 7.2710679909058 | -6.27697124 | 1.30857697980336e-09 | 2.61369637772e-08 | 11.4909957934461 |
| EFNA1 | 0.610748816597617 | 7.9973556301087 | 6.27488436580015 | 1.32405618344273e-09 | 2.63739202562747e-08 | 11.4796541543334 |
| CDK7 | -0.874124097 | 7.09517813271739 | -6.271018909 | 1.35320326413875e-09 | 2.68781435878437e-08 | 11.4586538382001 |
| ELMO1 | -0.703512972 | 7.61667688 | -6.270217693 | 1.35932295392453e-09 | 2.69615074722541e-08 | 11.4543021916812 |
| SH2D2A | 0.63175416158263 | 5.71639848992754 | 6.26758168998292 | 1.37964875190857e-09 | 2.72874674866203e-08 | 11.4399882075425 |
| ATP6AP1 | -0.719369611 | 9.78296675489131 | -6.253342929 | 1.49469876718305e-09 | 2.94384281070337e-08 | 11.3627472189548 |
| GABRD | -0.970583515 | 7.2564796690942 | -6.247962782 | 1.54057448545267e-09 | 3.01724525272386e-08 | 11.3335958966233 |
| MT1X | 0.721462448457331 | 10.5879809301812 | 6.24664293192555 | 1.55203771297167e-09 | 3.03545674323594e-08 | 11.3264474139333 |
| ANKRD39 | -0.727663879 | 7.07616492268116 | -6.245769674 | 1.55966807066703e-09 | 3.04613166503673e-08 | 11.3217183574144 |
| SCN2A | -0.595434259 | 10.1739771999275 | -6.23790891 | 1.63002917751243e-09 | 3.16591401056188e-08 | 11.2791714226204 |
| PPEF1 | -0.901156416 | 5.69665920688406 | -6.222647801 | 1.7756213551018e-09 | 3.41089565240993e-08 | 11.1966847547909 |
| NEK7 | 0.607377301954892 | 7.82419536101449 | 6.218464245 | 1.81770924809676e-09 | 3.48696809658835e-08 | 11.1740991100537 |
| CECR6 | -0.751299543 | 7.07747498699275 | -6.217411723 | 1.82845087149158e-09 | 3.49899854408174e-08 | 11.1684186907824 |
| LEAP2 | 0.722078482251433 | 6.25288383485507 | 6.21101398575974 | 1.89509402144188e-09 | 3.60440131853418e-08 | 11.1339059606722 |
| TCIRG1 | 0.916730232900785 | 5.93721169572464 | 6.20461673080727 | 1.96410932473007e-09 | 3.7069589583701e-08 | 11.0994226282293 |
| PSMC3 | -0.758309491 | 7.92341531702899 | -6.188319958 | 2.15123904921921e-09 | 4.03299801967927e-08 | 11.0116988361094 |
| CCK | -0.948347117 | 9.22920708452899 | -6.184680908 | 2.19535111340756e-09 | 4.10472115510856e-08 | 10.9921340269134 |
| ZCCHC12 | -0.679120211 | 8.24839539742754 | -6.177016734 | 2.29117230973717e-09 | 4.24426807126081e-08 | 10.9509572026489 |
| CALB1 | -0.774097552 | 7.0969665684058 | -6.174891041 | 2.31846642086397e-09 | 4.2860039251205e-08 | 10.9395434711858 |
| MID1IP1 | 0.587512080169834 | 8.13056275344203 | 6.17095767078651 | 2.3698134809804e-09 | 4.3611410031218e-08 | 10.9184313999117 |
| GAD2 | -0.652670918 | 6.93793504351449 | -6.165789329 | 2.43897793204928e-09 | 4.47082190080093e-08 | 10.8907061989722 |
| PLK2 | -0.876351671 | 8.82838538047101 | -6.155655795 | 2.58037295992748e-09 | 4.68131004519015e-08 | 10.8363966493965 |
| SERPING1 | 0.603695259579371 | 7.75288248413043 | 6.15425324732832 | 2.60056468708204e-09 | 4.71159155128571e-08 | 10.8288851857305 |
| CDH18 | -0.638453424 | 8.38944499101449 | -6.149324858 | 2.67275043695585e-09 | 4.82722664502556e-08 | 10.8025010694286 |
| RAB11FIP3 | 0.615494664131658 | 9.65855538463768 | 6.14783728477542 | 2.69492166871095e-09 | 4.85120494997864e-08 | 10.7945404978079 |
| ZIC1 | 0.837271802860824 | 7.04163471496377 | 6.13768411904671 | 2.85112991533805e-09 | 5.08095166365175e-08 | 10.7402460816541 |
| MRPL37 | -0.793218996 | 7.41177461786232 | -6.136447351 | 2.87075315622355e-09 | 5.10289704718256e-08 | 10.7336370730013 |
| GOLT1A | -0.881226767 | 4.57634466688406 | -6.134813343 | 2.89688205591715e-09 | 5.14214899621851e-08 | 10.7249068611542 |
| GLS | -0.634611008 | 9.37416162199275 | -6.12705806 | 3.02410310860757e-09 | 5.35441892575808e-08 | 10.6834958380773 |
| CLIP3 | -0.593408711 | 10.3313191042029 | -6.120367016 | 3.13824838032464e-09 | 5.50784193207665e-08 | 10.6477995497618 |
| ST6GALNAC5 | -0.70200748 | 8.04462927543478 | -6.115321392 | 3.22710254074702e-09 | 5.64964530947508e-08 | 10.6209010862274 |
| PRDM16 | 0.662877827348966 | 6.88754048387681 | 6.09249405667633 | 3.66084070613191e-09 | 6.30663012556361e-08 | 10.4994185689433 |
| ACTL6B | -0.841410777 | 8.07775732630435 | -6.082287629 | 3.87276196000988e-09 | 6.60678113932099e-08 | 10.4452140427159 |
| FAM107B | 0.745727568944739 | 9.11326383797102 | 6.0767381131095 | 3.99297988279158e-09 | 6.77888098019204e-08 | 10.4157706551887 |
| SMYD3 | -0.693859844 | 7.65866116322464 | -6.071965383 | 4.099285176279e-09 | 6.92290855258538e-08 | 10.3904649792643 |
| PPM1E | -0.875186986 | 8.64000939847826 | -6.071822903 | 4.10250089652603e-09 | 6.92290855258538e-08 | 10.3897097648764 |
| SORCS3 | -0.594710501 | 6.71605854387681 | -6.071432176 | 4.1113321462726e-09 | 6.92947243836306e-08 | 10.3876387842732 |
| NFIC | 0.682031078251743 | 9.02519644460145 | 6.06852196007882 | 4.17769688229273e-09 | 6.99926444210167e-08 | 10.3722169 |
| STAR | -0.748856849 | 5.90813341934783 | -6.059036646 | 4.40135831297002e-09 | 7.34764852652126e-08 | 10.3219913438483 |
| PDK4 | 0.713657009574116 | 8.2958169998913 | 6.05521366866896 | 4.49477879431394e-09 | 7.49468288141075e-08 | 10.3017653352836 |
| LAMB1 | -0.587851602 | 6.88517332391304 | -6.050580778 | 4.61059322352692e-09 | 7.63333515625951e-08 | 10.2772674543937 |
| NEUROD6 | -0.90418526 | 7.63717608619565 | -6.043546775 | 4.79203293519268e-09 | 7.87792237399846e-08 | 10.2401003626492 |
| BGN | 0.603761718448389 | 8.4457496167029 | 6.03600265852146 | 4.99439422670595e-09 | 8.18182128984785e-08 | 10.2002746723149 |
| ITGA6 | 0.598909030579415 | 6.73663449228261 | 6.02837481170325 | 5.20749364330803e-09 | 8.47154099305202e-08 | 10.1600457296971 |
| TFAP2C | 0.663853972998051 | 5.25734392771739 | 6.02763983295343 | 5.22849044523043e-09 | 8.48263484946253e-08 | 10.1561715428075 |
| UBE2T | -0.870375363 | 5.71866330634058 | -6.026710615 | 5.25515481789504e-09 | 8.5095884539656e-08 | 10.1512740079055 |
| TNFRSF13C | 0.615856294085912 | 5.40238481253623 | 6.01700678901519 | 5.54168007893467e-09 | 8.91372449366673e-08 | 10.1001636662939 |
| PCSK1 | -1.066996391 | 6.8029920807971 | -6.016066834 | 5.57023251831134e-09 | 8.92701378334628e-08 | 10.0952162502006 |
| APOO | -0.721822325 | 8.48012508985507 | -6.01126546 | 5.71834029455931e-09 | 9.14347616312488e-08 | 10.069953682517 |
| HDAC1 | 0.636915505157475 | 8.69947386619565 | 6.01022084381211 | 5.75107002372898e-09 | 9.18533655384413e-08 | 10.064459453863 |
| FSD1 | -0.716967253 | 8.30290086826087 | -6.000742275 | 6.0565471849905e-09 | 9.61845539922104e-08 | 10.0146398282188 |
| UCHL3 | -0.675251986 | 7.4883145576087 | -5.989387038 | 6.44344139050757e-09 | 1.01182954780613e-07 | 9.95503598280698 |
| ZNF134 | -0.629383607 | 6.07669694626812 | -5.978378265 | 6.84155631754143e-09 | 1.06480737226286e-07 | 9.89733368299254 |
| PLD3 | -0.683286503 | 8.96091578634058 | -5.974887285 | 6.97275218892072e-09 | 1.08282285653638e-07 | 9.87905284193213 |
| RNF175 | -0.605504068 | 8.67089647434783 | -5.973181936 | 7.03773267939966e-09 | 1.09049862279803e-07 | 9.87012561839113 |
| CLIC1 | 0.632576129209735 | 7.6721166373913 | 5.96368700490054 | 7.41049720904264e-09 | 1.14194947650994e-07 | 9.82045710327707 |
| SLC9A6 | -0.59896574 | 9.76640017478261 | -5.962881238 | 7.44300519394002e-09 | 1.14569991036906e-07 | 9.81624489777658 |
| CYP4X1 | -0.613721977 | 8.21281593721015 | -5.959586693 | 7.5773773133163e-09 | 1.16128483130748e-07 | 9.79902698156229 |
| LAMA5 | 0.620694692510133 | 8.73601700742754 | 5.95579004223195 | 7.73517095641207e-09 | 1.18288224996474e-07 | 9.77919407467595 |
| DMAP1 | -0.805112896 | 7.59589464858696 | -5.952395142 | 7.8789844612939e-09 | 1.20225243852801e-07 | 9.76146809001808 |
| RBP4 | -0.812840138 | 6.86601436768116 | -5.942171489 | 8.32804401226617e-09 | 1.26526718509218e-07 | 9.7081338949036 |
| CRH | -0.679322645 | 5.61493719608696 | -5.941804735 | 8.34460953280537e-09 | 1.26565397074814e-07 | 9.70622194422779 |
| NXPH1 | -0.664248233 | 7.27610778981884 | -5.941102517 | 8.37641710729192e-09 | 1.26849348915286e-07 | 9.70256142651217 |
| EXOSC9 | -0.61204266 | 7.30785156688406 | -5.930818482 | 8.85604336337028e-09 | 1.33249244725903e-07 | 9.64899118113633 |
| RAB27B | -0.72619237 | 8.09439392039855 | -5.928926506 | 8.94715845179913e-09 | 1.34331908961006e-07 | 9.63914356460256 |
| PFDN1 | -0.593050136 | 8.33855432271739 | -5.924939287 | 9.14218777121236e-09 | 1.36674732532741e-07 | 9.61839829804724 |
| TMEM141 | -0.593767567 | 7.49290206797101 | -5.918017065 | 9.49069173832681e-09 | 1.4098301932898e-07 | 9.58240803897498 |
| UBL7 | -0.752324941 | 7.40105966275362 | -5.914678386 | 9.66338808759809e-09 | 1.43093654859966e-07 | 9.56506110688484 |
| RGS4 | -0.770427387 | 9.80371026916667 | -5.913411489 | 9.72972005647284e-09 | 1.43469888908432e-07 | 9.55848061547067 |
| SYTL4 | 0.67198404029444 | 6.29236191985507 | 5.91278671702649 | 9.7625953580337e-09 | 1.43652544287205e-07 | 9.55523583999569 |
| NUPL2 | -0.677512937 | 6.86367236054348 | -5.906882102 | 1.00787034356494e-08 | 1.47684073435854e-07 | 9.52458312327478 |
| ZNF217 | 0.670648363355594 | 5.67171677456522 | 5.90177579203307 | 1.03601211932301e-08 | 1.51649247904662e-07 | 9.49809380199426 |
| AP2M1 | -0.684120541 | 9.81199474550725 | -5.900888087 | 1.04098214389749e-08 | 1.52217858226012e-07 | 9.49349058393753 |
| GAD1 | -0.724645087 | 8.86173851380435 | -5.899128855 | 1.05090053641719e-08 | 1.53508106481023e-07 | 9.48436963262498 |
| PDLIM1 | 0.715488170903835 | 6.93909917775362 | 5.8906163892894 | 1.10021236060687e-08 | 1.60210570433957e-07 | 9.44026551598447 |
| UHRF1 | 0.754224283916613 | 5.89078273235507 | 5.87513003902786 | 1.19578477829427e-08 | 1.73190559529595e-07 | 9.36015569122081 |
| OLFM3 | -0.740053278 | 7.5299957901087 | -5.870923243 | 1.22311754295955e-08 | 1.76221738335942e-07 | 9.33842253036127 |
| SMYD5 | -0.686851628 | 6.69594315862319 | -5.866839126 | 1.25023665301459e-08 | 1.79264504961387e-07 | 9.31733473468304 |
| MT1G | 0.687400808820653 | 9.61263732347826 | 5.86511772927366 | 1.26184212010216e-08 | 1.80615081696341e-07 | 9.3084499493959 |
| HSP90AB1 | -0.59664609 | 10.456579034058 | -5.856476775 | 1.32170694421065e-08 | 1.88356671531158e-07 | 9.26388134862331 |
| DUS4L | -0.620441581 | 5.85115086891304 | -5.845990193 | 1.39809213892878e-08 | 1.97834975420769e-07 | 9.20986203123847 |
| ENC1 | -0.898293277 | 11.1860295172101 | -5.837662554 | 1.46180722122e-08 | 2.05400026685051e-07 | 9.16701778532027 |
| PLXNB1 | 0.683955733448129 | 6.90028029583333 | 5.83613198017139 | 1.47382229580032e-08 | 2.06467632907171e-07 | 9.1591484284342 |
| GPAM | 0.612430093009106 | 7.81746882253623 | 5.82779889432504 | 1.54094862333074e-08 | 2.1502376651802e-07 | 9.11633260102087 |
| C11orf73 | -0.615494332 | 7.49814166083333 | -5.826115546 | 1.5548666443161e-08 | 2.1652328652676e-07 | 9.10768926410953 |
| PRMT7 | -0.83752062 | 6.40984568673913 | -5.822151474 | 1.58812846623733e-08 | 2.20498272099466e-07 | 9.08734300909817 |
| FAM19A1 | -0.648356412 | 8.93938950974638 | -5.813582553 | 1.66242041246131e-08 | 2.29450014212057e-07 | 9.04339855374791 |
| IGFBP7 | 0.63610447893107 | 10.0730386756159 | 5.80987581819161 | 1.69559908955717e-08 | 2.33799272692824e-07 | 9.02440477960869 |
| EXOSC5 | -0.706845857 | 6.64260976655797 | -5.800268827 | 1.78462854460904e-08 | 2.44393028135279e-07 | 8.97522143893175 |
| RAB31 | 0.588382763430259 | 10.3521563486957 | 5.79569380063641 | 1.8286144303486e-08 | 2.49928461567041e-07 | 8.95182180398557 |
| PSMC4 | -0.637864289 | 7.57029485978261 | -5.772406202 | 2.06943605175384e-08 | 2.77435007205967e-07 | 8.83293817983057 |
| PLCD1 | 0.624070794884061 | 8.01061049094203 | 5.77160040366619 | 2.07830043402553e-08 | 2.77826568029933e-07 | 8.82883127830505 |
| MAP2K1 | -0.626201618 | 9.57466479713768 | -5.764311569 | 2.16018504195157e-08 | 2.86470011060696e-07 | 8.79170281443041 |
| SOSTDC1 | -1.034687884 | 6.46205082637681 | -5.760599853 | 2.20308486530125e-08 | 2.91176805524217e-07 | 8.77280992277947 |
| GABRA5 | -0.618826004 | 6.90620509380435 | -5.753578169 | 2.28652694048483e-08 | 3.01051133846089e-07 | 8.73709518927821 |
| SCN3B | -0.847224006 | 9.84881639355072 | -5.746773077 | 2.37033412594617e-08 | 3.1026187826314e-07 | 8.70251477247089 |
| FCGRT | 0.652291430411169 | 7.07437382706522 | 5.74665431945151 | 2.37182295869704e-08 | 3.1026187826314e-07 | 8.70191158484059 |
| SERPINF1 | -0.796409607 | 7.5411908 | -5.743945304 | 2.40603403960807e-08 | 3.13567057039256e-07 | 8.6881547595883 |
| RPP40 | -0.629269151 | 7.35142338057971 | -5.734661298 | 2.52696997048455e-08 | 3.27667404740621e-07 | 8.64104774302301 |
| TRAPPC1 | -0.737096138 | 7.2834909348913 | -5.731164595 | 2.57403968777861e-08 | 3.33002396742536e-07 | 8.6233210203738 |
| SLC17A7 | -0.607588478 | 11.0189161700725 | -5.728693969 | 2.60781178529078e-08 | 3.36734297100669e-07 | 8.61080118369322 |
| SLC16A9 | 0.825114317771705 | 7.80252722789855 | 5.71826971366974 | 2.75512794672223e-08 | 3.53478126229514e-07 | 8.55802331628822 |
| RNF25 | -0.67235791 | 5.70612893221015 | -5.689788655 | 3.20024548135015e-08 | 4.03570525044722e-07 | 8.41421053486308 |
| SNX10 | -0.726104291 | 9.17642321007246 | -5.688906256 | 3.21510065351497e-08 | 4.05079572904226e-07 | 8.40976398478492 |
| RGS1 | 1.10773133365266 | 6.17110381021739 | 5.68567721915648 | 3.27003693520821e-08 | 4.10893619555777e-07 | 8.39349698917487 |
| KCNV1 | -0.615141858 | 6.86771508086956 | -5.684474748 | 3.29072786980065e-08 | 4.12384959054642e-07 | 8.38744113476404 |
| FOXF1 | 0.658092385440874 | 6.47025883442029 | 5.68372619939223 | 3.30367256191528e-08 | 4.13637502997661e-07 | 8.38367182743524 |
| AKR1C3 | 0.654038623946056 | 8.32371619634058 | 5.67908388529858 | 3.38506841480818e-08 | 4.2307321195058e-07 | 8.36030426009757 |
| GJA4 | 0.705948426380469 | 6.53091646771739 | 5.67168009740787 | 3.51894517393588e-08 | 4.36312101376836e-07 | 8.32306776222858 |
| SLITRK4 | -0.599516075 | 8.77189652148551 | -5.670161131 | 3.54704283468222e-08 | 4.39400898151491e-07 | 8.31543304311604 |
| MT1H | 0.726004206561337 | 9.58525241257246 | 5.66843213484247 | 3.57929184782362e-08 | 4.41445994564914e-07 | 8.3067446319057 |
| CACNG3 | -0.628985242 | 8.09963224807971 | -5.667734079 | 3.59239275378223e-08 | 4.42672439246821e-07 | 8.30323741121824 |
| GPRASP1 | -0.615214879 | 9.74376356119565 | -5.65430525 | 3.85371627464353e-08 | 4.71147892932225e-07 | 8.23583391794904 |
| HACL1 | -0.607164684 | 7.01440189797101 | -5.653932288 | 3.86123241789448e-08 | 4.71655594042982e-07 | 8.23396370947012 |
| C21orf91 | 0.585945540953781 | 7.70513557028985 | 5.65351213838046 | 3.86971661503257e-08 | 4.72280557812026e-07 | 8.23185700102123 |
| DMXL2 | -0.626482582 | 7.52481275115942 | -5.640113795 | 4.1500111176049e-08 | 5.00392140173461e-07 | 8.16474023269013 |
| RARRES2 | 0.800990405734272 | 7.46029225862319 | 5.62887957756426 | 4.4002031109616e-08 | 5.25070147415964e-07 | 8.10856163491165 |
| VIM | 0.644688568669755 | 9.3241900640942 | 5.61432323951306 | 4.74633402604107e-08 | 5.60076126798412e-07 | 8.03590248436177 |
| RFWD3 | -0.700024995 | 5.15985731481884 | -5.607668414 | 4.91328280487695e-08 | 5.76078300775832e-07 | 8.00273417514748 |
| TCF7L1 | 0.59002659523845 | 5.61331876130435 | 5.59858379163365 | 5.15045436115874e-08 | 6.00372581101655e-07 | 7.957506026 |
| CARD6 | 0.717310392598979 | 5.31291852362319 | 5.59816293012005 | 5.16170796622672e-08 | 6.0042033461143e-07 | 7.95541216385244 |
| MXRA8 | 0.585422342606867 | 6.67075387724638 | 5.57843531540741 | 5.71696473445357e-08 | 6.52841990808162e-07 | 7.85740437836446 |
| TCAP | 0.668656008813814 | 6.02269905286232 | 5.57362044299418 | 5.86107931437818e-08 | 6.68210692890449e-07 | 7.83352570412116 |
| KDELC2 | 0.671064735432457 | 6.35778805387681 | 5.57324369543076 | 5.87250390762557e-08 | 6.68969311914162e-07 | 7.83165797168276 |
| KIF1C | 0.590093782931815 | 7.61968360630435 | 5.5546378529013 | 6.46452108787929e-08 | 7.31064348510737e-07 | 7.73954453012723 |
| CYC1 | -0.60738719 | 9.45989871275362 | -5.553479895 | 6.50322801876925e-08 | 7.34849045182927e-07 | 7.73381986466154 |
| PRKAG1 | -0.664058024 | 6.79591594880435 | -5.552233786 | 6.5451337490992e-08 | 7.38988812911579e-07 | 7.72766046155355 |
| KCNIP1 | -0.609886763 | 5.97318786981884 | -5.543540874 | 6.8448747820414e-08 | 7.66658778502928e-07 | 7.68472293655859 |
| GDA | -0.691164158 | 8.65645179141304 | -5.538022405 | 7.04205024118142e-08 | 7.87485410941684e-07 | 7.65749307040591 |
| VSNL1 | -0.731277757 | 11.7754398572826 | -5.533454821 | 7.20942447506607e-08 | 8.03000471913038e-07 | 7.63497157265151 |
| MSH2 | -0.786081953 | 5.90065649061594 | -5.530480454 | 7.32049285899353e-08 | 8.13433212057577e-07 | 7.62031378246615 |
| GRIA1 | -0.646890679 | 8.13246619235507 | -5.517188538 | 7.8375346650904e-08 | 8.63360161889731e-07 | 7.55488782549322 |
| NELL1 | -0.747311756 | 7.1100890898913 | -5.500188687 | 8.55084627998927e-08 | 9.29523390575888e-07 | 7.4713943844199 |
| CNR1 | -0.667339555 | 7.53131560902174 | -5.492283465 | 8.90370513892322e-08 | 9.61175189862358e-07 | 7.43263889882733 |
| OCIAD2 | -0.628398368 | 8.5080947415942 | -5.490300985 | 8.99439459665755e-08 | 9.69472678162405e-07 | 7.42292677641179 |
| TSPO | 0.717164769643512 | 7.03019618931159 | 5.48463863241845 | 9.25840103981212e-08 | 9.96397220117309e-07 | 7.39520253981694 |
| EMP3 | 0.71137260119091 | 6.45483892336957 | 5.47959619667416 | 9.49984126592038e-08 | 1.0210810403508e-06 | 7.3705329084492 |
| WDR46 | -0.720219817 | 6.33326478315217 | -5.473369829 | 9.80643500866932e-08 | 1.05295281873331e-06 | 7.3400961637589 |
| RASL12 | 0.802784649898522 | 6.93225582557971 | 5.47070633320271 | 9.94050994276363e-08 | 1.06571690311448e-06 | 7.32708452273956 |
| ITGA5 | 0.592869741781906 | 6.39584692228261 | 5.45780850943036 | 1.0615404804663e-07 | 1.13201385228737e-06 | 7.26414847941911 |
| TCEAL7 | -0.609882299 | 9.12635969057971 | -5.437319345 | 1.17801645990089e-07 | 1.24112132360557e-06 | 7.16441580480398 |
| TAC1 | -0.977085712 | 7.04648182992754 | -5.435805067 | 1.18710176056047e-07 | 1.24975435347894e-06 | 7.15705692975971 |
| HAS1 | -0.646250689 | 6.32875495851449 | -5.433271673 | 1.20245412920312e-07 | 1.26496731086387e-06 | 7.14474919609828 |
| PPM1J | -0.625404561 | 6.04846211699275 | -5.424097618 | 1.25968165695585e-07 | 1.32120537587822e-06 | 7.10021852382474 |
| GRTP1 | 0.628442854164788 | 5.18634029884058 | 5.41088704455096 | 1.34676703278338e-07 | 1.40518706106557e-06 | 7.03620141205774 |
| IFITM2 | 0.712990116385194 | 10.08283235 | 5.4089078385543 | 1.36030849734707e-07 | 1.41445157313323e-06 | 7.02662123924605 |
| FGFR3 | 0.672366562914457 | 10.4160538344565 | 5.40885595757694 | 1.36066523508081e-07 | 1.41445157313323e-06 | 7.0263701520215 |
| GPR22 | -0.630269638 | 6.39525924615942 | -5.402527097 | 1.40487231710851e-07 | 1.45284104003043e-06 | 6.99575510658619 |
| OR2L13 | -0.781243548 | 5.53709904384058 | -5.397061495 | 1.44417086384638e-07 | 1.4879947115149e-06 | 6.96933928305555 |
| PRR3 | -0.708056994 | 5.57321495594203 | -5.383905982 | 1.54319468787454e-07 | 1.57727544519422e-06 | 6.90584602190034 |
| DNTTIP1 | -0.590568166 | 6.73695529286232 | -5.383109255 | 1.54939924757262e-07 | 1.58246363064172e-06 | 6.9020047571364 |
| LRRTM1 | -0.672676456 | 7.72469179717391 | -5.382246881 | 1.5561423663032e-07 | 1.58810313005675e-06 | 6.89784750883988 |
| COG7 | -0.647242786 | 6.22362070394928 | -5.377137171 | 1.59668571509001e-07 | 1.61896773555367e-06 | 6.87322617758189 |
| NAP1L5 | -0.60237812 | 10.7982877993841 | -5.361192442 | 1.7299122747495e-07 | 1.73523317802663e-06 | 6.79651774617692 |
| CGREF1 | -0.644465961 | 6.61238052927536 | -5.358806752 | 1.75075321690797e-07 | 1.75237775593865e-06 | 6.78505632397215 |
| SCG5 | -0.674238239 | 11.1036676919928 | -5.353434198 | 1.79858434676138e-07 | 1.79385123005938e-06 | 6.759260462 |
| NRIP3 | -0.622392179 | 9.79401865630435 | -5.350791368 | 1.82257677765516e-07 | 1.81519844837062e-06 | 6.74657884321698 |
| TRIM17 | -0.649529441 | 5.61185168442029 | -5.348737311 | 1.84143868620068e-07 | 1.8287885762459e-06 | 6.73672596148321 |
| CPNE6 | -0.615708813 | 8.54642107815217 | -5.345874308 | 1.86804574301442e-07 | 1.85137379366252e-06 | 6.72299786063698 |
| ZFP36 | 0.717523538116621 | 8.25998189681159 | 5.34154860340954 | 1.90895631666419e-07 | 1.88516157947761e-06 | 6.70226742576351 |
| INHBB | 0.687274449247068 | 6.88696258547101 | 5.33629571127947 | 1.95980737544322e-07 | 1.92858798777827e-06 | 6.67711188513646 |
| CITED1 | -0.603213089 | 6.69010927865942 | -5.332681613 | 1.99555574519039e-07 | 1.96101459108653e-06 | 6.65981603470928 |
| LFNG | 0.591880744560707 | 6.01118046231884 | 5.32119751894619 | 2.11340491731891e-07 | 2.05950501428513e-06 | 6.60492029541096 |
| NRGN | -0.616691601 | 10.7709476203261 | -5.309904247 | 2.23587686440824e-07 | 2.16531086116e-06 | 6.55103063933598 |
| NEFL | -0.705205207 | 10.8847606592029 | -5.299085899 | 2.35964846597179e-07 | 2.27301300191883e-06 | 6.49949470122287 |
| RIT2 | -0.631507094 | 7.58715095007246 | -5.297990366 | 2.37254721890399e-07 | 2.2819087551914e-06 | 6.49428062780059 |
| SPR | 0.615935848573008 | 7.06050565481884 | 5.29588665103445 | 2.39750854594211e-07 | 2.30275769450316e-06 | 6.48427068465869 |
| NPTX2 | -0.683271446 | 8.64696647007246 | -5.292443318 | 2.43891632202495e-07 | 2.33932445853323e-06 | 6.46789353560043 |
| CHAF1B | -0.621460335 | 5.58106486663043 | -5.287987265 | 2.49353472496514e-07 | 2.38193715587099e-06 | 6.44671258936811 |
| SLC7A14 | -0.627748532 | 7.61631587206522 | -5.28218411 | 2.56644815575957e-07 | 2.44085508482927e-06 | 6.4191502918845 |
| PTRH2 | -0.63510356 | 8.32434544376812 | -5.274080541 | 2.67173882152347e-07 | 2.53147253339348e-06 | 6.38070343093534 |
| ERC2 | -0.659362629 | 9.12886473913044 | -5.26854528 | 2.74605768177398e-07 | 2.59487647382187e-06 | 6.35446944556708 |
| KCNJ16 | 0.618939845342021 | 7.66410820463768 | 5.25929428722677 | 2.87476868957972e-07 | 2.69549765622447e-06 | 6.31067526719475 |
| C14orf79 | -0.659777471 | 6.44677905717391 | -5.252182321 | 2.97768240529851e-07 | 2.77448773219275e-06 | 6.2770500224794 |
| ESAM | 0.751776916733794 | 6.27919714811594 | 5.23406011261458 | 3.25635150320528e-07 | 3.00617624288662e-06 | 6.1915368993818 |
| EPDR1 | -0.652499667 | 9.6856949682971 | -5.226556718 | 3.37900025111942e-07 | 3.10509308790613e-06 | 6.15620156198821 |
| PTPRT | -0.717207901 | 7.79129289018116 | -5.222005809 | 3.45556004315624e-07 | 3.15450990914262e-06 | 6.1347904537077 |
| PPP1R13L | 0.680072151544246 | 6.09433431036232 | 5.21390852737229 | 3.59596259713688e-07 | 3.25330216126777e-06 | 6.09673219179871 |
| SYT1 | -0.794112827 | 10.5080114814493 | -5.196582686 | 3.91522326956293e-07 | 3.52169184792053e-06 | 6.01546135900131 |
| PCP4 | -0.707182162 | 9.92450638347826 | -5.184388017 | 4.15624653871749e-07 | 3.69906129777423e-06 | 5.9583927381424 |
| NKX2-2 | 0.680856278814344 | 6.64920284423913 | 5.18315616767587 | 4.18137906019778e-07 | 3.7158097947499e-06 | 5.95263405795851 |
| SHF | -0.608235117 | 6.0045895282971 | -5.179045132 | 4.26632488419205e-07 | 3.78410334288584e-06 | 5.93342383440882 |
| SUSD1 | -0.757872386 | 6.27418895362319 | -5.178594533 | 4.27573671343042e-07 | 3.78765988202368e-06 | 5.93131901725466 |
| PPP1CA | -0.593985751 | 7.41064099 | -5.176272216 | 4.32456403354277e-07 | 3.82366717795525e-06 | 5.92047351343612 |
| ZFPM2 | -0.6345337 | 7.70723524771739 | -5.171033015 | 4.43671407176587e-07 | 3.91049914697504e-06 | 5.89602050699456 |
| LATS2 | 0.621488623324049 | 7.00879345 | 5.16261143713277 | 4.62292113626772e-07 | 4.03912967480877e-06 | 5.85675706800182 |
| TGFB1I1 | 0.866604599699244 | 6.80707415884058 | 5.10939093692885 | 5.98739494786867e-07 | 5.11646796794408e-06 | 5.60985147053857 |
| HSPB2 | 0.586425144725276 | 6.59500521771739 | 5.10914201867018 | 5.99461306570639e-07 | 5.11951638370284e-06 | 5.6087016313041 |
| SNCB | -0.777530785 | 8.07246826130435 | -5.108683995 | 6.0079168406383e-07 | 5.12688958353633e-06 | 5.6065859847616 |
| MAP7D2 | -0.686304848 | 9.47258496800725 | -5.090245642 | 6.56789411253877e-07 | 5.55833308027345e-06 | 5.52154809434546 |
| NELL2 | -0.779127232 | 11.4692185718841 | -5.087559194 | 6.65359059622921e-07 | 5.62068077897122e-06 | 5.50917941211942 |
| PGM2L1 | -0.628378446 | 8.85049596155797 | -5.085739193 | 6.71226229036671e-07 | 5.6600152794836e-06 | 5.50080301467982 |
| TUBB6 | 0.604707246308954 | 6.50840592735507 | 5.0749394835891 | 7.07086367436561e-07 | 5.93030629818355e-06 | 5.4511493869623 |
| CCNA1 | -0.671598126 | 6.77283925336957 | -5.067715193 | 7.32102998625617e-07 | 6.11088116055181e-06 | 5.41798325105993 |
| SLC30A3 | -0.777063198 | 7.93545995586957 | -5.064252539 | 7.44395512903527e-07 | 6.19137501627886e-06 | 5.40210037581456 |
| TMEM176A | 0.608001737567166 | 7.30834909880435 | 5.06320224209328 | 7.48163441881496e-07 | 6.21534119994326e-06 | 5.39728454408487 |
| GNG2 | -0.618286655 | 7.88941134518116 | -5.062579455 | 7.50406403424622e-07 | 6.23028359693515e-06 | 5.39442932263706 |
| NAP1L2 | -0.593548555 | 9.23176122753623 | -5.055443281 | 7.7657660233919e-07 | 6.42473964283331e-06 | 5.36173375130266 |
| RPA3 | -0.807018633 | 6.50290669050725 | -5.041013137 | 8.32223186669666e-07 | 6.83671104081355e-06 | 5.29573668603557 |
| RUVBL1 | -0.597278388 | 5.87831982362319 | -5.029624364 | 8.78848670455057e-07 | 7.19025373733446e-06 | 5.24376023107064 |
| GIMAP7 | 0.746865993651614 | 6.69157265065217 | 5.00747318259758 | 9.76883538586248e-07 | 7.90469582319385e-06 | 5.14294619263817 |
| ZNF566 | 0.588093031295542 | 6.11225147438406 | 5.00691419917919 | 9.79489495620003e-07 | 7.91664622310046e-06 | 5.14040695232097 |
| SLC32A1 | -0.667082593 | 7.34186493460145 | -4.997735419 | 1.02325514995018e-06 | 8.25135535810889e-06 | 5.09874518578957 |
| PRC1 | -0.627444223 | 6.71595236786232 | -4.990211716 | 1.06053491873026e-06 | 8.50794117011123e-06 | 5.0646432273041 |
| CORO1A | -0.711492173 | 7.51362327061594 | -4.990070887 | 1.06124510427772e-06 | 8.50877078175328e-06 | 5.06400531114127 |
| SYP | -0.648896286 | 8.54797090286232 | -4.986537453 | 1.07921511440185e-06 | 8.6331052762448e-06 | 5.04800482795192 |
| SNAP91 | -0.678066631 | 11.1671885004348 | -4.974362985 | 1.14341344871565e-06 | 9.07932434390686e-06 | 4.99294752856974 |
| TDRD10 | 0.586867655696936 | 6.02465180181159 | 4.97205493127922 | 1.15599397548787e-06 | 9.16883683159863e-06 | 4.98252235621848 |
| ANKRD36B | 0.652762409723952 | 9.89420392978261 | 4.96878524938655 | 1.1740454936811e-06 | 9.28051857829201e-06 | 4.96776055885593 |
| ZBBX | -0.637419576 | 6.45974541663043 | -4.93967891 | 1.34721989731531e-06 | 1.05131133111033e-05 | 4.83671067531267 |
| HPCA | -0.652929014 | 8.88195761293478 | -4.900987494 | 1.61603236161827e-06 | 1.24241347625948e-05 | 4.66350341173007 |
| GLRX | -0.619935135 | 8.68877504572464 | -4.887655404 | 1.72014174657898e-06 | 1.31418937274682e-05 | 4.60408542165937 |
| SERPINI1 | -0.671322834 | 10.4534322211232 | -4.871633662 | 1.85384293888316e-06 | 1.39440499895226e-05 | 4.53286031794317 |
| KLF4 | 0.641297038131869 | 6.71658493474638 | 4.87016825075888 | 1.86656100433384e-06 | 1.40186303399785e-05 | 4.526355612 |
| TMEM169 | -0.63341772 | 6.19464714695652 | -4.859987528 | 1.95727355081099e-06 | 1.46160495864415e-05 | 4.48121062813376 |
| LY6E | -0.628619912 | 8.28088239188406 | -4.85701534 | 1.98454969095616e-06 | 1.47868353890426e-05 | 4.46804588303195 |
| TXNIP | 0.644428530816027 | 8.77098641764493 | 4.84660632851776 | 2.08299849232071e-06 | 1.54467942135449e-05 | 4.42199462952371 |
| NEU1 | -0.595953337 | 6.7066092957971 | -4.828440887 | 2.26621321700035e-06 | 1.65975692388652e-05 | 4.34182718218799 |
| LRRC4 | -0.648897061 | 6.53527534623188 | -4.816599924 | 2.39391335990216e-06 | 1.74117463931058e-05 | 4.28970763773756 |
| LPAR4 | 0.593908912139438 | 5.59404111789855 | 4.78906286894194 | 2.71831328054464e-06 | 1.94881938308167e-05 | 4.16891804448827 |
| IRX3 | 0.660941517531945 | 5.44244259427536 | 4.78337518419723 | 2.79041257923972e-06 | 1.99134634089967e-05 | 4.14404238511184 |
| CDH12 | -0.673985679 | 7.1488301448913 | -4.773288526 | 2.92283620467336e-06 | 2.06962918890188e-05 | 4.09998894780525 |
| NPTXR | -0.587067573 | 8.49907778148551 | -4.767539984 | 3.0009969453019e-06 | 2.11721672864683e-05 | 4.07491746300931 |
| VSIG4 | 0.600433106869448 | 7.49510062155797 | 4.75760311673667 | 3.14088273754976e-06 | 2.20996480826193e-05 | 4.03163958239858 |
| PRMT6 | -0.604936953 | 6.05153654996377 | -4.746584107 | 3.30335387833286e-06 | 2.31037064518014e-05 | 3.98373824617011 |
| TBC1D19 | -0.701170632 | 5.87789260692029 | -4.735152791 | 3.48045715771201e-06 | 2.41855553630305e-05 | 3.93414422113158 |
| FCRLB | -0.638839657 | 6.26081476025362 | -4.724836219 | 3.64812026161585e-06 | 2.51265178922588e-05 | 3.88947366863617 |
| SPTBN2 | -0.631752915 | 6.87144003050725 | -4.713938782 | 3.83367030610495e-06 | 2.62497845227099e-05 | 3.8423779678729 |
| P2RY14 | 0.675667646418316 | 6.09393519634058 | 4.70910800944792 | 3.91880283571126e-06 | 2.67673512738329e-05 | 3.82153030831156 |
| ZNF222 | -0.652209875 | 5.37993712224638 | -4.688042495 | 4.3118083242768e-06 | 2.9195793399968e-05 | 3.73083284549375 |
| NECAB1 | -0.69212087 | 6.69730865771739 | -4.677851005 | 4.5153141254455e-06 | 3.04268380495542e-05 | 3.68707783894462 |
| C15orf52 | 0.590758801321844 | 6.94935833195652 | 4.67708770982466 | 4.53092259384681e-06 | 3.04880650352754e-05 | 3.68380406367837 |
| HIST1H1C | 0.601252370504758 | 7.35078068112319 | 4.67245323281021 | 4.62681362624503e-06 | 3.10587876882882e-05 | 3.66393655005126 |
| SEMA5B | -0.618892257 | 7.11693591061594 | -4.655278641 | 4.99949944915991e-06 | 3.31622124811675e-05 | 3.59045750409908 |
| ST6GALNAC2 | 0.610494268332721 | 4.92168756978261 | 4.63645176743552 | 5.44122443551659e-06 | 3.58563394075419e-05 | 3.51017507521341 |
| TRIM56 | 0.712682602313479 | 6.57710578478261 | 4.62568418194241 | 5.71049953209773e-06 | 3.73499696542008e-05 | 3.46438453285205 |
| FGF13 | -0.591630408 | 8.86332597083333 | -4.625049252 | 5.72677188357942e-06 | 3.74214921358034e-05 | 3.46168725569765 |
| AQP11 | -0.636816837 | 6.03749846891304 | -4.601182611 | 6.37185710956793e-06 | 4.11762913582816e-05 | 3.36052829280042 |
| UBE2M | -0.72549797 | 7.54788088398551 | -4.59311743 | 6.60524000585373e-06 | 4.24497161329454e-05 | 3.32644538038079 |
| CAP2 | -0.585118573 | 10.8587896920652 | -4.572883861 | 7.22748807830929e-06 | 4.60059306954749e-05 | 3.24116557015233 |
| PCDH8 | -0.688140905 | 8.26784618188406 | -4.514406887 | 9.35891808778682e-06 | 5.78660089704738e-05 | 2.99651962759535 |
| NRSN1 | -0.598324867 | 8.56978400865942 | -4.513038259 | 9.41540405437118e-06 | 5.81383580160489e-05 | 2.99082628366577 |
| DACH2 | -0.618863915 | 7.54333243706522 | -4.504227638 | 9.78695161324986e-06 | 6.02160952498426e-05 | 2.95421071556557 |
| CXCL1 | 0.621483380348598 | 6.17707870307971 | 4.46284112287705 | 1.17288997006842e-05 | 7.06808596917467e-05 | 2.78304204264382 |
| GALNTL5 | -0.718549167 | 5.34784101434783 | -4.427692034 | 1.36637983298853e-05 | 8.08470227763635e-05 | 2.63874457707367 |
| CCL2 | 0.743075446133867 | 5.45067736905797 | 4.40392603389379 | 1.51417382541298e-05 | 8.86492638170903e-05 | 2.54173891081007 |
| HIGD1B | 0.889612900730849 | 7.16197224565217 | 4.3766344113755 | 1.70279990906834e-05 | 9.79826143818847e-05 | 2.43090236964304 |
| IFITM3 | 0.663456913117926 | 9.89599075894928 | 4.37537366717718 | 1.71203635225204e-05 | 9.84736905973352e-05 | 2.42579672579268 |
| F3 | 0.603082690908042 | 8.52944634253623 | 4.3717844828229 | 1.73859493989072e-05 | 9.98783975505431e-05 | 2.41126859279374 |
| TYRP1 | -0.703397588 | 5.35132764003623 | -4.347751171 | 1.92687171916866e-05 | 0.000108647053147978 | 2.31425524807477 |
| MUM1L1 | -0.627046917 | 6.98941485184783 | -4.269407921 | 2.68574954334773e-05 | 0.000146147713800408 | 2.00125669471853 |
| ABT1 | -0.601823801 | 5.37989891373188 | -4.257559198 | 2.82290277687667e-05 | 0.000152663191824688 | 1.95435210095386 |
| SYNPR | -0.61298177 | 9.66269853351449 | -4.19112991 | 3.72463835165981e-05 | 0.000196060824344315 | 1.69350403626657 |
| TMEM200A | -0.631248061 | 6.58243504902174 | -4.18420148 | 3.83313383190853e-05 | 0.00020079206471742 | 1.66650592122497 |
| PLA2G4A | -0.586596406 | 5.19817650619565 | -4.140130092 | 4.5971770789248e-05 | 0.000235967108996202 | 1.49569434140044 |
| CDH13 | -0.693072785 | 7.04646655601449 | -4.104079856 | 5.32805195777015e-05 | 0.000269147235604506 | 1.35715906034394 |
| PLA1A | 0.683728703335084 | 5.67334369452899 | 4.07843526629475 | 5.91397421585662e-05 | 0.000294725166974321 | 1.25926375658957 |
| TRHDE | -0.596190317 | 6.8120504792029 | -4.003415822 | 8.00079760525303e-05 | 0.000382006077012132 | 0.976013650664435 |
| PVALB | -0.786604951 | 6.63337815376812 | -3.6889758 | 0.000270431349669934 | 0.00110884760714079 | -0.159886655 |
| PDYN | -0.592869434 | 5.852619795 | -3.406399357 | 0.000754663018427227 | 0.00271767835321135 | -1.108670583 |
| TGFBI | 0.66151239958831 | 5.87629474496377 | 3.31957211392175 | 0.00102093767625555 | 0.00352973595516064 | -1.386269295 |
| RPS4Y1 | -1.008573189 | 7.6372117423913 | -3.291282214 | 0.00112506838282749 | 0.00384425778079675 | -1.47528738 |
