# Supplementary material for: Deciphering Alzheimer’s disease transcriptomics: exploration and validation of core genes in tau and Aβ pathological models toward novel therapeutic targets
Source: Front Aging Neurosci. 2025 Oct 10;17:1621153. doi: 10.3389/fnagi.2025.1621153 (PMC12549628; doi:10.3389/fnagi.2025.1621153)
Supplement: Supplementary file 2 [file Data_Sheet_1.PDF]

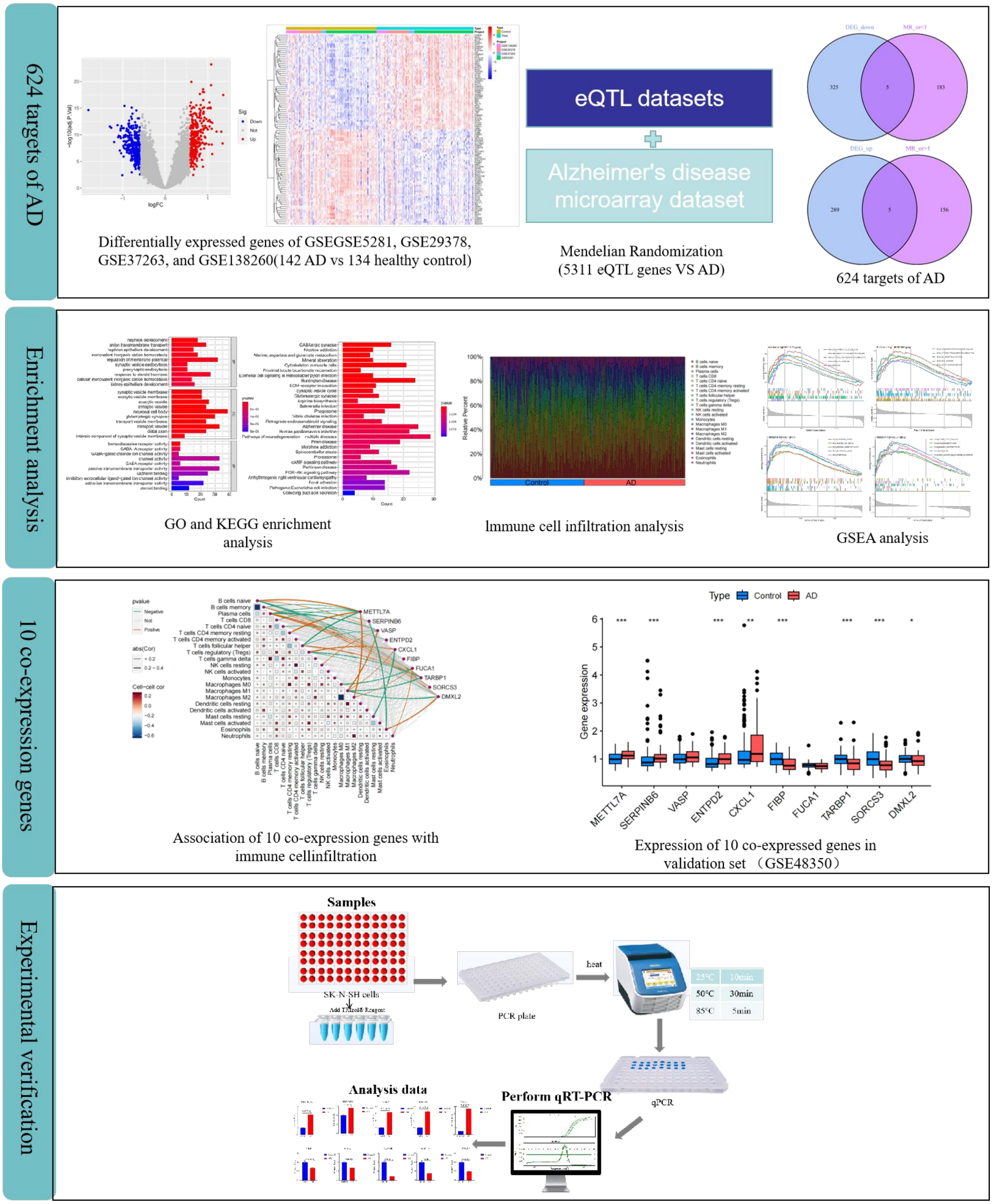
624 targets of AD
Differentially expressed genes of GSEGSE5281, GSE29378, GSE37263, and GSE138260(142 AD vs 134 healthy control)
eQTL datasets
Alzheimer's disease microarray dataset
Mendelian Randomization (5311 eQTL genes VS AD)
DEG_down
MR_or<1
325
5
183
DEG_up
MR_or>1
289
5
156
624 targets of AD
Enrichment analysis
GO and KEGG enrichment analysis
Immune cell infiltration analysis
Control
AD
GSEA analysis
10 co-expression genes
METTL7A
SERPINB6
VASP
ENTPD2
CXCL1
FIBP
FUCA1
TARBP1
SORCS3
DMXL2
Association of 10 co-expression genes with immune cellinfiltration
Type
Control
AD
Gene expression
Expression of 10 co-expressed genes in validation set （GSE48350）
Experimental verification
Samples
SK-N-SH cells
PCR plate
heat
25°C
10min
50°C
30min
85°C
5min
qPCR
Perform qRT-PCR
Analysis data
