# Supplementary material for: Deciphering Alzheimer’s disease transcriptomics: exploration and validation of core genes in tau and Aβ pathological models toward novel therapeutic targets
Source: Front Aging Neurosci. 2025 Oct 10;17:1621153. doi: 10.3389/fnagi.2025.1621153 (PMC12549628; doi:10.3389/fnagi.2025.1621153)

exposure	nsnp	method	pval	OR(95% CI)
CXCL1	7	Weighted median	**0.033**	1.052 (1.004 to 1.102)
	7	Inverse variance weighted	**0.004**	1.060 (1.019 to 1.104)
DMXL2	6	Weighted median	**0.009**	0.946 (0.907 to 0.986)
	6	Inverse variance weighted	**0.011**	0.950 (0.913 to 0.988)
ENTPD2	3	Weighted median	**0.010**	1.057 (1.013 to 1.102)
	3	Inverse variance weighted	**0.007**	1.056 (1.015 to 1.099)
FIBP	5	Weighted median	**0.004**	0.940 (0.902 to 0.981)
	5	Inverse variance weighted	**<0.001**	0.934 (0.897 to 0.973)
FUCA1	6	Weighted median	**0.006**	0.946 (0.909 to 0.984)
	6	Inverse variance weighted	**0.007**	0.943 (0.904 to 0.984)
METTL7A	3	Weighted median	**0.002**	1.067 (1.024 to 1.111)
	3	Inverse variance weighted	**0.001**	1.067 (1.026 to 1.110)
SERPINB6	4	Weighted median	**0.017**	1.026 (1.005 to 1.047)
	4	Inverse variance weighted	**0.033**	1.022 (1.002 to 1.043)
SORCS3	5	Weighted median	0.153	0.941 (0.865 to 1.023)
	5	Inverse variance weighted	**0.016**	0.909 (0.840 to 0.982)
TARBP1	4	Weighted median	0.145	0.937 (0.859 to 1.023)
	4	Inverse variance weighted	**0.033**	0.920 (0.853 to 0.993)
VASP	5	Weighted median	0.078	1.037 (0.996 to 1.079)
	5	Inverse variance weighted	**0.040**	1.046 (1.002 to 1.092)

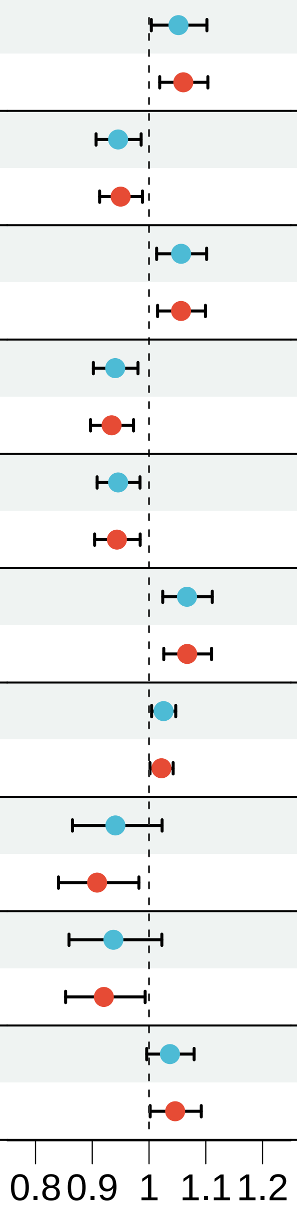

Supplement: Supplementary file 3 [file Data_Sheet_2.ZIP › Figure/Figure8.pdf]
