# Supplementary figures and images for: Deciphering Alzheimer’s disease transcriptomics: exploration and validation of core genes in tau and Aβ pathological models toward novel therapeutic targets
Source: Front Aging Neurosci. 2025 Oct 10;17:1621153. doi: 10.3389/fnagi.2025.1621153 (PMC12549628; doi:10.3389/fnagi.2025.1621153)

# Enriched in high CXCL1 group

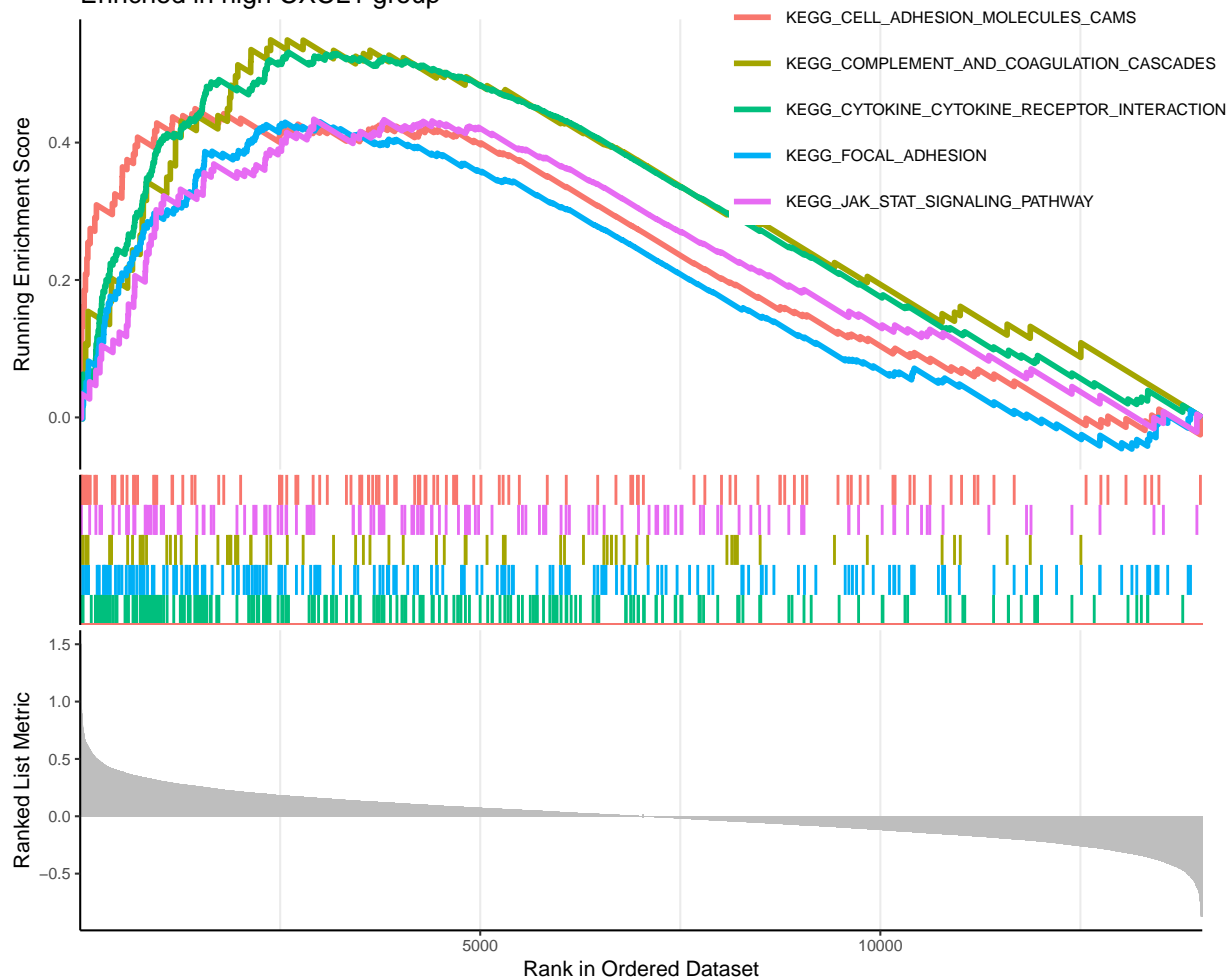

Supplement: Supplementary file 3 [file Data_Sheet_2.ZIP › Figure/CXCL1-GSEA.highExp.pdf]

# Enriched in high DMXL2 group

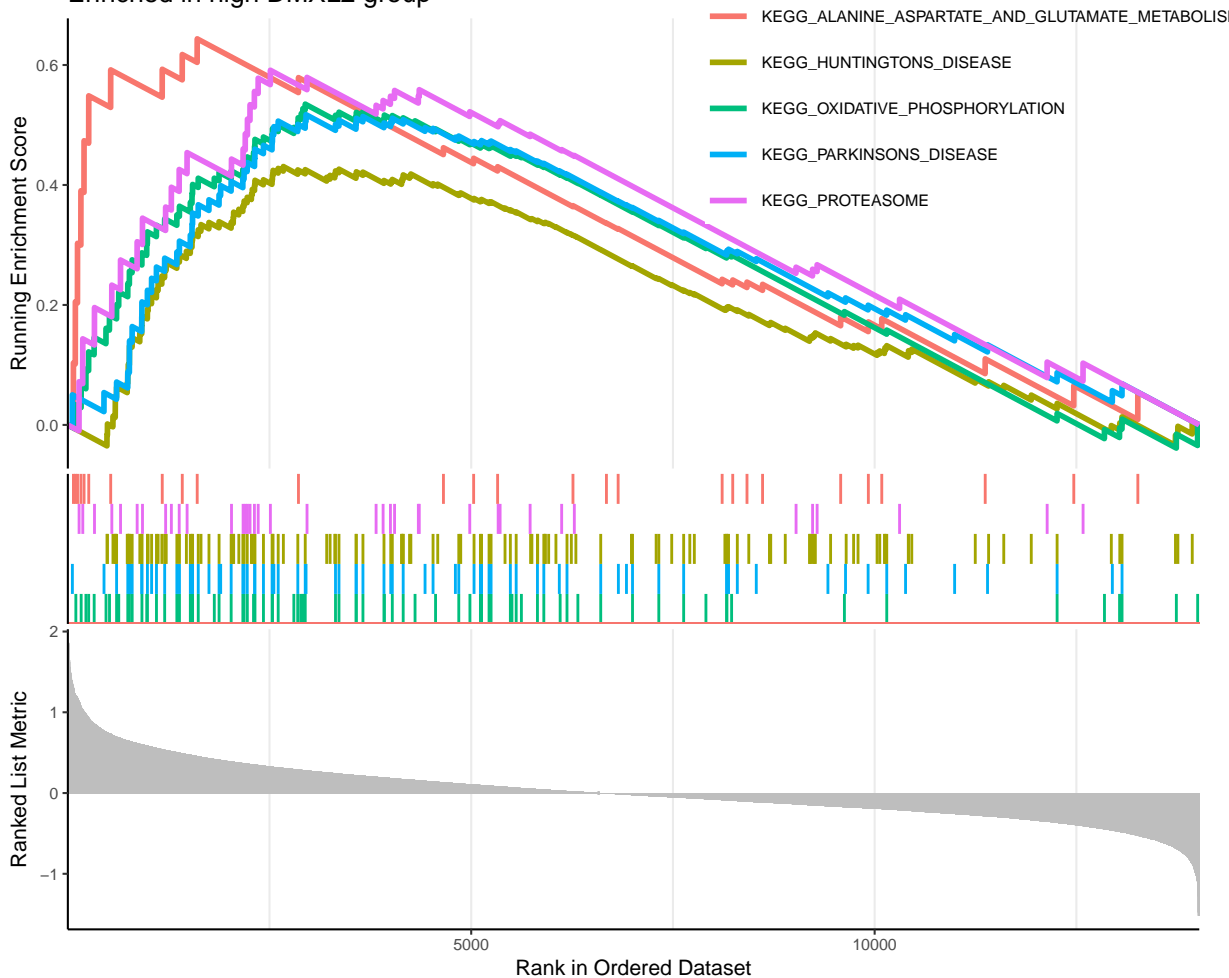

Supplement: Supplementary file 3 [file Data_Sheet_2.ZIP › Figure/DMXL2-GSEA.highExp.pdf]

# Enriched in high FIBP group

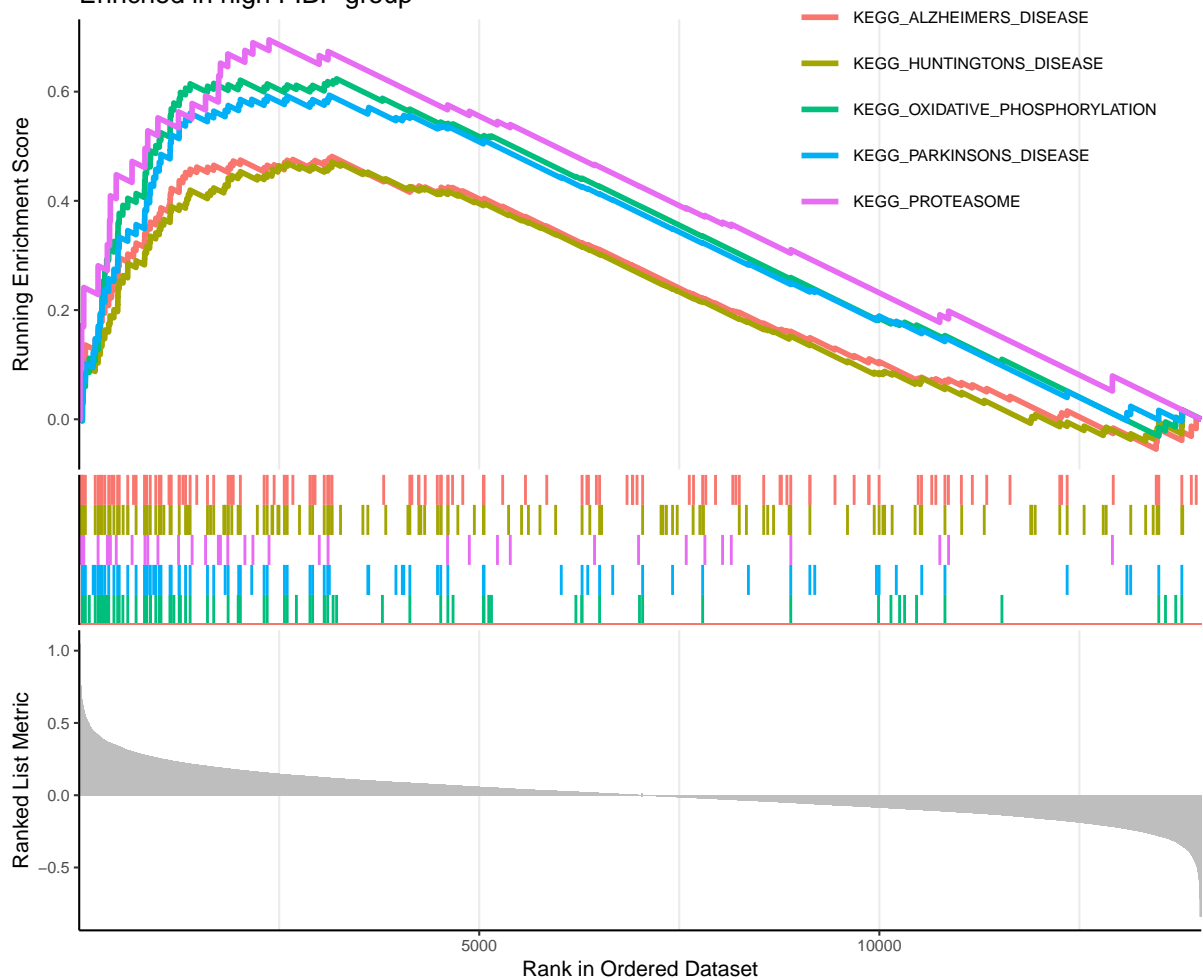

Supplement: Supplementary file 3 [file Data_Sheet_2.ZIP › Figure/FIBP-GSEA.highExp.pdf]

# Before batch correction

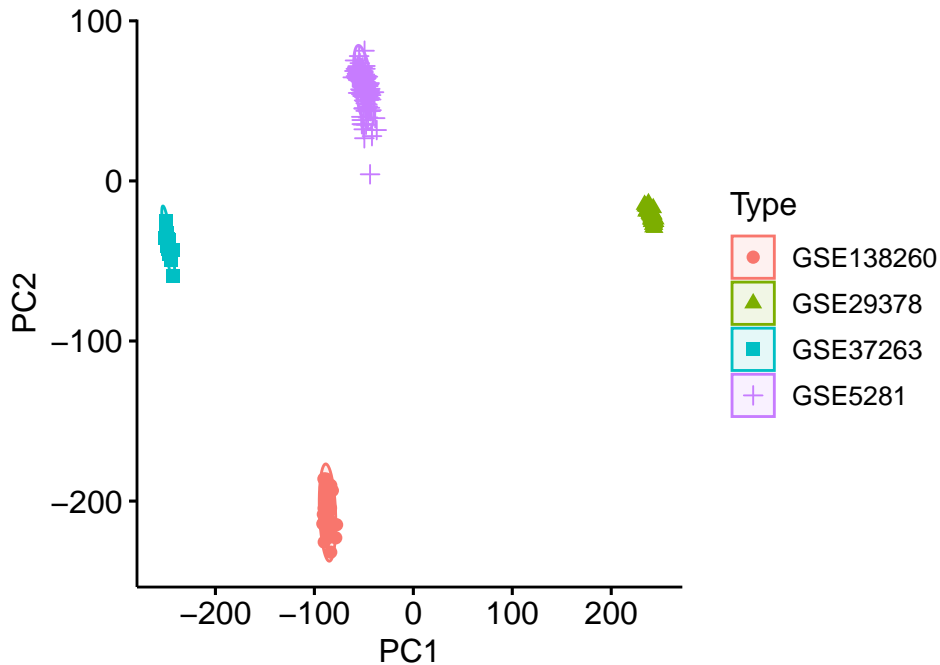

Supplement: Supplementary file 3 [file Data_Sheet_2.ZIP › Figure/Figure1.pdf]

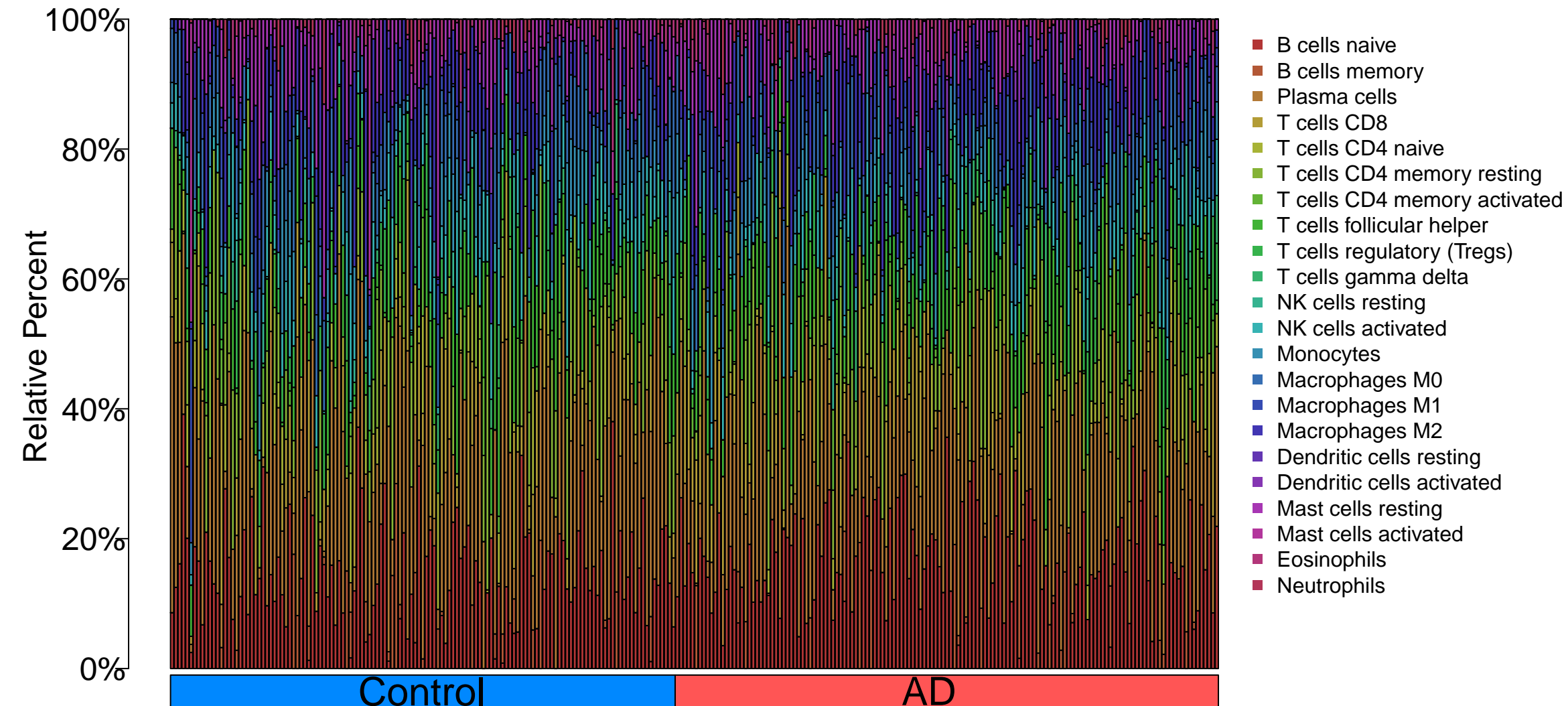

Supplement: Supplementary file 3 [file Data_Sheet_2.ZIP › Figure/Figure10A.pdf]

Type Control AD

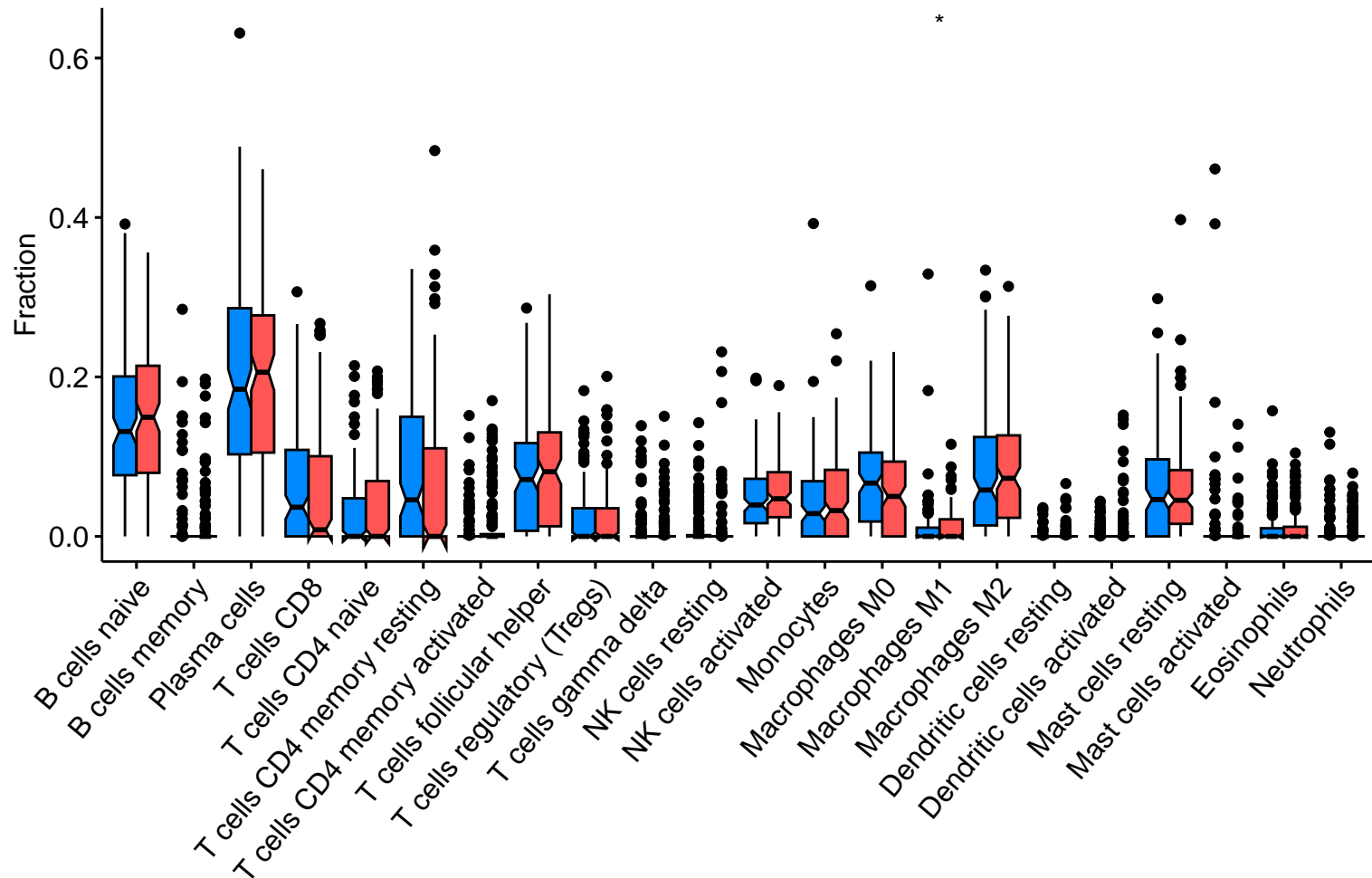

Supplement: Supplementary file 3 [file Data_Sheet_2.ZIP › Figure/Figure10B.pdf]

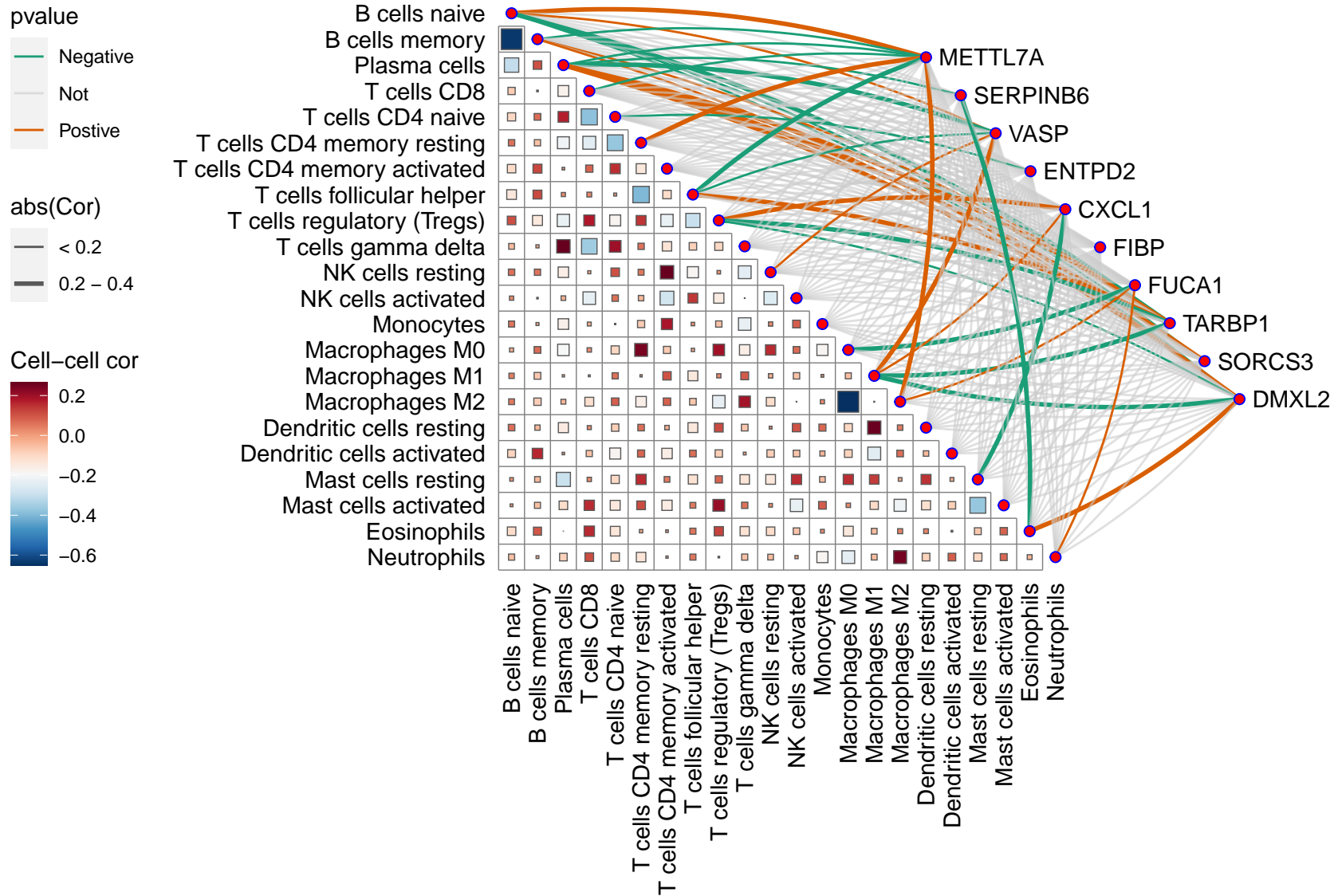

Supplement: Supplementary file 3 [file Data_Sheet_2.ZIP › Figure/Figure10C.pdf]

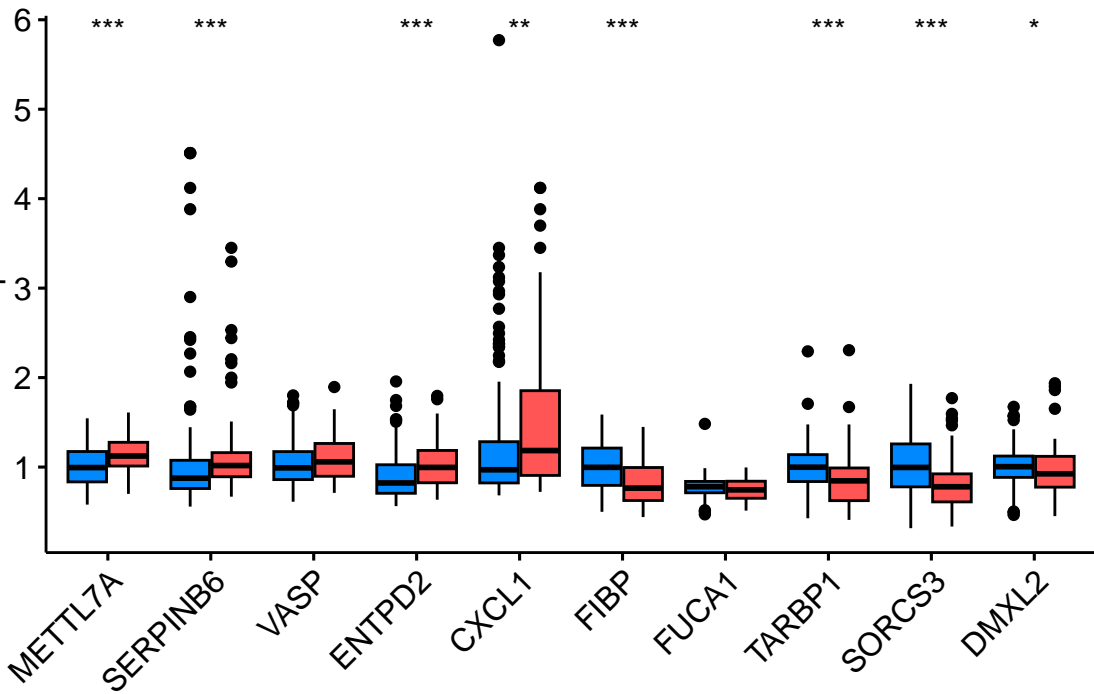

Supplement: Supplementary file 3 [file Data_Sheet_2.ZIP › Figure/Figure12.pdf]

## After batch correction

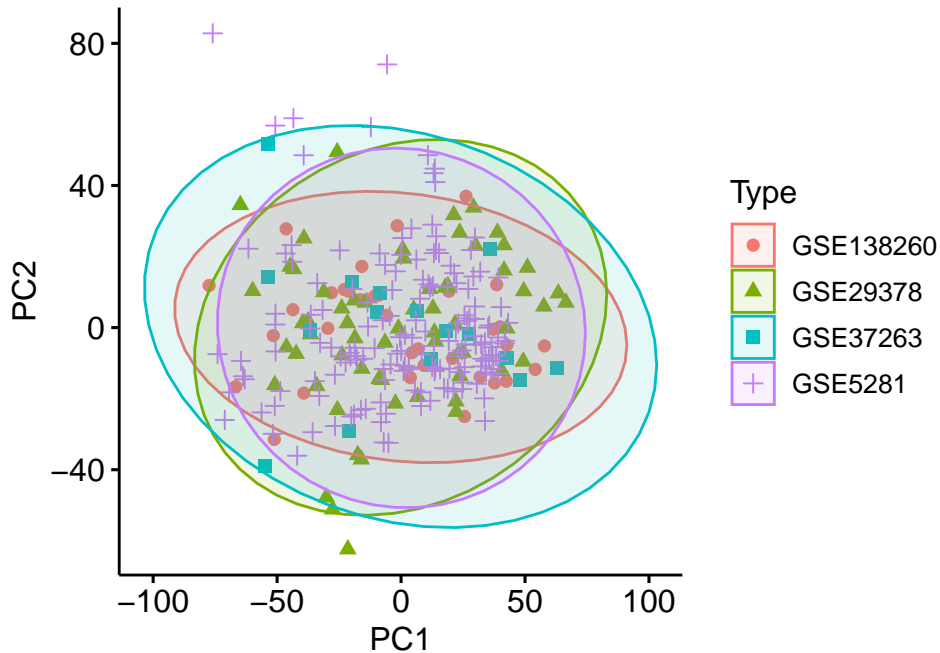

Supplement: Supplementary file 3 [file Data_Sheet_2.ZIP › Figure/Figure2.pdf]

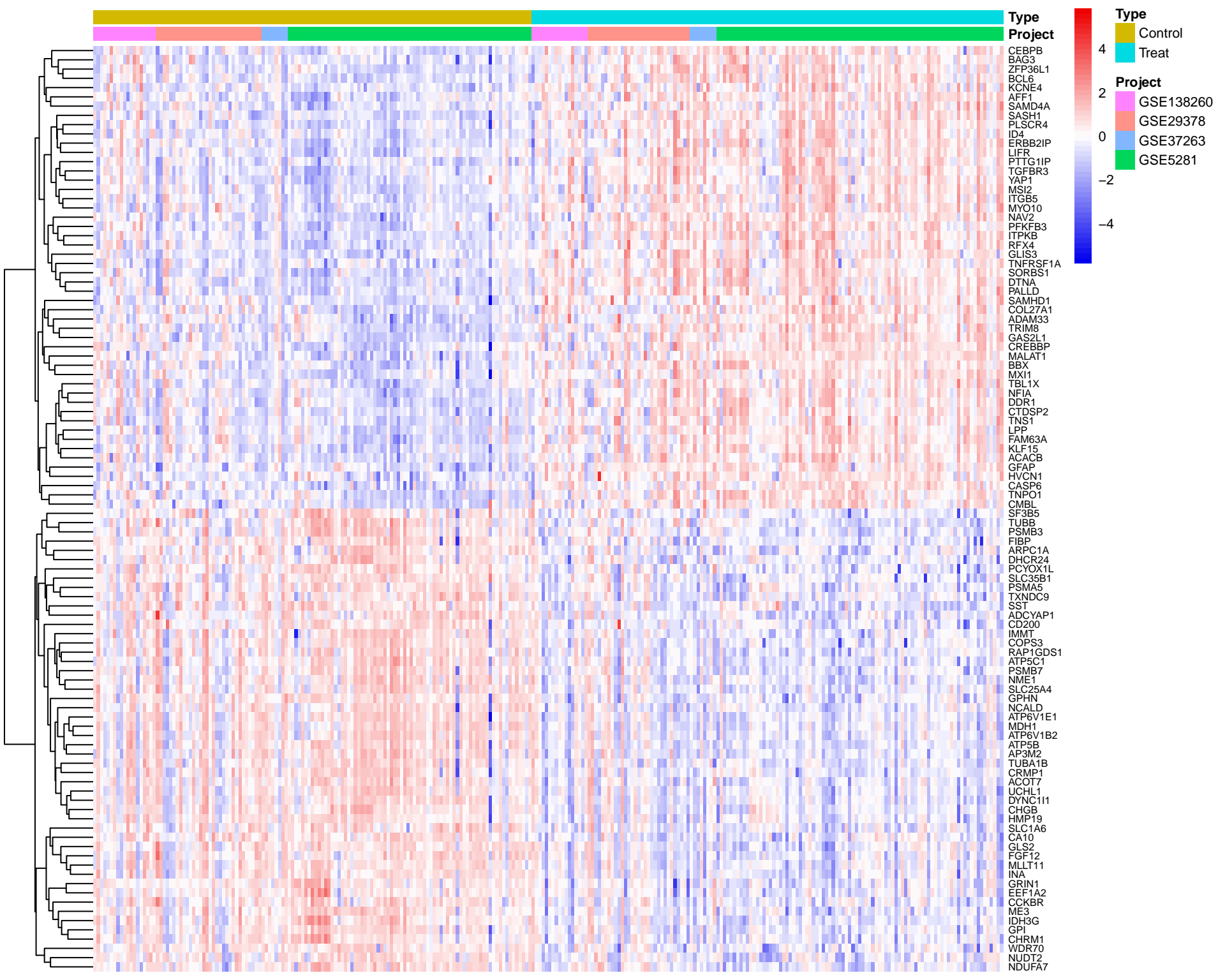

Supplement: Supplementary file 3 [file Data_Sheet_2.ZIP › Figure/Figure3.pdf]

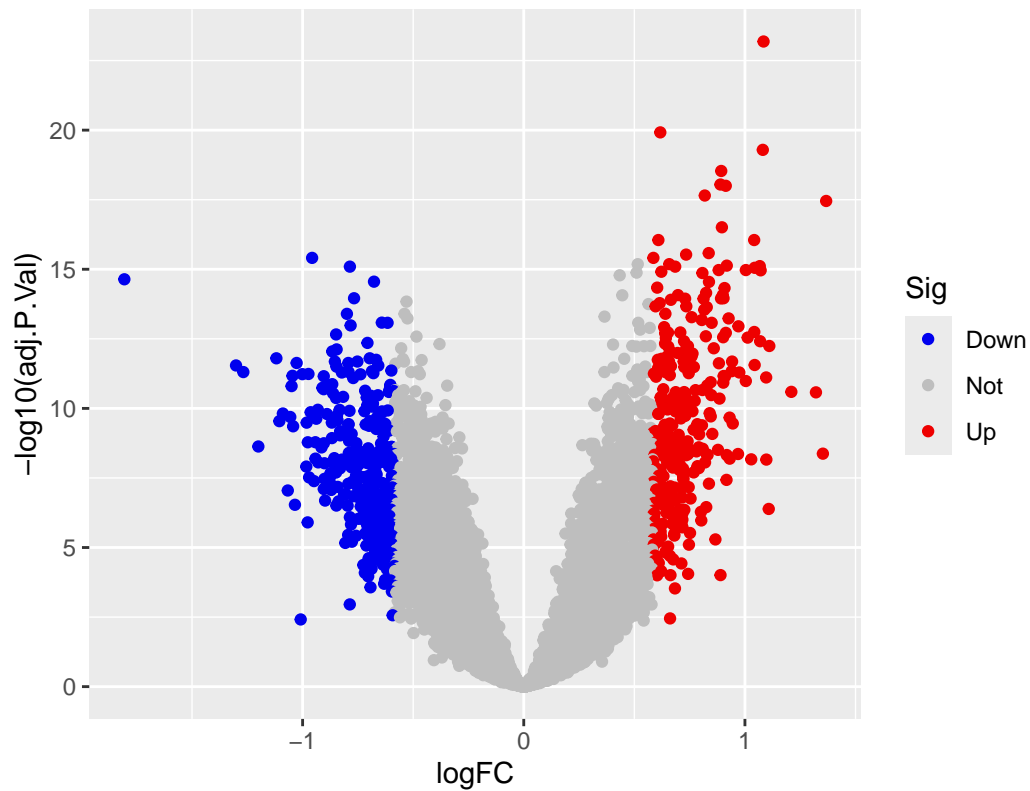

Supplement: Supplementary file 3 [file Data_Sheet_2.ZIP › Figure/Figure4.pdf]

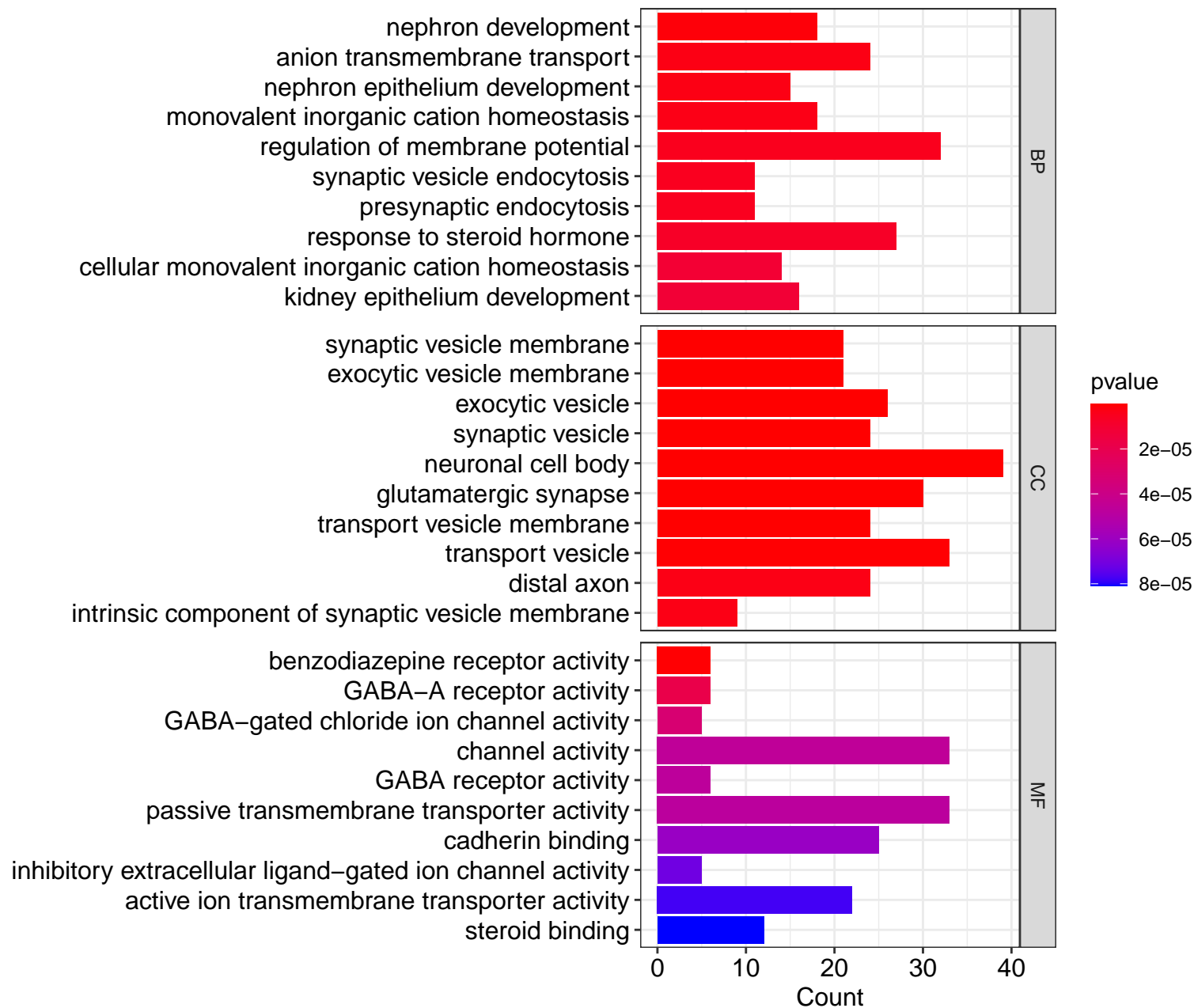

Supplement: Supplementary file 3 [file Data_Sheet_2.ZIP › Figure/Figure5.pdf]

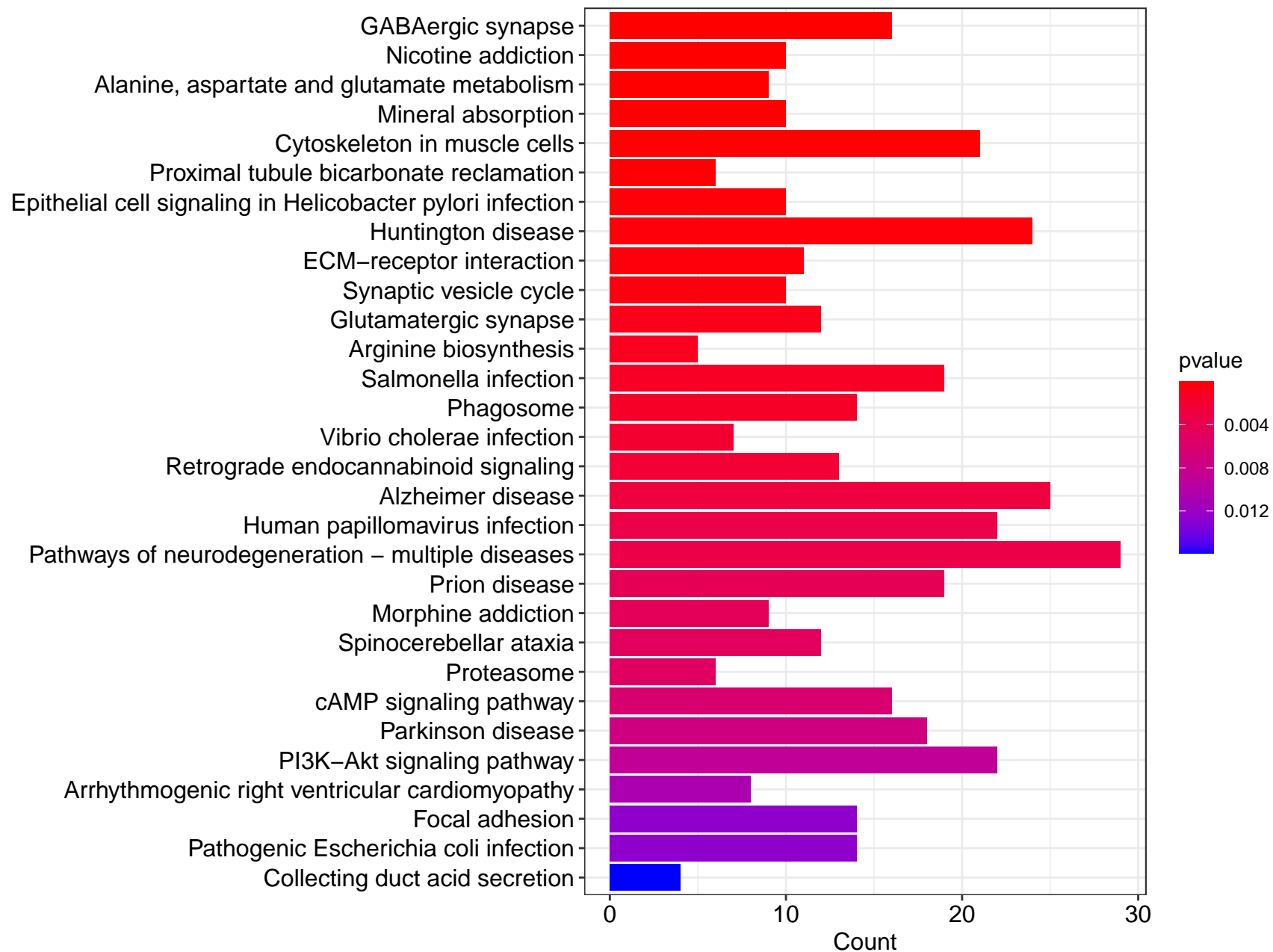

Supplement: Supplementary file 3 [file Data_Sheet_2.ZIP › Figure/Figure6.pdf]

DEG\_up

MR\_or>1

289

5

156

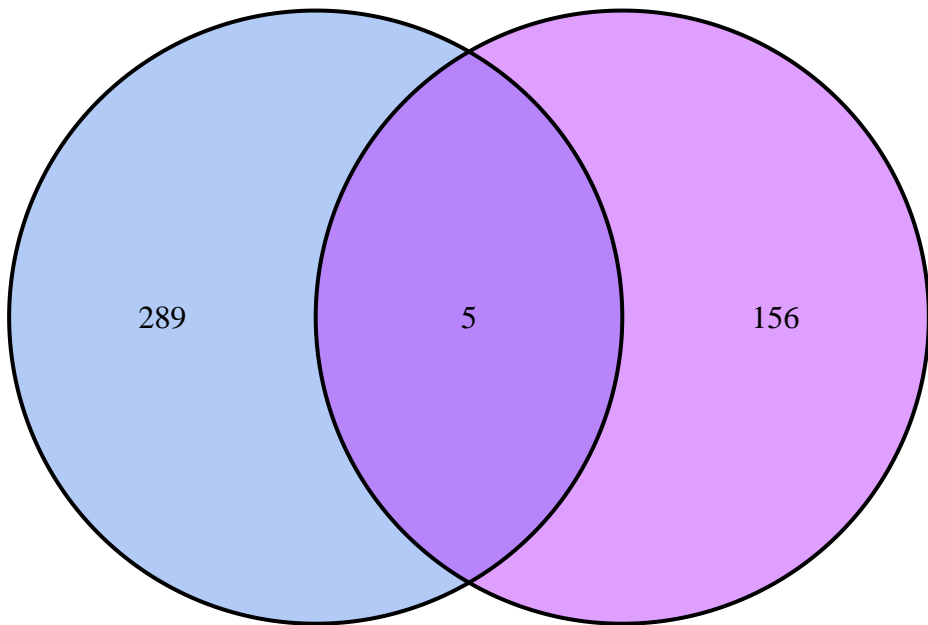

Supplement: Supplementary file 3 [file Data_Sheet_2.ZIP › Figure/Figure7A.pdf]

DEG\_down

MR\_or<1

325

5

183

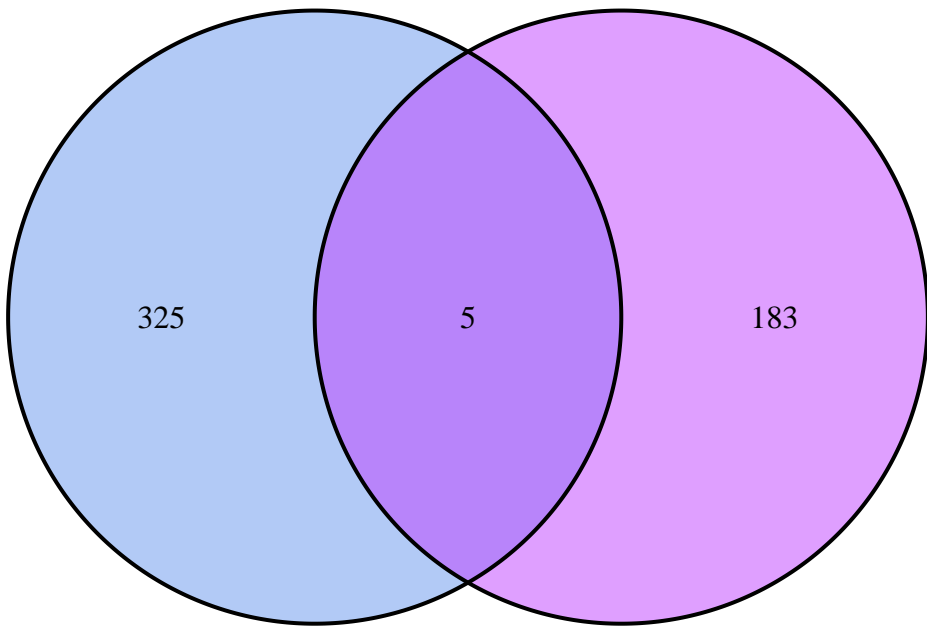

Supplement: Supplementary file 3 [file Data_Sheet_2.ZIP › Figure/Figure7B.pdf]

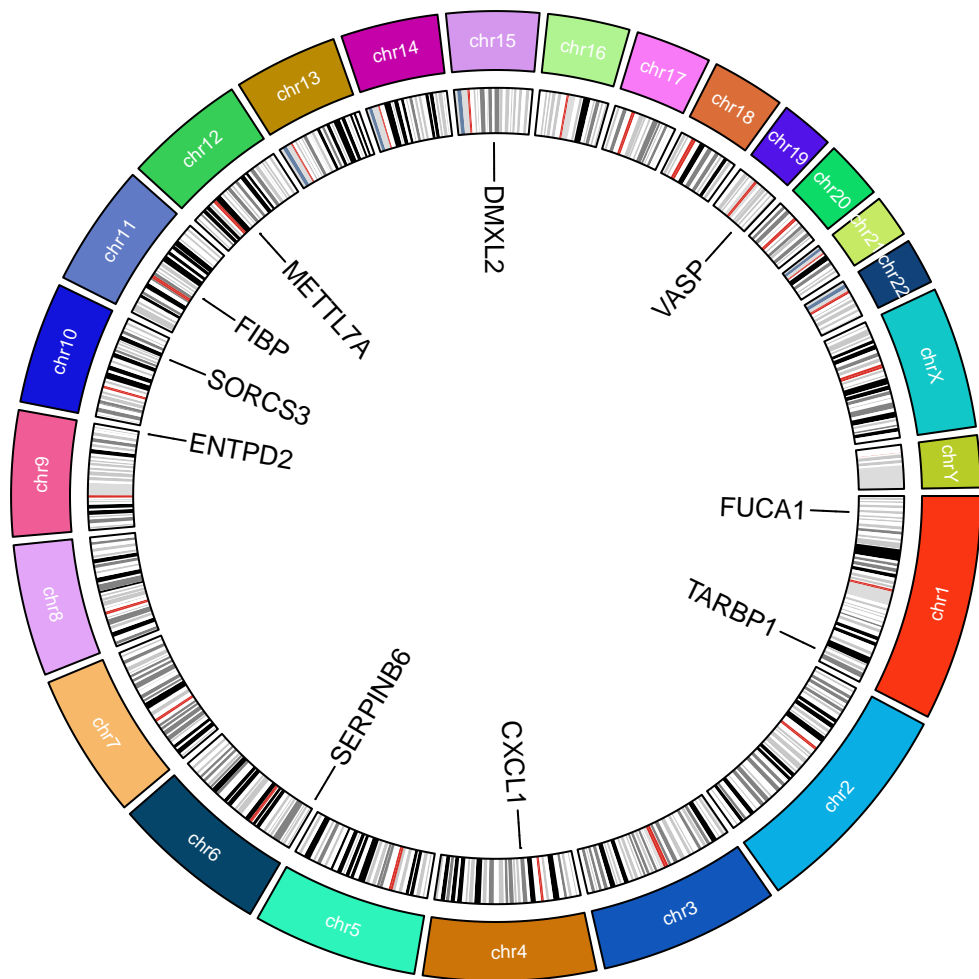

Supplement: Supplementary file 3 [file Data_Sheet_2.ZIP › Figure/Figure9.pdf]

# Enriched in high FUCA1 group

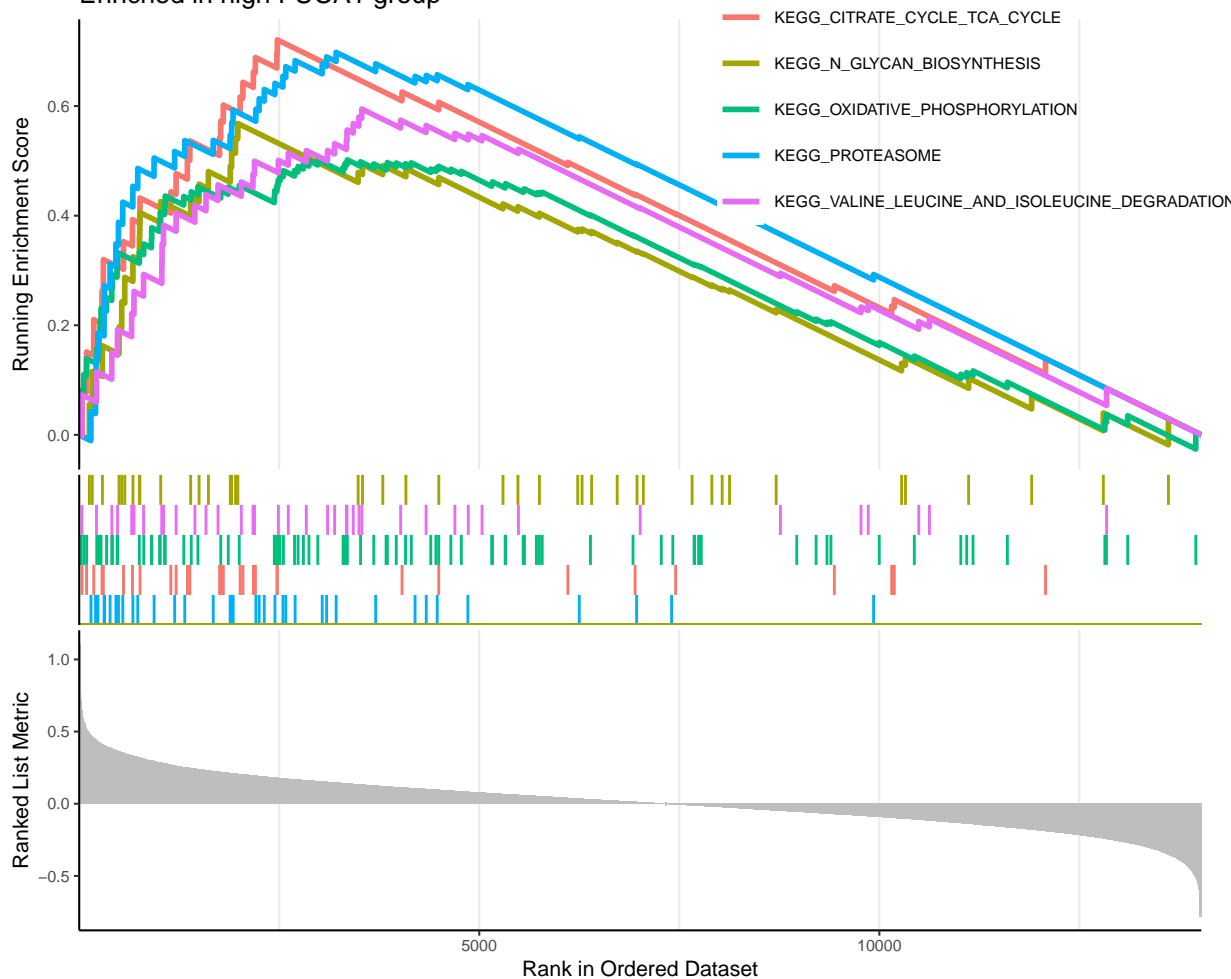

Supplement: Supplementary file 3 [file Data_Sheet_2.ZIP › Figure/FUCA1-GSEA.highExp.pdf]

# Enriched in high ENTPD2 group

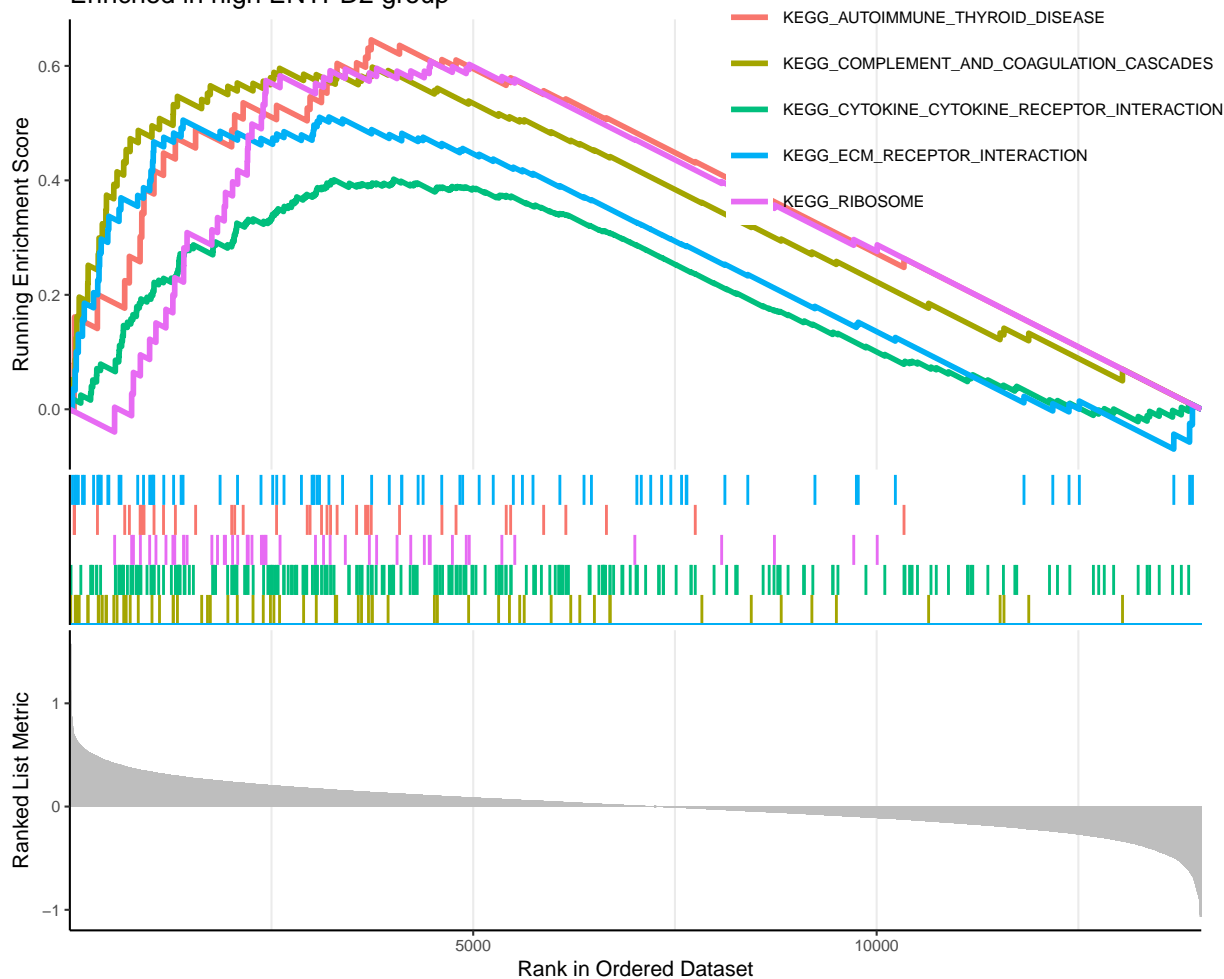

Supplement: Supplementary file 3 [file Data_Sheet_2.ZIP › Figure/GSEA.highExp.pdf]

# Enriched in high METTL7A group

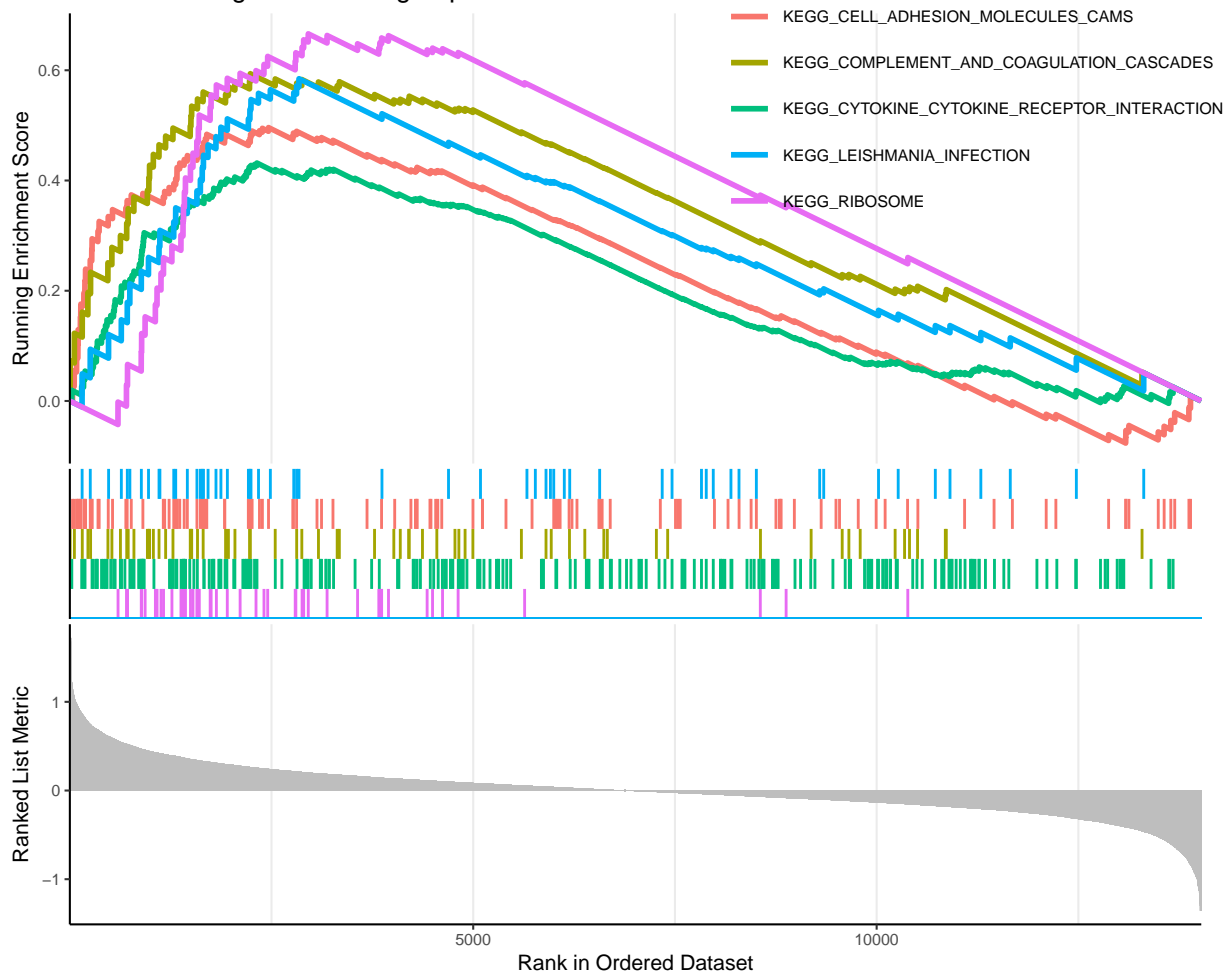

Supplement: Supplementary file 3 [file Data_Sheet_2.ZIP › Figure/METTL7A-GSEA.highExp.pdf]

# Enriched in high SERPINB6 group

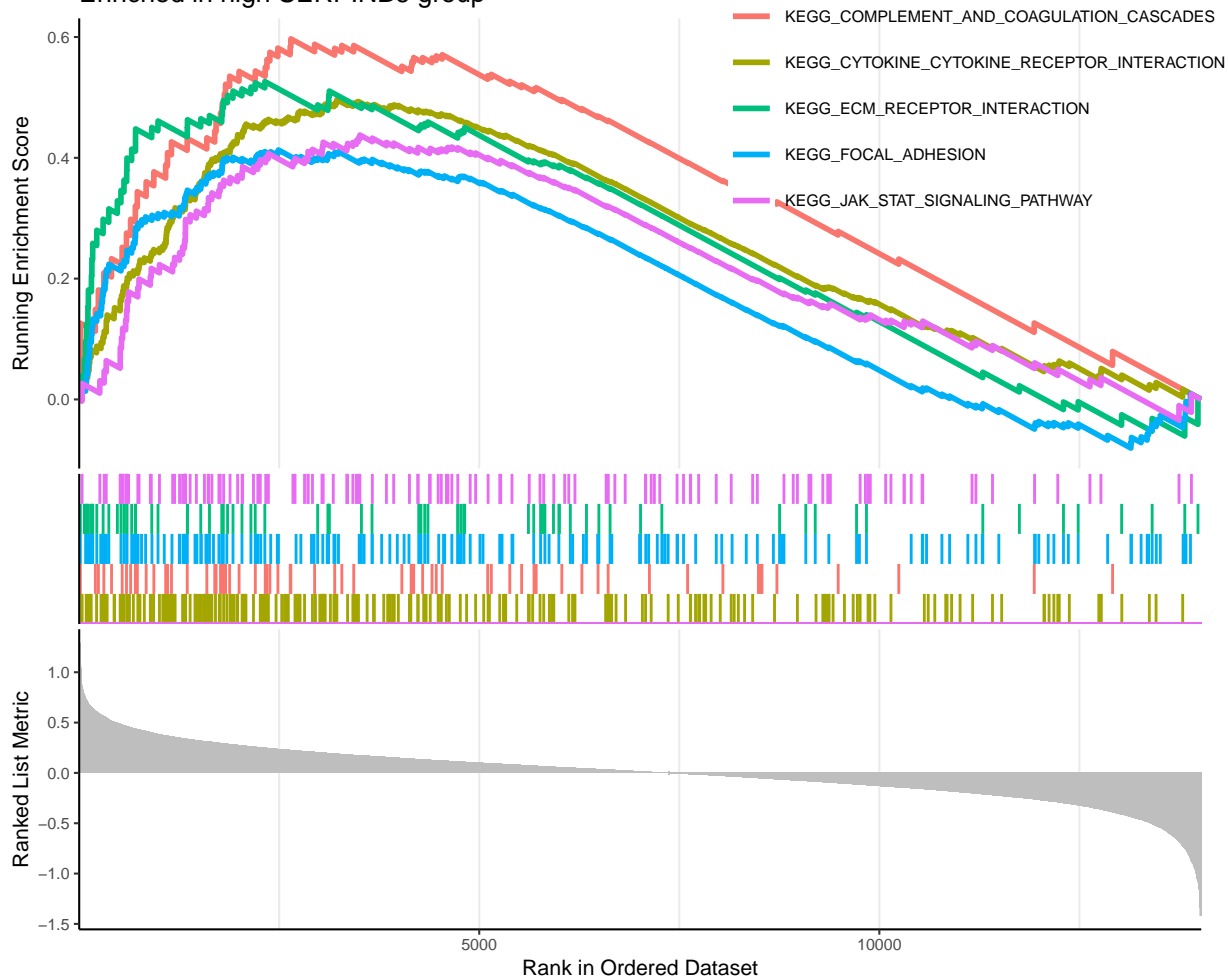

Supplement: Supplementary file 3 [file Data_Sheet_2.ZIP › Figure/SERPINB6-GSEA.highExp.pdf]

# Enriched in high SORCS3 group

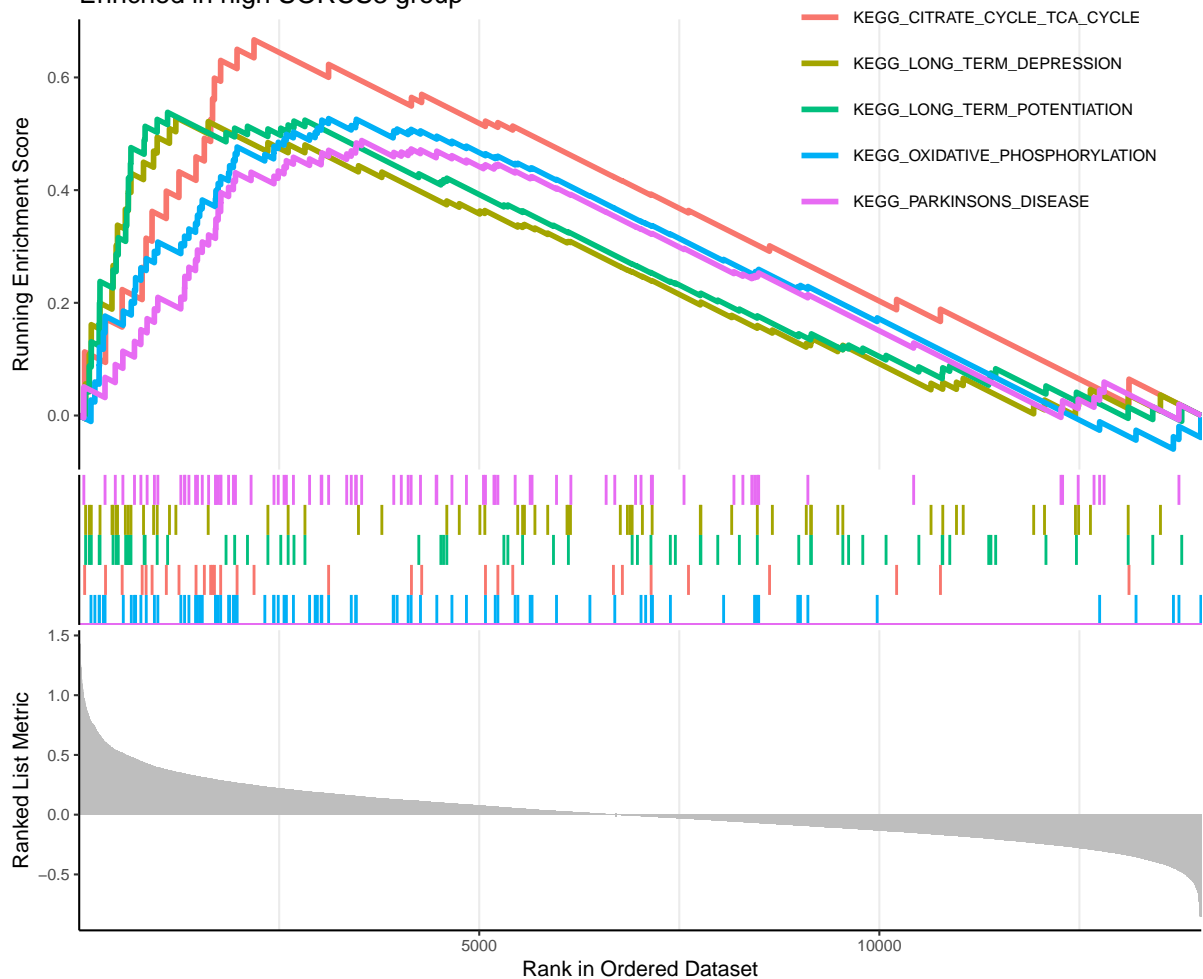

Supplement: Supplementary file 3 [file Data_Sheet_2.ZIP › Figure/SORCS3-GSEA.highExp.pdf]

# Enriched in high TARBP1 group

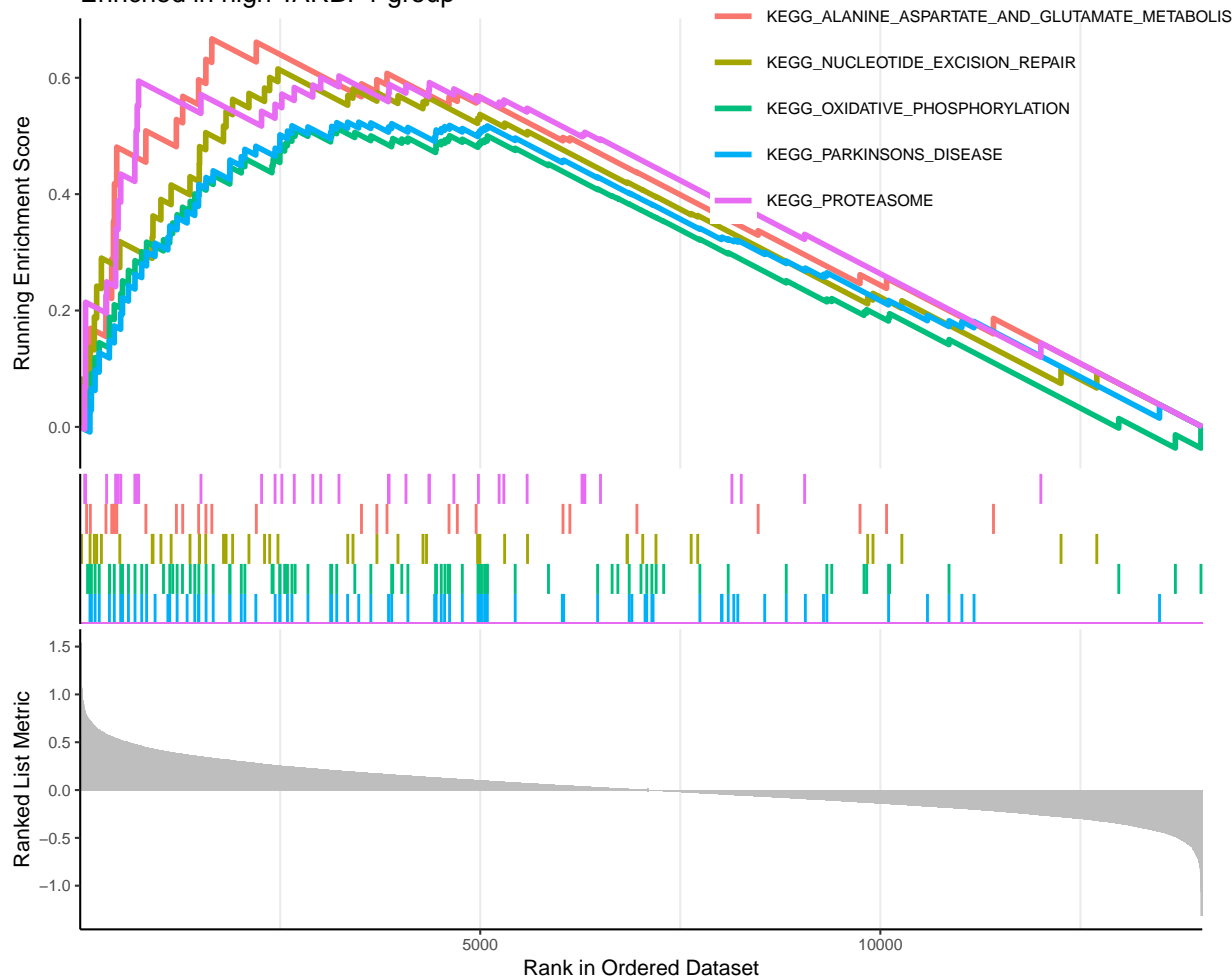

Supplement: Supplementary file 3 [file Data_Sheet_2.ZIP › Figure/TARBP1-GSEA.highExp.pdf]

# Enriched in high VASP group

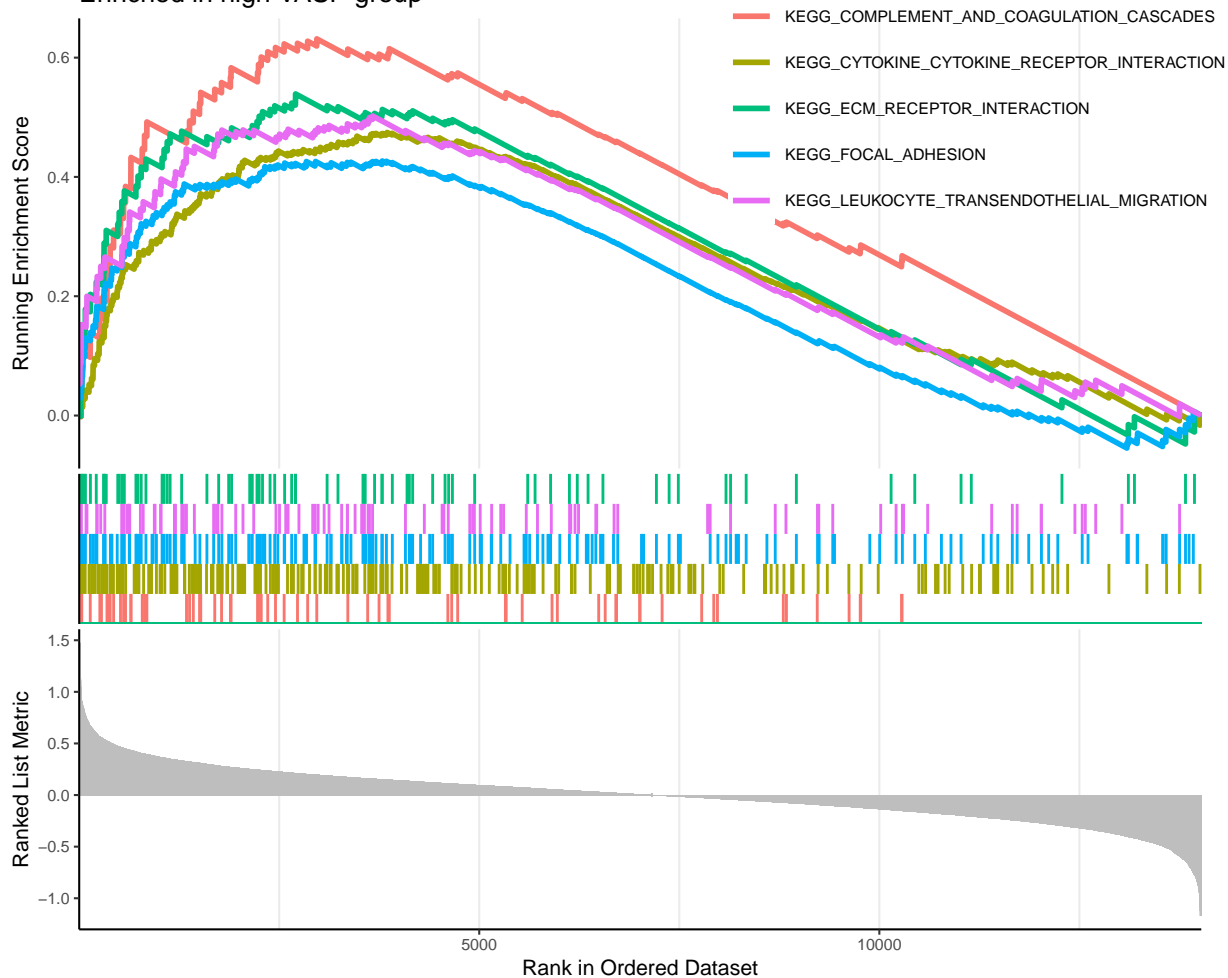

Supplement: Supplementary file 3 [file Data_Sheet_2.ZIP › Figure/VASP-GSEA.highExp.pdf]
